# Intensity-modulated fractionated radiotherapy versus stereotactic body radiotherapy for prostate cancer (PACE-B): acute toxicity findings from an international, randomised, open-label, phase 3, non-inferiority trial

**DOI:** 10.1016/S1470-2045(19)30569-8

**Published:** 2019-11

**Authors:** Douglas H Brand, Alison C Tree, Peter Ostler, Hans van der Voet, Andrew Loblaw, William Chu, Daniel Ford, Shaun Tolan, Suneil Jain, Alexander Martin, John Staffurth, Philip Camilleri, Kiran Kancherla, John Frew, Andrew Chan, Ian S Dayes, Daniel Henderson, Stephanie Brown, Clare Cruickshank, Stephanie Burnett, Aileen Duffton, Clare Griffin, Victoria Hinder, Kirsty Morrison, Olivia Naismith, Emma Hall, Nicholas van As, PACE Trial Investigators, D Dodds, E Lartigau, S Patton, A Thompson, M Winkler, P Wells, T Lymberiou, D Saunders, M Vilarino-Varela, P Vavassis, T Tsakiridis, R Carlson, G Rodrigues, J Tanguay, S Iqbal, M Winkler, S Morgan, A Mihai, A Li, O Din, M Panades, R Wade, Y Rimmer, J Armstrong, M Panades, N Oommen

**Affiliations:** a The Royal Marsden Hospital, London, UK; b Mount Vernon Cancer Centre, Northwood, UK; c The James Cook University Hospital, Middlesbrough, UK; d Odette Cancer Centre, Sunnybrook Health Sciences Centre, Toronto, ON, Canada; e University Hospitals Birmingham, Birmingham, UK; f The Clatterbridge Cancer Centre, Birkenhead, UK; g Queen's University Belfast, Belfast, UK; h Cambridge University Hospitals NHS Foundation Trust, Cambridge, UK; i Cardiff University, Cardiff, UK; j Churchill Hospital, Oxford, UK; k University Hospitals of Leicester, Leicester, UK; l Freeman Hospital, Newcastle, UK; m University Hospitals Coventry & Warwickshire, Coventry, UK; n Department of Oncology, McMaster University, Hamilton, ON, Canada; o Beatson West of Scotland Cancer Centre, Glasgow, UK; p The Institute of Cancer Research, London, UK

## Abstract

**Background:**

Localised prostate cancer is commonly treated with external-beam radiotherapy. Moderate hypofractionation has been shown to be non-inferior to conventional fractionation. Ultra-hypofractionated stereotactic body radiotherapy would allow shorter treatment courses but could increase acute toxicity compared with conventionally fractionated or moderately hypofractionated radiotherapy. We report the acute toxicity findings from a randomised trial of standard-of-care conventionally fractionated or moderately hypofractionated radiotherapy versus five-fraction stereotactic body radiotherapy for low-risk to intermediate-risk localised prostate cancer.

**Methods:**

PACE is an international, phase 3, open-label, randomised, non-inferiority trial. In PACE-B, eligible men aged 18 years and older, with WHO performance status 0–2, low-risk or intermediate-risk prostate adenocarcinoma (Gleason 4 + 3 excluded), and scheduled to receive radiotherapy were recruited from 37 centres in three countries (UK, Ireland, and Canada). Participants were randomly allocated (1:1) by computerised central randomisation with permuted blocks (size four and six), stratified by centre and risk group, to conventionally fractionated or moderately hypofractionated radiotherapy (78 Gy in 39 fractions over 7·8 weeks or 62 Gy in 20 fractions over 4 weeks, respectively) or stereotactic body radiotherapy (36·25 Gy in five fractions over 1–2 weeks). Neither participants nor investigators were masked to allocation. Androgen deprivation was not permitted. The primary endpoint of PACE-B is freedom from biochemical or clinical failure. The coprimary outcomes for this acute toxicity substudy were worst grade 2 or more severe Radiation Therapy Oncology Group (RTOG) gastrointestinal or genitourinary toxic effects score up to 12 weeks after radiotherapy. Analysis was per protocol. This study is registered with ClinicalTrials.gov, NCT01584258. PACE-B recruitment is complete and follow-up is ongoing.

**Findings:**

Between Aug 7, 2012, and Jan 4, 2018, we randomly assigned 874 men to conventionally fractionated or moderately hypofractionated radiotherapy (n=441) or stereotactic body radiotherapy (n=433). 432 (98%) of 441 patients allocated to conventionally fractionated or moderately hypofractionated radiotherapy and 415 (96%) of 433 patients allocated to stereotactic body radiotherapy received at least one fraction of allocated treatment. Worst acute RTOG gastrointestinal toxic effect proportions were as follows: grade 2 or more severe toxic events in 53 (12%) of 432 patients in the conventionally fractionated or moderately hypofractionated radiotherapy group versus 43 (10%) of 415 patients in the stereotactic body radiotherapy group (difference −1·9 percentage points, 95% CI −6·2 to 2·4; p=0·38). Worst acute RTOG genitourinary toxicity proportions were as follows: grade 2 or worse toxicity in 118 (27%) of 432 patients in the conventionally fractionated or moderately hypofractionated radiotherapy group versus 96 (23%) of 415 patients in the stereotactic body radiotherapy group (difference −4·2 percentage points, 95% CI −10·0 to 1·7; p=0·16). No treatment-related deaths occurred.

**Interpretation:**

Previous evidence (from the HYPO-RT-PC trial) suggested higher patient-reported toxicity with ultrahypofractionation. By contrast, our results suggest that substantially shortening treatment courses with stereotactic body radiotherapy does not increase either gastrointestinal or genitourinary acute toxicity.

**Funding:**

Accuray and National Institute of Health Research.

## Introduction

Prostate cancer is the most common non-cutaneous malignancy among men living in developed countries.^1^, ^2^, ^3^ For patients with National Comprehensive Cancer Network (NCCN) low-risk or intermediate-risk disease,^4^ several management approaches can be considered, including external-beam radiotherapy (EBRT), brachytherapy, surgery, and—for some—active surveillance. Data from the randomised ProtecT trial, which compared surgery, EBRT, and active monitoring, have given reassurance that cancer outcomes are similar for low-risk and intermediate-risk disease, regardless of the management option used.^5^ Therefore, side-effects might influence decision making, with gastrointestinal, genitourinary, and sexual side-effects being common concerns.^6^ Additionally, the tolerability of treatment for a given patient is crucial, with anaesthetic and intra-operative risks balanced against the inconvenience of multiweek courses of EBRT.

**Research in context**

**Evidence before this study**

At the time of initiation of this study on Jan 25, 2012, to our knowledge there were no published randomised controlled trials of ultra-hypofractionated stereotactic body radiotherapy compared with conventional fractionated or moderately hypofractionated radiotherapy for localised prostate cancer. Standard treatment was radiotherapy in 2 Gy per fraction, to a dose of 74 Gy or 78 Gy. A subsequent change of standard-of-care practice to moderate hypofractionation over the course of 2016 was reflected in the control group of this study. We searched PubMed using the terms [“SBRT” OR “Stereotactic Body Radiotherapy”] AND “Prostate” for studies published in English up to March 31, 2019. We searched the reference lists of the papers identified by our search, and supplemented the search with the authors' knowledge of the field. We identified 16 studies reporting acute toxicity outcomes from ultrahypofractionated radiotherapy to the prostate, including a randomised phase 3 study (HYPO-RT-PC). Grade 2 or worse acute toxicity estimates for ultra-hypofractionation were similar to standard fractionation, ranging from 4–24% for gastrointestinal toxicity and 4–40% for genitourinary toxicity.

**Added value of this study**

To our knowledge, this is the first published phase 3 randomised trial investigating acute toxicity after ultra-hypofractionated stereotactic body radiotherapy, delivered over five fractions, compared with standard fractionation schedules. Overall, this study showed similar acute toxicity for ultra-hypofractionation compared with standard fractionation, with only Common Terminology Criteria for Adverse Events grade 2 or more severe gastrointestinal toxicity being significantly worse. Proportions of patients with acute grade 3 toxicity were low, which adds to the body of evidence for low acute toxicity, as was also reported for seven-fraction hypofractionated radiotherapy in the HYPO-RT-PC trial.

**Implications of all the available evidence**

Ultra-hypofractionated radiotherapy over five fractions appears to be tolerable in the short term in men with low-risk of intermediate-risk prostate adenocarcinoma. The HYPO-RT-PC trial showed that a schedule of 42·7 Gy delivered every other day over 2·5 weeks (6·1 Gy per fraction) was non-inferior in terms of failure-free survival compared with conventional fractionation of 78 Gy over 8 weeks (2 Gy per fraction), with similar proportions of late toxicity in each group. Late toxicity and efficacy data for the PACE-B trial are awaited and are required before a new standard of care for localised prostate cancer can be recommended.

Hypofractionation—increasing the dose per fraction above the conventional 2 Gy, thus reducing the total fractions required—is an appealing approach. The key advantages are twofold. First, the greater fraction size sensitivity of prostate cancer (indicated by a lower α/β ratio^7^, ^8^, ^9^, ^10^), relative to the relevant late gastrointestinal and genitourinary side-effects, means that the therapeutic ratio might be improved by hypofractionation.^11^ Second, fewer fractions are needed with hypofractionation, allowing for quicker and more cost-effective EBRT treatment courses.^12^

Three major non-inferiority phase 3 randomised controlled trials have confirmed the safety and efficacy of moderate hypofractionation (2·5–3·0 Gy per fraction),^11^, ^13^, ^14^ which has gained acceptance as a standard-of-care option.^15^, ^16^ Although the proportions of patients with late toxicity were low, some intertrial differences in the proportion of patients who experienced acute toxicity were observed. The CHHiP trial reported significantly higher proportions of patients with peak acute Radiation Therapy Oncology Group (RTOG) grade 2 or worse gastrointestinal toxicity—38% in both hypofractionated groups—compared with conventional fractionation (25%; p<0·0001 for both comparisons).^11^ Similarly, the PROFIT trial reported a significantly (p=0·003) higher proportion of patients with cumulative acute RTOG grade 2 or worse gastrointestinal toxic effect proportions in the hypofractionated arm (16·7%) versus conventional fractionation (10·5%).^14^ For both trials, acute grade 2 or worse genitourinary toxic effects were similar between hypofractionated and conventionally fractionated groups. By contrast, the RTOG-0415 hypofractionation trial did not find a significant difference in acute gastrointestinal or genitourinary toxic effects between groups.^13^ Although more profound hypofractionation beyond 3·0 Gy per fraction would allow further reductions in the overall treatment time, the accelerated schedule might worsen acute toxicity, as seen in the CHHiP trial,^11^ potentially leading to late effects.^17^

Substantial evidence exists for the efficacy of ultra-hypofractionation, with over 6000 patients treated in prospective studies and excellent 5-year biochemical progression-free survival in a recent meta-analysis (95·3%, 95% CI 91·3–97·5).^18^ A phase 3 trial (HYPO-RT-PC) reported good biochemical progression-free survival and acceptable proportions of toxic effects for seven-fraction ultra-hypofractionated radiotherapy.^19^ To our knowledge, phase 3 randomised toxic effect data for five-fraction treatment have not previously been reported.

We report the acute toxicity findings (both clinician-reported and patient-reported) from the PACE-B randomised, controlled trial, which compared standard-of-care conventionally fractionated or moderately hypofractionated radiotherapy with five-fraction stereotactic body radiotherapy for low-risk to intermediate-risk localised prostate cancer.

## Methods

### Study design and participants

PACE-B is an international, phase 3, open-label, randomised, non-inferiority trial at 37 centres (appendix p 7) in three countries (UK, Ireland, and Canada) aiming to assess non-inferiority of stereotactic body radiotherapy compared with conventionally fractionated or moderately hypofractionated radiotherapy for biochemical or clinical failure.

The PACE study comprises multiple cohorts (PACE-A, PACE-B, and PACE-C) which were independently randomised. This study, PACE-B, recruited only patients suitable for radical radiotherapy, but not willing to have or not suitable for radical prostatectomy. Eligible patients were men aged at least 18 years, with WHO performance status of 0–2,^20^ life expectancy of at least 5 years, and histologically confirmed prostate adenocarcinoma. All patients had NCCN low-risk or intermediate-risk disease.^4^ Low-risk patients had cT1c–T2a (TNM 6th edition^21^), N0-X, M0-X, Gleason score 6 or less, and prostate-specific antigen (PSA) concentration less than 10 ng/mL. Intermediate-risk patients had at least one of the following criteria: T2c, Gleason score 7 (3 + 4 for PACE; Gleason 4 + 3 was excluded), and PSA 10–20 ng/mL. Distant staging was not mandated. A minimum of ten biopsy cores taken within the last 18 months before randomisation were required, except for those progressing on active surveillance, whose last biopsy was suitable for PACE-B and required treatment (eg, PSA or MRI progression). These patients were stratified as intermediate risk. No PSA adjustment was made for 5-α reductase inhibitor use at randomisation. Treating physicians had discretion to exclude patients for comorbid conditions that made radiotherapy inadvisable (eg, inflammatory bowel disease or substantial urinary tract symptoms). Detailed inclusion and exclusion criteria are in the protocol (appendix pp 82–83).

The trial was approved by the London Chelsea research ethics committee (reference 11/LO/1915) in the UK and the relevant institutional review boards in Ireland and Canada. PACE-B was conducted in accordance with the principles of Good Clinical Practice. All participants provided voluntary written informed consent.

### Randomisation and masking

Patients were randomly assigned (1:1) to conventionally fractionated or moderately hypofractionated radiotherapy or stereotactic body radiotherapy. Randomisation was done centrally by the Institute of Cancer Research Clinical Trials and Statistics Unit (ICR-CTSU), by telephone (UK and Ireland) or fax (Canada), with allocation by computer generated random permuted blocks (size four and six) and stratification by centre and risk group (low or intermediate). Sequence generation, enrolment, and trial group assignment were done by ICR-CTSU staff who were not involved in the clinical running of the trial or data collection. Participants and researchers were not masked to treatment assignment.

### Procedures

Before radiotherapy, three or more prostatic fiducial markers were strongly recommended for all participants to permit more accurate image-guided radiotherapy and CT or MRI fusion. Bowel preparation (enema) was suggested, along with moderate bladder filling. The radiotherapy planning CT scan took place at least 7 days after fiducial placement. A radiotherapy planning MRI scan was strongly recommended to be fused to the CT scan (preferably by fiducial match) for improved prostate anatomical definition. The clinical target volume (CTV) was the prostate only (low-risk patients) or prostate and proximal 1 cm of seminal vesicles (intermediate-risk patients). The recommended conventionally fractionated or moderately hypofractionated radiotherapy CTV to planning target volume (PTV) expansion was 5–9 mm isometric, except posteriorly 3–7 mm. The recommended stereotactic body radiotherapy CTV to PTV expansion was 4–5 mm isometric, except posteriorly 3–5 mm. Dose constraints were applied to organs at risk and were amended during the trial. A history of the constraints used with numbers of patients randomised to each iteration is presented in the appendix (pp 4–5). Additional detail on radiotherapy preparation and final dose constraints used from March 24, 2016, are in the protocol (appendix pp 93–100). Androgen deprivation therapy was not permitted.

The conventionally fractionated or moderately hypofractionated radiotherapy PTV dose was 78 Gy in 39 daily fractions or, following an approved protocol amendment (on March 24, 2016), 62 Gy in 20 daily fractions. This change followed the CHHiP trial data supporting moderate hypofractionation,^11^ but with a higher dose (62 Gy *vs* 60 Gy) because the PACE-B protocol prohibits androgen deprivation therapy. Data from the PROFIT trial which assessed 60 Gy in 20 fractions, without androgen deprivation therapy, were not available at that time.^14^ After the protocol amendment, centres were required to choose either 78 Gy in 39 fractions or 62 Gy in 20 fractions as their control treatment for all subsequent patients. The stereotactic body radiotherapy PTV dose was 36·25 Gy in five fractions over 1–2 weeks (ie, daily or alternate days, at centre discretion), with an additional secondary CTV dose target of 40 Gy. The CyberKnife treatment platform (Accuray; Sunnyvale, CA, USA) was initially mandatory for all stereotactic body radiotherapy; however, sponsorship changes prompted a protocol amendment (on Oct 24, 2014) permitting stereotactic body radiotherapy delivery on conventional linear accelerators. Detailed prescription objectives, along with minor variations permitted, are listed in the protocol (appendix pp 93–100).

Treatment was mandated to commence within 12 weeks of randomisation, with 8 weeks or less strongly recommended. Image-guided radiotherapy (preferably fiducial based) was mandated. For stereotactic body radiotherapy, intrafractional motion monitoring was permitted; otherwise, a repeat static image was required for stereotactic body radiotherapy fraction delivery extending beyond 3 min. A radiotherapy quality assurance programme was undertaken for each centre to ensure consistency with the trial protocol and quality of radiotherapy treatments (appendix p 113).

Participants were assessed on alternate weeks during conventionally fractionated or moderately hypofractionated radiotherapy and on the final fraction for stereotactic body radiotherapy, and at weeks 2, 4, 8, and 12 after the end of treatment for all patients in both groups. Two clinician-reported outcomes were collected—RTOG (gastrointestinal and genitourinary domains) at baseline and every visit and Common Terminology Criteria for Adverse Events (CTCAE version 4.03) at baseline and follow-up weeks 2, 4, 8, and 12, with additional end-of-treatment assessment for patients in the stereotactic body radiotherapy group. Specific CTCAE items in the gastrointestinal composite are anal pain, colitis, constipation, diarrhoea, diverticulitis, faecal incontinence, fistula, gastrointestinal pain, haemorrhoids, gastrointestinal haemorrhage, proctitis, rectal pain, gastrointestinal unspecified, and rectal prolapse. Specific CTCAE items in the genitourinary composite are bladder spasm, cystitis, haematuria, prostatic obstruction, urinary frequency, urinary incontinence, urinary retention, urinary urgency, and urethral stricture. We used paper questionnaires to collect four patient-reported outcome measures—expanded prostate cancer index composite short form (EPIC-26) and the Vaizey faecal incontinence score, at baseline and weeks 4 and 12; international prostate symptom score (IPSS), at baseline and weeks 2, 4, 8, and 12; and the international index of erectile function 5-question (IIEF-5) score, at baseline and week 12. Subsequent follow-up is ongoing (and will continue until all patients have reached 10 years), with the full schedule, along with criteria for removal of patients from the study, available in the protocol (appendix pp 84–92). Regular toxicity and patient-reported outcome assessment occurs during follow up. Study recruitment is complete.

The EPIC tool gives a measure of patient-reported quality of life in genitourinary, gastrointestinal, sexual, and general domains. The Vaizey questionnaire measures patient quality of life relating to faecal incontinence and the IPSS records patient experience of various facets of urinary function. For each scale, the baseline, worst, worst above baseline, and week 12 (residual) toxic effects were of interest, with exact definitions detailed in the statistical analysis plan (appendix pp 145–47).

### Outcomes

The primary endpoint of PACE-B is freedom from biochemical or clinical failure, the data for which is not yet mature. This acute toxicity report is a prespecified subanalysis of the PACE-B trial. A statistical analysis plan for this substudy (appendix pp 129–158) was prospectively written, with worst grade 2 or worse RTOG toxic effects score, up to week 12 follow-up after radiotherapy finished, for both gastrointestinal and genitourinary systems, as coprimary sub-study endpoints.

Separately for gastrointestinal and genitourinary systems, the numerator was patients with recorded RTOG grade 2 or worse toxic effects at any point after baseline up to week 12 after radiotherapy. The denominator was all patients with at least one RTOG score completed after baseline up to week 12 after radiotherapy. Patients were recorded as missing if no such score was returned. This endpoint was pragmatically chosen, as only RTOG assessments were done for patients in the conventionally fractionated or moderately hypofractionated group during radiotherapy. PACE-B secondary endpoints were acute toxicity (CTCAE), late toxicity (CTCAE and RTOG), progression-free survival, disease-specific survival, overall survival, distant progression, commencement of hormone therapy, and acute and late patient-reported toxicity (EPIC, IPSS, IIEF-5, and Vaizey scales). All but the physician-reported and patient-reported toxicity outcomes will be reported elsewhere.

### Statistical analysis

For this acute toxicity analysis, patients were analysed per protocol, with those receiving one or more fractions of conventionally fractionated or moderately hypofractionated radiotherapy or stereotactic body radiotherapy included. Patients who did not receive radiotherapy were excluded from this analysis. Patients receiving both conventionally fractionated or moderately hypofractionated radiotherapy and stereotactic body radiotherapy fractions were excluded unless the reason was toxicity-related, where analysis was on the first treatment fraction received. The PACE-B trial targeted recruitment of 858 patients to exclude a hazard ratio [HR] of 1·45 in biochemical or clinical failure at 5 years, with consideration given to also excluding a 6% increase in grade 2 gastrointestinal or genitourinary late toxicity at 2 years (appendix pp 107–08). For this acute toxicity substudy, we assumed conventionally fractionated or moderately hypofractionated radiotherapy group acute RTOG grade 2 or worse toxic effect proportions of 25% (gastrointestinal) and 40% (genitourinary), as per the CHHiP trial.^11^ With two-sided α=0·025 for each endpoint, we estimated that ongoing PACE-B recruitment would provide 83% power to exclude a 10% increase in acute gastrointestinal toxic effects and 84·5% power to exclude an 11% increase in acute genitourinary toxic effects for the stereotactic body radiotherapy group (appendix pp 139–40).

We used the χ^2^ test to compare treatment groups for the coprimary endpoints. Secondary endpoints were compared using appropriate statistical tests (appendix pp 145–47). To reduce the effect of multiple comparisons, p<0·001 was considered significant for secondary comparisons. The different durations of radiotherapy (conventionally fractionated or moderately hypofractionated radiotherapy 4 or 7·8 weeks; stereotactic body radiotherapy 1 or 2 weeks) led to differing timepoints of toxicity assessment. RTOG (assessed during radiotherapy) and CTCAE (assessed at end of radiotherapy for stereotactic body radiotherapy) graphical presentation is therefore protrayed as four different groups and also displayed grouped as 1–2 weeks and 4–7·5 weeks (interpolation detailed in appendix p 6). We calculated confidence intervals for the difference in proportions by normal approximation.

We rescaled EPIC-26 scores to a 0–100 point scale, with higher scores representing better quality of life.^22^ Subdomains were scored if sufficient questions were completed as follows: urinary incontinence (four of four questions), urinary obstructive (four of four questions), bowel (five of six questions), sexual (five of six questions), and hormonal (four of five questions).^22^ A clinically important point reduction in EPIC-26 subdomain score was as follows: urinary incontinence (8 points), urinary obstruction (6 points), bowel (5 points), sexual (11 points), and hormonal (5 points).^23^ IPSS severity categories were assessed as none (0 points), mild (1–7 points), moderate (8–19 points), and severe (20–35 points).^24^

Exploratory examination of CyberKnife versus standard linear accelerators for patients undergoing stereotactic body radiotherapy was prespecified in the protocol when amendment permitted standard linear accelerators (Aug 5, 2014). The prespecified statistical analysis plan called for a multivariate analysis, which will be published subsequently, but a post-hoc decision due to the paucity of published non-CyberKnife toxicity data was made to analyse the worst RTOG grade 2 or more severe gastrointestinal and genitourinary toxic effects for patients undergoing stereotactic body radiotherapy, split by CyberKnife and non-CyberKnife use, interpreted at a significant p-value of 0·001. Since centre-level effects could influence this non-randomised analysis (eg, variation in toxic effect reporting), we did similar analysis for patients undergoing conventionally fractionated or moderately hypofractionated radiotherapy, separated by whether their centre used CyberKnife or non-CyberKnife for stereotactic body radiotherapy treatments.

The study was overseen by a trial steering committee and an independent data monitoring committee (IDMC; appendix p 3). Analyses are based on a snapshot of data taken on May 28, 2019, and were done with STATA version 15.1. The IDMC gave approval for release of acute toxicity results before release of primary endpoint (efficacy) results. The PACE study is prospectively registered at ClinicalTrials.gov, NCT01584258.

### Role of the funding source

The funder of the study had no role in study design, data collection, data analysis, data interpretation, or writing of the report. The corresponding author had full access to all the data in the study and had final responsibility for the decision to submit for publication.

## Results

**Figure 1:**
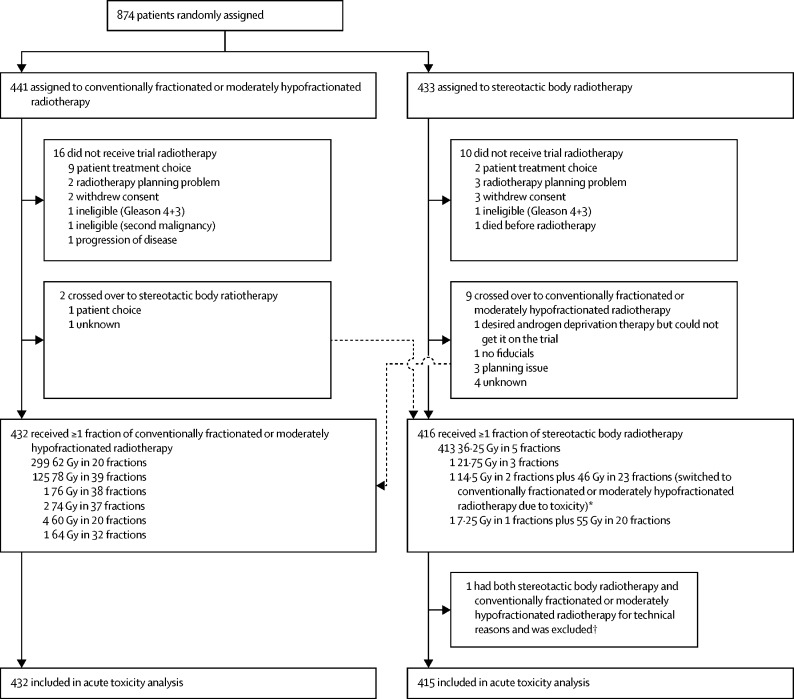
Trial profile Crossovers between treatment groups were analysed per-protocol for this acute toxicity substudy. Dose fractionation regimens administered within each group are shown. *One patient who received two fractions of stereotactic body radiotherapy then developed grade 3 toxicity (urosepsis) and switched to conventionally fractionated or moderately hypofractionated radiotherapy (further 46 Gy in 23 fractions) was not excluded from the toxicity analysis because he had toxic effects after two fractions of sterotactic body radiotherapy. †One patient who received a single incomplete fraction of stereotactic body radiotherapy (<7·25 Gy, set-up issues) and switched to conventionally fractionated or moderately hypofractionated radiotherapy (further 55 Gy in 20 fractions) was excluded.

Between Aug 7, 2012, and Jan 4, 2018, we randomly assigned 874 men to conventionally fractionated or moderately hypofractionated radiotherapy (n=441) or stereotactic body radiotherapy (n=433; figure 1). Median follow-up was 12 weeks (IQR 12–12), matching the time period authorised for data release. 11 patients received non-protocol regimens due to crossing over treatment groups (figure 1; appendix p 8); one cross over was toxicity-related (a patient in the stereotactic body radiotherapy group with grade 3 urinary toxicity), meaning that 432 patients received at least one fraction of conventionally fractionated or moderately hypofractionated radiotherapy and 416 patients received at least one fraction of stereotactic body radiotherapy. Two men received both stereotactic body radiotherapy and conventionally fractionated or moderately hypofractionated radiotherapy treatments—one patient who received two fractions of stereotactic body radiotherapy (14·5 Gy) then developed grade 3 toxicity (urosepsis) and switched to conventionally fractionated or moderately hypofractionated radiotherapy (further 46 Gy in 23 fractions) was not excluded from the toxicity analysis because he had toxic effects after two fractions of sterotactic body radiotherapy and one patient who received a single incomplete fraction of stereotactic body radiotherapy (<7·25 Gy, set-up issues) and switched to conventionally fractionated or moderately hypofractionated radiotherapy (further 55 Gy in 20 fractions) was excluded (figure 1).

Baseline characteristics for each per-protocol treatment group were similar (table 1). Four (21%) of 19 patients on a 5-α reductase inhibitor at baseline had a PSA value of 10–20 ng/mL. Radiotherapy delivery techniques (planning, image-guided radiotherapy, and margins) differed between arms, as expected, although recorded supportive prescribing appeared to be similar (appendix pp 9–11). Despite fiducial recommendations for both groups, more patients in the stereotactic body radiotherapy group received fiducial markers (303 [73%] of 415 patients) than did patients in the conventionally fractionated or moderately hypofractionated radiotherapy group (245 [57%] of 432 patients). RTOG and CTCAE form completion was excellent at all timepoints (appendix p 12). Patient illness caused non-completion of three RTOG forms (two in the conventionally fractionated or moderately hypofractionated radiotherapy group and one in the stereotactic body radiotherapy group) and one CTCAE form in the stereotactic body radiotherapy group. Patient-reported outcome assessment completion varied by scale (appendix pp 13–14). One patient randomly assigned to stereotactic body radiotherapy died because of myocardial infarction before receiving trial treatment and was excluded from per-protocol analyses; no other deaths were reported up to 12 weeks after completion of radiotherapy.

**Table 1:** Baseline characteristics

		**Conventionally fractionated or moderately hypofractionated radiotherapy group (n=432)**	**Stereotactic body radiotherapy group (n=415)**
Age (years)		69·7 (65·6–73·9)	69·6 (65·3–73·8)
Ethnicity			
	Black	25 (6%)	35 (8%)
	East Asian	3 (1%)	4 (1%)
	Mixed heritage	2 (<1%)	2 (<1%)
	South Asian	9 (2%)	19 (5%)
	White	386 (89%)	352 (85%)
	Other	7 (2%)	3 (1%)
Family history of prostate cancer			
	No	321 (74%)	300 (72%)
	Yes	85 (20%)	85 (20%)
	Unknown	26 (6%)	30 (7%)
WHO performance status			
	0	382 (88%)	372 (90%)
	1	48 (11%)	43 (10%)
	2	2 (<1%)	0
National Comprehensive Cancer Network risk score			
	Low	38 (9%)	30 (7%)
	Intermediate	394 (91%)	385 (93%)
T stage			
	T1c	78 (18%)	76 (18%)
	T2a	130 (30%)	105 (25%)
	T2b	57 (13%)	81 (20%)
	T2c	167 (39%)	153 (37%)
Gleason grade			
	3 + 3	84 (19%)	61 (15%)
	3 + 4	348 (81%)	354 (85%)
Pre-treatment PSA (ng/mL)			
	Mean	8·7 (3·7)	8·6 (4·0)
	Median	8·0 (6·3–11·0)	8·0 (5·5–11·0)
	<10	299 (69%)	283 (68%)
	10–20	133 (31%)	132 (32%)
Pre-treatment testosterone (nmol/L)			
	<1·7	0	2 (<1%)
	≥1·7	391 (91%)	376 (91%)
	Unknown	41 (9%)	37 (9%)
Active surveillance before trial enrolment			
	Yes	160 (37%)	146 (35%)
	No	258 (60%)	256 (62%)
	Unknown	14 (3%)	13 (3%)
Prostate volume (mL)			
	<40	153 (35%)	160 (39%)
	40–<80	200 (46%)	170 (41%)
	≥80	16 (4%)	21 (5%)
	Unknown	63 (15%)	64 (15%)
α blockers at randomisation			
	Yes	68 (16%)	67 (16%)
	No	361 (84%)	344 (83%)
	Unknown	3 (1%)	4 (1%)
Aspirin at randomisation			
	Yes	74 (17%)	63 (15%)
	No	355 (82%)	347 (84%)
	Unknown	3 (1%)	5 (1%)
Statin at randomisation			
	Yes	153 (35%)	126 (30%)
	No	275 (64%)	283 (68%)
	Unknown	4 (1%)	6 (1%)
Anticholinergic for bladder symptoms at randomisation			
	Yes	16 (4%)	10 (2%)
	No	414 (96%)	400 (96%)
	Unknown	2 (<1%)	5 (1%)
5-α reductase inhibitor at randomisation			
	Yes	9 (2%)	10 (2%)
	No	416 (96%)	387 (93%)
	Unknown	7 (2%)	18 (4%)
Phosphodiesterase-5 inhibitor at randomisation			
	Yes	12 (3%)	6 (1%)
	No	412 (95%)	392 (94%)
	Unknown	8 (2%)	17 (4%)

Data are median (IQR), n (%), or mean (SD). PSA=prostate-specific antigen.

Worst RTOG grade 2 or more severe gastrointestinal toxic effects did not differ significantly between conventionally fractionated or moderately hypofractionated radiotherapy (53 [12%] of 432 patients) and stereotactic body radiotherapy (43 [10%] of 415 patients; difference −1·9 percentage points, 95% CI −6·2 to 2·4; p=0·38; table 2).

**Table 2:** Radiation Therapy Oncology Group adverse events

	**Conventionally fractionated or moderately hypofractionated radiotherapy (n=432)**				**Stereotactic body radiotherapy (n=415)**			
	Grade 1	Grade 2	Grade 3	Grade 4	Grade 1	Grade 2	Grade 3	Grade 4
Gastrointestinal	264 (61%)	49 (11%)	4 (1%)	0	219 (53%)	42 (10%)	1 (<1%)	0
Genitourinary	254 (59%)	111 (26%)	6 (1%)	1 (<1%)	236 (57%)	86 (21%)	8 (2%)	2 (<1%)

Data are n (%). No death due to adverse events were reported.

Worst RTOG grade 2 or more severe genitourinary toxic effects also did not differ significantly between conventionally fractionated or moderately hypofractionated radiotherapy (118 [27%] of 432 patients) and stereotactic body radiotherapy (96 [23%] of 415 patients; difference −4·2 percentage points, 95% CI −10·0 to 1·7; p=0·16).

For RTOG secondary endpoints, we observed no significant differences between conventionally fractionated or moderately hypofractionated radiotherapy and stereotactic body radiotherapy for any comparison for gastrointestinal toxic effects (appendix p 15), including worst RTOG gastrointestinal toxic effects of grade 3 or more severe (four [1%] of 432 patients in the conventionally fractionated or moderately hypofractionated radiotherapy group *vs* one [<1%] of 415 patients in the stereotactic body radiotherapy group; difference −0·7 percentage points, 95% CI −1·7 to 0·3; p=0·37), nor for genitourinary toxic effects (appendix p 16), including worst RTOG genitourinary toxic effects of grade 3 or more severe (seven [2%] of 432 patients in the conventionally fractionated or moderately hypofractionated radiotherapy group *vs* ten [2%] of 415 patients in the stereotactic body radiotherapy group; difference 0·8 percentage points, −1·1 to 2·7; p=0·47).

**Figure 2:**
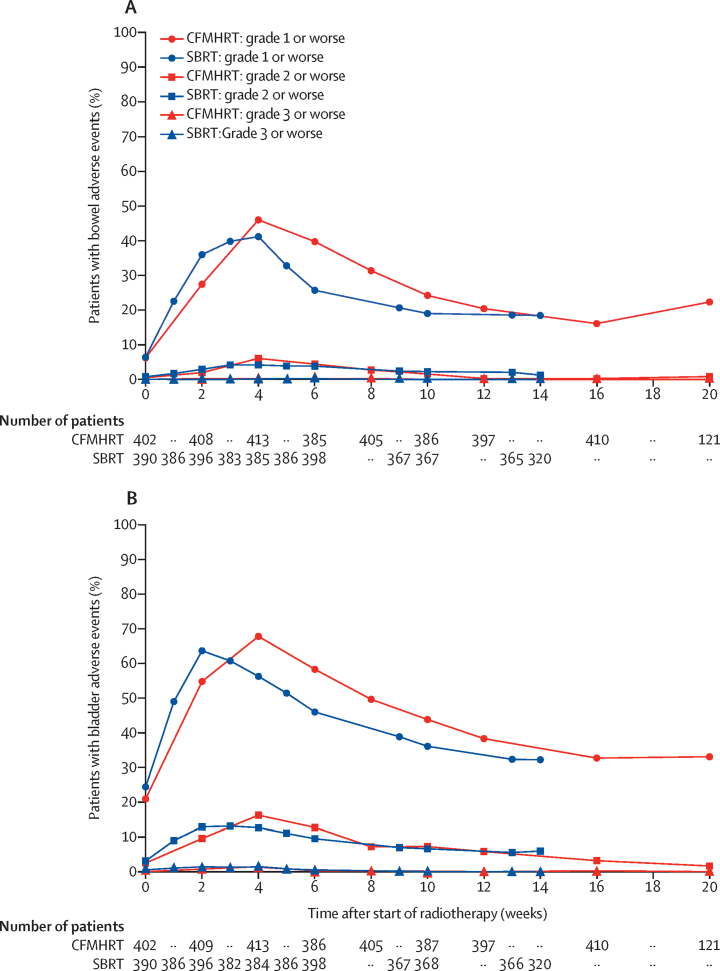
Acute Radiation Therapy Oncology Group toxicity for gastrointestinal (A) and genitourinary (B) systems As each group allowed two different treatment durations (CFMHRT 78 Gy in 39 fractions and 62 Gy in 20 fractions; SBRT 36·25 Gy in five fractions over 1 or 2 weeks) it was necessary to interpolate data where assessments did not overlap. Raw data are presented in the appendix (p 17), with all four schedules shown separately. Numbers at risk for each arm are asynchronous because they are shown only at data collection timepoints (which are non-simultaneous relative to the start of radiotherapy). Week 0 is the baseline toxicity score taken before start of radiotherapy. CFMHRT=conventionally fractionated or moderately hypofractionated radiotherapy. SBRT=stereotactic body radiotherapy.

We recorded RTOG acute toxicity over time for gastrointestinal and genitourinary toxic effects and observed a similar time course of toxicity peak and recovery between the groups (figure 2). Graphical representation of the four different durations of treatment separately (stereotactic body radiotherapy 1 week and 2 weeks and conventionally fractionated or moderately hypofractionated radiotherapy 4 weeks and 7·8 weeks) is shown in the appendix (p 17). The RTOG baseline, worst, worst (exceeding baseline), and week-12 after radiotherapy gastrointestinal and genitourinary toxic effects are summarised in the appendix (pp 15–16).

**Figure 3:**
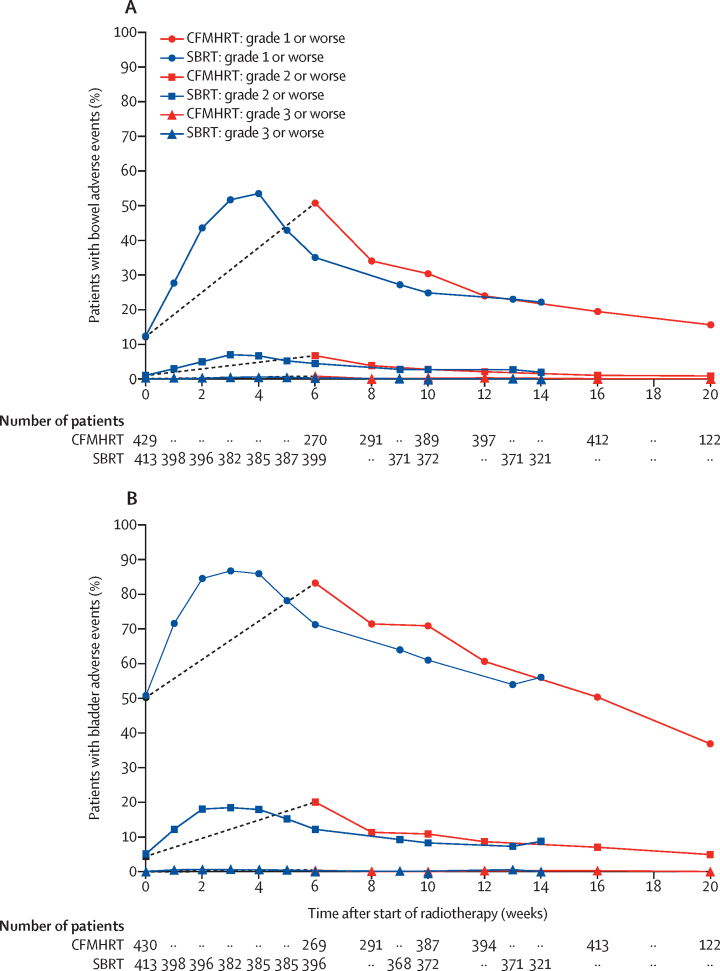
Acute CTCAE toxicity for gastrointestinal (A) and genitourinary systems As each group allowed two different treatment durations (CFMHRT 78 Gy in 39 fractions and 62 Gy in 20 fractions; SBRT 36·25 Gy in five fractions over 1 or 2 weeks) it was necessary to interpolate data. Raw data are presented in the appendix (p 21), with all four schedules presented separately. Numbers at risk for each arm are asynchronous because they are shown only at data collection timepoints (which are non-simultaneous relative to the start of radiotherapy). The initial points for CFMHRT are connected by grey dashed lines to emphasise that there were no CTCAE assessments during radiotherapy delivery. Week 0 is the baseline toxicity score taken before start of radiotherapy. CTCAE=Common Terminology Criteria for Adverse Events. CFMHRT=conventionally fractionated or moderately hypofractionated radiotherapy. SBRT=stereotactic body radiotherapy.

A summary table of all common and serious CTCAE adverse events is provided in the appendix (pp 18–19). 17 serious adverse events were reported (five in the conventionally fractionated or moderately hypofractionated radiotherapy group and 12 in the stereotactic body radiotherapy group) up to 12 weeks after radiotherapy, of which 15 (five in the conventionally fractionated or moderately hypofractionated radiotherapy and ten in the stereotactic body radiotherapy group) were related to treatment (appendix p 20). We recorded CTCAE acute toxicity over time for composite gastrointestinal and genitourinary toxic effects, and observed a similar time course of toxicity peak and recovery between stereotactic body radiotherapy and conventionally fractionated or moderately hypofractionated radiotherapy (figure 3). Graphical representation of the four different durations of treatment separately (stereotactic body radiotherapy 1 week and 2 weeks and conventionally fractionated or moderately hypofractionated radiotherapy 4 weeks and 7·8 weeks) is shown in the appendix (p 21). Data for composite gastrointestinal and genitourinary toxic effects, at baseline, worst, worst (exceeding baseline), and week 12 after radiotherapy are summarised in the appendix (pp 22–23), with the results of hypothesis testing. Stereotactic body radiotherapy was statistically significantly worse compared with the conventionally fractionated or moderately hypofractionated radiotherapy for two of the CTCAE secondary endpoints analysed—worst CTCAE grade 2 or more severe gastrointestinal toxic effects (36 [8%] of 430 patients *vs* 65 [16%] of 415 patients; difference 7·3 percentage points, 95% CI 2·9–11·7; p=0·0011), corroborated by worst CTCAE grade 2 or more severe gastrointestinal toxic effects exceeding baseline (34 [8%] of 427 patients *vs* 63 [15%] of 413 patients; difference 7·3 percentage points, 95% CI 3·0–11·6; p=0·00095; appendix p 22). Diarrhoea grade 2 and worst proctitis grade 2 occurred more frequently in the stereotactic body radiotherapy group. We found no significant difference in worst CTCAE grade 2 or more severe gastrointestinal toxic effects by week 12. We observed no other significant differences in CTCAE gastrointestinal secondary endpoints for conventionally fractionated or moderately hypofractionated radiotherapy compared with stereotactic body radiotherapy (appendix p 22), including worst CTCAE gastrointestinal grade 3 or more severe toxic effects (three [1%] of 430 patients in the conventionally fractionated or moderately hypofractionated radiotherapy *vs* three [1%] of 415 patients in the stereotactic body radiotherapy group). We observed no significant differences in CTCAE genitourinary secondary endpoints between the conventionally fractionated or moderately hypofractionated radiotherapy and stereotactic body radiotherapy groups (appendix p 23), including worst CTCAE genitourinary grade 3 or more severe toxic effects (three [1%] of 430 patients *vs* seven [2%] of 415 patients). Further tables broken down into individual CTCAE toxicity items, separately for gastrointestinal and genitourinary systems, are presented in the appendix (pp 24–39) and show baseline CTCAE toxicity, worst acute CTCAE toxicity, worst (exceeding baseline) acute CTCAE toxicity, and week 12 CTCAE toxicity.

**Figure 4:**
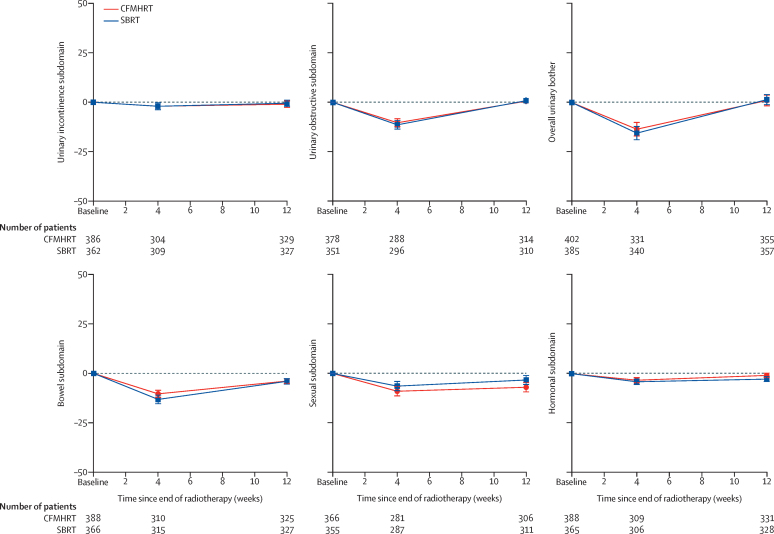
Changes from baseline in expanded prostate cancer index composite (26 question) subdomains Urinary bother is graphed separately, as it does not form part of the urinary incontinence or obstructive subdomain scores. Error bars show 95% CIs for estimates of mean subdomain scores. The time period between baseline scoring and week 4 after radiotherapy follow-up is variable, since the total time of radiotherapy delivery varied (SBRT in 1 or 2 weeks; CFMHRT in 4 or 7·8 weeks). Week 0 is the baseline score taken before start of radiotherapy. Scores are change from baseline, with 0 representing no change. CFMHRT=conventionally fractionated or moderately hypofractionated radiotherapy. SBRT=stereotactic body radiotherapy.

EPIC-26 mean changes in subdomain scores over time were similar, both for change from baseline (figure 4) and absolute scores (appendix p 40). Comparison over each of the five EPIC-26 subdomains and overall urinary bother for scores at baseline, worst, worst minus baseline, and week 12 after radiotherapy showed no significant differences between the trial groups (appendix p 41). We observed no significant difference between the study groups in the proportion of patients with a clinically significant reduction from baseline for any EPIC-26 subdomain score area, neither assessed at any time (appendix p 42) nor at week-12 only (appendix p 43).

IPSS subscores, total score, and quality of life over time were similar between the study groups, both for change from baseline (appendix p 44) and absolute scores (appendix p 45). We observed no significant differences between treatment groups for median scores of worst IPSS total, week-12 IPSS total, worst IPSS quality of life, or week-12 IPSS quality of life (appendix p 46). IPSS severity categories (none, mild, moderate, or severe) over time were similar between the treatment groups (appendix p 47), with no significant differences in IPSS total score categories at baseline, worst, and week-12 after radiotherapy (appendix p 48).

For IIEF-5, we observed no significant differences between conventionally fractionated or moderately hypofractionated radiotherapy and stereotactic body radiotherapy at baseline or at week 12 after radiotherapy (appendix p 49). Vaizey score changes were similar between treatment groups for both change from baseline and absolute scores (appendix pp 50–51). We observed no significant differences between treatment groups for Vaizey scores at baseline, worst, worst change from baseline, and week 12 after radiotherapy (appendix p 52).

For the stereotactic body radiotherapy group, worst RTOG gastrointestinal grade 2 or more severe (without reference to baseline) toxic effects for non-CyberKnife (27 [11%] of 245 patients) versus CyberKnife (16 [9%] of 170 patients) delivery was not different (difference −1·6 percentage points, 95% CI −7·5 to 4·3; p=0·597), consistent with observations over time (appendix p 53). For patients in the stereotactic body radiotherapy group, worst RTOG grade 2 or more severe genitourinary (without reference to baseline) toxic effects for non-CyberKnife (75 [31%] of 245 patients) versus CyberKnife (21 [12%] of 170 patients) delivery were significantly different (difference −18·3 percentage points, 95% CI −10·7 to −25·9; p<0·0001), consistent with observations over time (appendix p 54). Given the non-randomised nature of comparison of non-CyberKnife with CyberKnife delivery, we examined the conventionally fractionated or moderately hypofractionated radiotherapy toxicity in non-CyberKnife centres compared with CyberKnife centres. For patients in the conventionally fractionated or moderately hypofractionated radiotherapy group, worst RTOG gastrointestinal grade 2 or more severe (without reference to baseline) toxic effects in non-CyberKnife-using centres (25 [10%] of 252 patients) versus CyberKnife centres (28 [16%] of 180 patients) were not different (difference 5·6 percentage points, 95% CI −0·8 to 12·1; p=0·078), consistent with grade 2 and grade 3 observations over time (appendix p 55). For patients in the conventionally fractionated or moderately hypofractionated radiotherapy group, worst RTOG grade 2 or more severe genitourinary (without reference to baseline) toxic effects in non-CyberKnife-using centres (73 [29%] of 252 patients) versus CyberKnife-using centres (45 [25%] of 180 patients) were not significantly different (difference −4·0 percentage points, 95% CI −12·4 to 4·5; p=0·361), contrary to possible graphical interpretation over time (appendix p 56).

## Discussion

This pre-planned analysis of acute toxicity in the PACE-B trial, occurring up to 12 weeks after radiotherapy delivery completion, does not suggest that patients have greater acute RTOG toxic effects with stereotactic body radiotherapy compared with conventionally fractionated or moderately hypofractionated radiotherapy. Of the secondary endpoints examined, only worst CTCAE grade 2 or more severe composite toxic effects (both with and without reference to baseline) showed significantly higher proportions of patients with toxic effects when treated with stereotactic body radiotherapy compared with conventionally fractionated or moderately hypofractionated radiotherapy. Differences in CTCAE toxicity were resolved by week 12 after completion of radiotherapy. Patient-reported outcomes were similar between the trial groups. Overall, our results do not provide consistent evidence of higher acute toxicity with stereotactic body radiotherapy compared with conventionally fractionated or moderately hypofractionated radiotherapy.

It is notable that the control group in our trial (conventionally fractionated or moderately hypofractionated radiotherapy) had lower acute toxicity than in the preceding CHHiP trial,^11^ with toxicity more comparable to the PROFIT trial (appendix p 57).^14^ Although image-guided radiotherapy was mandatory in both the PACE and PROFIT^14^ trials, it was only used in 30% of CHHiP participants, which could have caused this difference. PACE also used smaller margins and benefitted from use of highly conformal techniques, such as volumetric modulated arc therapy. The CHHiP trial used androgen deprivation therapy for most patients, which was not permitted in PACE or PROFIT; however, androgen deprivation therapy is not known to alter acute toxicity. Both PROFIT and CHHiP assessed acute RTOG weekly during radiotherapy versus two-weekly assessment in PACE. Conceivably, the cumulative proportion of higher worst RTOG grade 2 or more severe events in CHHiP and PROFIT versus PACE-B might result from recall selection bias due to more frequent sampling in PROFIT and CHHiP.

The most similar phase 3 randomised controlled trial to PACE-B is the Scandinavian HYPO-RT-PC trial, which randomly assigned (1:1) intermediate-risk and high-risk patients with prostate cancer to 78 Gy in 39 fractions over 7·8 weeks or 42·7 Gy in seven fractions over 2·5 weeks, without androgen deprivation therapy.^19^ Important differences between PACE-B and HYPO-RT-PC are as follows: HYPO-RT-PC recruited 11% high-risk patients and 89% intermediate-risk patients (*vs* 8% low-risk patients and 92% intermediate-risk patients in PACE-B), treated a CTV of prostate only, and mostly (80%) used three-dimensional (3D) conformal radiotherapy. Image-guided radiotherapy (fiducial markers or guidance catheter) and planning MRI were used for all patients in HYPO-RT-PC. The control groups differ between HYPO-RT-PC (all 78 Gy in 39 fractions) and PACE-B (70% receiving 62 Gy in 20 fractions). This difference is important given the higher acute gastrointestinal toxicity observed for moderate hypofractionation in the CHHiP trial.^11^ HYPO-RT-PC made only a single end-of-treatment toxicity assessment during the acute toxicity window, and reported significantly higher RTOG genitourinary and patient-reported outcome acute toxic effects with ultra-hypofractionation. Comparison of RTOG toxicity for PACE-B with HYPO-RT-PC (estimates approximated from graphs in paper^19^) produces similar results, although reported grade 3 to grade 4 toxicity for HYPO-RT-PC is higher than most reports of ultra-hypofractionation (appendix p 58). Although measured on different patient-reported outcome scales to HYPO-RT-PC, our results do not suggest a difference in patient-reported outcome acute side-effects.

We identified no up to date systematic literature review of acute toxicity in this setting. Therefore, we prospectively collated acute toxicity data from smaller studies of stereotactic body radiotherapy in low-risk and intermediate-risk prostate cancer (appendix p 58). The PACE-B outcomes appear to be broadly in line with results anticipated from earlier phase work. For example, a multicentre phase 2 study of 309 men^25^ recorded cumulative acute toxicity of CTCAE gastrointestinal grade 2 or worse of 12% and CTCAE genitourinary grade 2 or worse of 26%, similar to the 15·7% and 30·8%, respectively, for patients in the stereotactic body radiotherapy group in PACE-B.

Strengths of these data relate predominantly to trial design. This is a large phase 3 randomised, controlled trial, and represents, to our knowledge, the first published phase 3 acute toxicity data on five-fraction stereotactic body radiotherapy compared with standard fractionation. PACE-B reflects real world prostate radiotherapy practice, with multiple centres recruiting in the UK, Canada, and Ireland. This study incorporates modern planning practice, with no patients receiving 3D conformal radiotherapy. The protocol amendment relating to treatment in the control group strengthened the trial by allowing most patients in that group to receive moderate hypofractionation at 62 Gy in 20 fractions, close to the 60 Gy in 20 fractions regimen shown to be effective in CHHiP^11^ and PROFIT.^14^ The PACE-B acute toxicity sampling frequency exceeded HYPO-RT-PC (assessed only at end of radiotherapy and 6 months). Combined with the high proportions of assessment forms returned, this is a major strength given the dynamic nature of acute toxicity.

Limitations arise from the external applicability of the patients recruited to PACE-B. These results cannot necessarily be extrapolated to higher-risk patients, nor alternative treatment techniques. Randomised data regarding toxicity after stereotactic body radiotherapy, with concurrent androgen deprivation therapy and a larger target volume, will be acquired by the PACE-C trial. This trial cohort will randomly assign unfavourable intermediate-risk and lower high-risk patients to either stereotactic body radiotherapy or moderately hypofractionated radiotherapy. The absence of treatment blinding is always a limitation for subjective endpoints, such as toxicity. Although blinding has been achieved in previous radiotherapy trials,^26^, ^27^ it is not feasible for most studies. We also note the higher fiducial marker use for image-guided radiotherapy in patients undergoing stereotactic body radiotherapy compared with conventionally fractionated or moderately hypofractionated radiotherapy in PACE-B. Mandatory fiducials would have prevented some centres participating, slowing trial recruitment. Furthermore, the multiple radiotherapy schedule durations meant that some undesirable interpolation was needed to present two arm graphs (RTOG and CTCAE). This fact also means that the follow up of 12 weeks after radiotherapy refers to quite different period of time for someone receiving 1 week of stereotactic body radiotherapy (ie, 13 weeks from the start of radiotherapy) versus 7·8 weeks of conventional fractionation (ie, 19·8 weeks after commencing treatment). Future trials should consider a follow-up schedule fixed by radiotherapy start date rather than end date.

Stereotactic body radiotherapy is already the standard of care in some centres and is an option for men with low and favourable intermediate-risk prostate cancer in the NCCN guidelines.^28^ The HYPO-RT-PC trial suggested similar oncological outcomes with ultra-hypofractionation.^19^ This result was attenuated by increased acute toxicity in the study, notably higher grade 3 or worse toxic effects than other reports of stereotactic body radiotherapy, which might potentially be driven by the 3D conformal radiotherapy technique predominantly used in the HYPO-RT-PC study. Other earlier phase studies, most of which used the same 36·25 Gy dose as PACE (appendix p 58), suggest good oncological outcomes and low late toxicity with stereotactic body radiotherapy, but the mature results of PACE-B are required before definite oncological outcome statements can be made.

The method of stereotactic body radiotherapy delivery—for example, CyberKnife versus non-CyberKnife—might influence acute toxicity, a prespecified area of interest after the introduction of conventional linear accelerator stereotactic body radiotherapy. There are many reasons why there might be a systematic difference between CyberKnife and non-CyberKnife stereotactic body radiotherapy outcomes, including variations in dosimetry, image guidance, and treatment times (typically 45 min for CyberKnife and <5 min for conventional linear accelerators). Our post-hoc analysis of the same primary endpoint RTOG metrics shows similar grade 2 or worse gastrointestinal toxic effects, but less grade 2 or worse genitourinary toxic effects with CyberKnife. We compared conventionally fractionated or moderately hypofractionated radiotherapy toxic effects between centres using CyberKnife versus those not using CyberKnife and found no significant difference for either worst RTOG grade 2 or more severe gastrointestinal or genitourinary toxic effects. We caution that this result is hypothesis-generating and intend to explore further in multivariate analyses once digital imaging and communications in medicine data have been centralised for all patients.

To our knowledge, we present the first published prospective phase 3 acute toxicity results for random assignment of patients between five-fraction stereotactic body radiotherapy and either conventional or moderately hypofractionated radiotherapy. Our results do not suggest that patients have greater acute RTOG toxic effects with stereotactic body radiotherapy compared with conventionally fractionated or moderately hypofractionated radiotherapy. The absence of increased toxicity in the stereotactic body radiotherapy group is reassuring given the higher acute toxicity suggested in the only previously published phase 3 ultra-hypofractionation trial,^19^ especially given the more abbreviated (five-fraction) investigational radiotherapy protocol used in PACE-B. Results regarding late toxicity and biochemical control from PACE-B will be reported in the next 3-4 years.

## Data sharing

The Institute of Cancer Research Clinical Trials and Statistics Unit (ICR-CTSU) supports the wider dissemination of information from the research it conducts and increased cooperation between investigators. Trial data is collected, managed, stored, shared, and archived according to ICR-CTSU Standard Operating Procedures to ensure the enduring quality, integrity, and utility of the data. Formal requests for data sharing are considered in line with ICR-CTSU procedures with due regard given to funder and sponsor guidelines. Requests are via a standard proforma describing the nature of the proposed research and extent of data requirements. Data recipients are required to enter a formal data sharing agreement, which describes the conditions for release and requirements for data transfer, storage, archiving, publication, and intellectual property. Requests are reviewed by the trial management group (TMG) in terms of scientific merit and ethical considerations including patient consent. Data sharing is undertaken if proposed projects have a sound scientific or patient benefit rationale as agreed by the TMG and approved by the independent data monitoring and steering committee as required. Restrictions relating to patient confidentiality and consent will be limited by aggregating and anonymising identifiable patient data. Additionally, all indirect identifiers that might lead to deductive disclosures will be removed in line with Cancer Research UK Data Sharing Guidelines.
