# Supplementary material for: Intensity-modulated fractionated radiotherapy versus stereotactic body radiotherapy for prostate cancer (PACE-B): acute toxicity findings from an international, randomised, open-label, phase 3, non-inferiority trial
Source: Lancet Oncol. 2019 Nov;20(11):1531–43. doi: 10.1016/S1470-2045(19)30569-8 (PMC6838670; doi:10.1016/S1470-2045(19)30569-8)
Supplement: Supplementary appendix [file mmc1.pdf]

THE LANCET
Oncology

Supplementary appendix

This appendix formed part of the original submission and has been peer reviewed. We post it as supplied by the authors.

Supplement to: Brand DH, Tree AC, Ostler P, et al. Intensity-modulated fractionated radiotherapy versus stereotactic body radiotherapy for prostate cancer (PACE-B): acute toxicity findings from an international, randomised, open-label, phase 3, non-inferiority trial. *Lancet Oncol* 2019; published online Sept 17. http://dx.doi.org/10.1016/S1470-2045(19)30569-8.

Supplementary Appendices

Contents

Supplementary Appendix 1. Trial Steering Committee Members & Independent Data Monitoring Committee During the Course of PACE B Trial........3

Supplementary Appendix 2. Dose Constraints Used Over the Course of the PACE Trial........4

Supplementary Table 1. Original CFMHRT Dose Constraints........4

Supplementary Table 2. Original SBRT Dose Constraints........4

Supplementary Appendix 3. Details of Two Arm Graph Construction for RTOG and CTCAE Toxicity....6

Supplementary Appendix 4. List of Recruiting Centres and Investigators........7

Supplementary Table 3. List of All Patients Prescribed Non-Protocol Regimen or Receiving Dose Reduction Due to Treatment Toxicity........8

Supplementary Table 4. Treatment Characteristics by Treatment Arm........9

Supplementary Appendix 5. CTV to PTV Margins Used By Treatment Arm........11

Supplementary Table 5. RTOG Assessment Completion Rates........12

Supplementary Table 6. CTCAE Assessment Completion Rates........12

Supplementary Table 7. EPIC-26 Assessment Completion Rates........13

Supplementary Table 8. IPSS Assessment Completion Rates........13

Supplementary Table 9. IIEF-5 Assessment Completion Rates........14

Supplementary Table 10. Vaizey Assessment Completion Rates........14

Supplementary Table 11. Baseline, Worst and Worst Exceeding Baseline for Acute Gastrointestinal RTOG Toxicity........15

Supplementary Table 12. Baseline, Worst and Worst Exceeding Baseline for Acute Genitourinary RTOG Toxicity........16

Supplementary Figure 1. Acute RTOG Toxicity By Treatment Duration: Gastrointestinal and Genitourinary........17

Supplementary Table 13. Standardised Summary Table of CTCAE Adverse Events........18

Supplementary Table 14. Summary of All Serious Adverse Events and Serious Adverse Reactions Occurring During Acute Toxicity Period........20

Supplementary Figure 2. Acute CTCAE Toxicity By Treatment Duration: Gastrointestinal and Genitourinary........21

Supplementary Table 15. Summary of CTCAE GI Composite Toxicity........22

Supplementary Table 16. Summary of CTCAE GU Composite Toxicity........23

Supplementary Table 17. Baseline CTCAE Gastrointestinal (GI) Toxicity........24

Supplementary Table 18. Baseline CTCAE Genitourinary (GU) Toxicity........26

Supplementary Table 19. Worst Acute CTCAE Gastrointestinal (GI) Toxicity........28

Supplementary Table 20. Worst Acute CTCAE Genitourinary (GU) Toxicity 30
Supplementary Table 21. Worst (Exceeding Baseline) Acute CTCAE Gastrointestinal (GI) Toxicity..... 32
Supplementary Table 22. Worst (Exceeding Baseline) Acute CTCAE Genitourinary (GU) Toxicity 34
Supplementary Table 23. Week 12 Acute CTCAE Gastrointestinal (GI) Toxicity 36
Supplementary Table 24. Week 12 CTCAE Genitourinary (GU) Toxicity 38
Supplementary Figure 3. Mean average EPIC-26 subdomain scores in the acute toxicity setting, separated by delivered radiotherapy technique 40
Supplementary Table 25. Comparison of Median Scores for EPIC-26 Subdomains 41
Supplementary Table 26. EPIC-26 Score Reductions at Any Timepoint (up to 12 weeks post radiotherapy) Exceeding Minimal Clinically Important Differences 42
Supplementary Table 27. EPIC-26 Score Reductions at 12 Weeks post Radiotherapy Exceeding Minimal Clinically Important Differences 43
Supplementary Figure 4. Change from Baseline IPSS Scores... 44
Supplementary Figure 5. Average IPSS Subscores, Total & Quality of Life.. 45
Supplementary Table 28. Comparison of Median IPSS Total Scores and Quality of Life Scores 46
Supplementary Figure 6. IPSS Severity Categories Over Time.. 47
Supplementary Table 29. Comparison of IPSS Total Score Categories... 48
Supplementary Table 30. Comparison of Median IIEF-5 Scores... 49
Supplementary Figure 7. Change from Baseline Vaizey Total Scores... 50
Supplementary Figure 8. Vaizey Total Scores Between Baseline and Week 12 Post Radiotherapy 51
Supplementary Table 31. Comparison of Median Vaizey Total Scores 52
Supplementary Figure 9. RTOG Gastrointestinal Acute Toxicity (For SBRT Patients Only) By Delivery Platform 53
Supplementary Figure 10. RTOG Genitourinary Acute Toxicity (For SBRT Patients Only) By Delivery Platform 54
Supplementary Figure 11. RTOG Gastrointestinal Acute Toxicity (For CFMHRT Patients Only) By Treating Centre CyberKnife Status.. 55
Supplementary Figure 12. RTOG Genitourinary Acute Toxicity (For CFMHRT Patients Only) By Treating Centre CyberKnife Status.. 56
Supplementary Table 32. PACE RTOG Toxicity with Reference to Comparable Hypofractionation Trials.. 57
Supplementary Table 33. Reported Toxicity After SBRT to the Prostate in Low-Intermediate Risk Patients 58
Bibliography for Supplementary Table 33 60

<u>Supplementary Appendix 1. Trial Steering Committee Members & Independent Data Monitoring Committee During the Course of PACE B Trial</u>

Our thanks to those members of the Trial Steering Committee and Independent Data Monitoring Committee

Trial Steering Committee Members:
Anthony Zietman
Ann Henry
John Norrie
Alberto Bossi
Alberto Briganti
Gert De Meerler
Piet Ost

Independent Data Monitoring Committee
Raj Persad
Søren Bentzen
Joe O'Sullivan

Supplementary Appendix 2. Dose Constraints Used Over the Course of the PACE Trial

Over the course of trial recruitment, several changes were made to the normal tissue dose constraints applied during radiotherapy planning. The final constraints used are detailed in the final protocol (version 9), (**Appendix p97-98**). Below are the original dose constraints used in version 1 of the protocol and dates of changes made. From Protocol version 7, (24/03/2016), patients could be treated with 62Gy in 20 fractions. The dose constraints for these patients were proportionally scaled to those for 74 Gy in 37 fractions listed below. E.g. V74 = V62, V70 = V57 etc.

Protocol Version 3 (19/07/2012). The first clinically used protocol version.

First patient randomised 07/08/12. Total 118/847 patients randomised with these constraints

Supplementary Table 1. Original CFMHRT Dose Constraints

OAR	Dose Constraint (2 Gy per fraction)	Max Vol (% or cc)	Notes
Rectum	V30	80%	Recommended
	V40	70%	Recommended
	V50	60%	
	V60	50%	
	V65	30%	
	V70	25%	
	V74	15%	Mandatory
	V74	5%	Recommended
Bladder	V50	50%	
	V60	25%	
	V74	5%	
Femoral Heads	V50	50%	
Bowel	V50	17cc	

Supplementary Table 2. Original SBRT Dose Constraints

OAR	Dose constraint	Max Vol (% or cc)
Rectum	V18.1	50%
	V29	20%
	V36	1 cc
Bladder	V18.1	40%
	V37	10 cc
Prostatic urethra (if visualized)	V44	20%
Neurovascular bundle (if seen)	V38	50%
Femoral head	V14.5	5%
Penile Bulb	V29.5	50%
Testicular	Blocking structure	
Bowel	V18.1	5 cc
	V30	1 cc

Version 5 (05/08/2014)
Total 58/847 patients randomised with these constraints

SBRT

- Bladder. V37<5cc optimal constraint added
- Prostatic urethra. V44<20% (v3) changed to V42<50% (optional)

Version 6 (22/06/2015)
Total 67/847 patients randomised with these constraints

SBRT

- REMOVED OAR: Neurovascular bundle constraint

Version 7 (24/03/2016) onwards (including version 8 and 9)
Total 604/847 patients randomised with these constraints

CFMHRT

- Rectum. V30<80% re-termed "optimal"
- Rectum. V40<70% changed to V40<65% (optimal)
- Rectum. Added V50<50% (optimal)
- Rectum. Added V60<35% (optimal)
- Rectum. Added V70<15% (optimal)
- Rectum. Changed V75<15% mandatory to V75<5% mandatory
- Rectum. Changed V75<5% recommended to V75<3% optimal
- Bladder. Added V74<5% (optimal)
- NEW OAR: Penile bulb V50<50% (optimal)
- NEW OAR: Penile bulb V60<10% (optimal)

Supplementary Appendix 3. Details of Two Arm Graph Construction for RTOG and CTCAE Toxicity

There is difficulty in producing graphs showing toxicity over time for two arms (CFMHRT and SBRT), with the x-axis beginning at the start of radiotherapy (represented by the baseline data). This is caused by each arm having two schedules of different durations:

- CFMHRT has:
  - 78 Gy in 39 fractions over 7.8 weeks
  - 62 Gy in 20 fractions over 4 weeks
- SBRT has
  - 36.25 Gy in 5 fractions over 1 week
  - 36.25 Gy in 5 fractions over 2 weeks

Therefore, the follow-up assessments do not necessarily fall at the same time for each schedule. For example, week 2 follow-up post RT for 1-week SBRT occurs at 3 weeks from start of RT, whereas it will occur 4 weeks from start of RT for 2-week SBRT patients.

For a given grade (e.g. G1+), each patient is scored as a 1 (toxicity of that grade or more) or 0 (toxicity less than that grade). We wish to show at each timepoint the proportion of patients with grade 1+, grade 2+ and grade 3+ toxicity. For example, a patient with Grade 2 toxicity at a timepoint would be grade 1+ = 1 (yes), grade 2+ = 1 (yes), grade 3+ = 0 (no)

To obtain interpolated score for (e.g.) Grade 1+ at week 6 from start of RT, for a given patient not assessed at that timepoint:

  - Take G1+ toxicity status (0/1) at week 4.
  - Add G1+ toxicity status (0/1) at week 8.
  - Multiply by 0.5 (since the timepoint of interest is halfway between the known measurements)

The final multiplier could be altered if a different week of interest required interpolated data. In the above example, if week 5 interpolated data were required, then a final multiplier of 0.25 would be applied.

This of course assumes that patients' probability of having a toxicity or not changes in a linear fashion between timepoints.

The final point of each line contains only data from the longer of the two schedules to avoid extrapolation of data for the shorter schedule.

<u>Supplementary Appendix 4. List of Recruiting Centres and Investigators</u>

Royal Marsden Hospital, London, n=172, Dr N van As
Mount Vernon Hospital, Middlesex, n=114, Dr P Ostler
James Cook University Hospital, Middlesbrough, n=111, Dr H Van der Voet
Odette Cancer Centre, Toronto, n=83, Dr W Chu
Churchill Hospital, Oxford, n=41, Dr P Camilleri
Queen Elizabeth Hospital, Birmingham, n=36, Dr D Ford
Leicester Royal Infirmary, Leicester, n=34, Dr K Kancherla
Freeman Hospital, Newcastle, n=30, Dr J Frew
UHCW NHS Trust, Coventry & Warwickshire, n=30, Dr A Chan
Clatterbridge Cancer Centre, n=25, Dr S Tolan
Juravinski Cancer Centre, Ontario, n=24, Dr I Dayes
Belfast City Hospital, n=21, Dr S Jain
St Bartholomew's Hospital, London, n=17, Dr P Wells
Hôspital Charles-LeMoyne, Quebec, n=15, Dr T Lymberiou
Cambridge University Hospital, n=13, Dr A Martin
Nottingham City Hospital, n=11, Dr D Saunders
Royal Free Hospital, London, n=11, Dr M Vilarino-Varela
Hôspital Maisonneuve-Rosemont, Quebec, n=11, Dr P Vavassis
Walker Family Cancer Centre, Ontario, n=9, Dr T Tsakiridis
Hinchingbrooke Healthcare NHST, Cambridge, n=7, Dr A Martin
Northeast Cancer Centre, Ontario, n=7, Dr R Carlson
London Health Sciences Centre, Ottawa, Ontario, n=7, Dr G Rodrigues
Velindre Cancer Centre, Cardiff, n=6, Dr J Tanguay
Sunderland Royal Hospital, n=5, Dr S Iqbal
Charing Cross Hospital, London, n=5, Dr M Winkler
The Ottawa Hospital Cancer Centre, Ontario, n=5, Dr S Morgan
Beacon Hospital, Dublin, n=4, Dr A Mihai
Lakeridge Health, Oshawa, Ontario, n=4, Dr A Li
Weston Park Hospital, Sheffield, n=4, Dr O Din
Lincoln County Hospital, n=3, Dr M Panades
Norfolk & Norwich University Hospital, n=3, Dr R Wade
West Suffolk Hospital, n=2, Dr Y Rimmer
Beaumont Hospital (SLRON), Dublin n=2, Dr J Armstrong
Pilgrim Hospital, Lincolnshire, n=1, Dr M Panades
Glan Clwyd, North Wales, n=1, Dr N Oommen.

Supplementary Table 3. List of All Patients Prescribed Non-Protocol Regimen or Receiving Dose Reduction Due to Treatment Toxicity

Patient	Randomised	Per Protocol	Delivered Regimen	Radiotherapy Toxicity Related?	Reason that Non-Protocol Regimen Delivered
1	CFMHRT	CFMHRT	60 Gy in 20 F	No	Patient wanted 4-week regimen but was consented before 4 weekly regimen amendment occurred.
2	CFMHRT	CFMHRT	64 Gy in 32 F	No	Radiotherapy planning issue (small bowel proximity to prostate). Lower dose regimen prescribed.
3	CFMHRT	CFMHRT	74 Gy in 37 F	No	Pre-radiotherapy a protocol deviation to give ADT occurred, so given standard off-trial dose regimen for concurrent ADT usage
4	CFMHRT	CFMHRT	76 Gy in 38 F	No	Radiotherapy planning issue (Dose constraints not met so lower dose used)
5	SBRT	SBRT	14.5 Gy in 2 F then 46 Gy in 23 F	**Yes**	G3 urinary toxicity caused treatment interruption after 2 fractions SBRT. Completed treatment with conventional fractionation
6	SBRT	SBRT	21.75 in 3 F	No	On-treat dosimetry issue. Concerns that normal tissue dose constraints being violated. Decided not to deliver last 2 fractions
7	SBRT	CFMHRT	60 Gy in 20 F	No	Radiotherapy planning issue (bowel volume). Standard-of-care treatment preferred.
8	SBRT	CFMHRT	60 Gy in 20 F	No	Radiotherapy planning issue (bowel proximity to prostate). Standard-of-care treatment preferred.
9	SBRT	CFMHRT	60 Gy in 20 F	No	Radiotherapy planning issue (dosimetry at planning). Standard-of-care treatment preferred.
10	SBRT	CFMHRT	74 Gy in 37 F	No	Significant pre-existent urinary symptoms not recognised until planning CT. Thus had standard of care radiotherapy (with ADT).
11	SBRT	N/A	7.25Gy in 1 F then 55Gy in 20 F	No	On-treat issue. Patient moved during the delivery of first SBRT fraction. Decided to complete course with modified conventional regimen.

Supplementary Table 4. Treatment Characteristics by Treatment Arm

Treatment Characteristic	Per Protocol Treatment				Total	
	CFMHRT		SBRT			
	n	%	n	%	n	%
**Fiducial Markers Inserted?**						
No	187	43.3%	112	27.0%	299	35.3%
Yes	245	56.7%	303	73.0%	548	64.7%
**Number of Fiducial Markers**						
0	187	43.3%	112	27.0%	299	35.3%
2	4	0.9%	11	2.7%	15	1.8%
3	184	42.6%	94	22.7%	278	32.8%
4	51	11.8%	189	45.5%	240	28.3%
5+	1	0.2%	9	2.2%	10	1.2%
Unknown	5	1.2%	0	0.0%	5	0.6%
**Radiotherapy Delivery Method**						
Step and Shoot IMRT	106	24.5%	3	0.7%	109	12.9%
VMAT	322	74.5%	242	58.3%	564	66.6%
Tomotherapy	4	0.9%	0	0.0%	4	0.5%
CyberKnife	0	0.0%	170	41.0%	170	20.1%
**IGRT Method**						
Planar Film - With Fiducials	94	21.8%	10	2.4%	104	12.3%
Planar Intra-fractional Tracking	2	0.5%	170	41.0%	172	20.3%
CBCT - No Fiducials	185	42.8%	112	27.0%	297	35.1%
CBCT - With Fiducials	124	28.7%	116	28.0%	240	28.3%
CBCT & Planar Film Mix – No Fiducials	1	0.2%	0	0.0%	1	0.1%
CBCT & Planar Film Mix – With Fiducials	23	5.3%	4	1.0%	27	3.2%
CBCT & Planar Intra-Fractional Tracking	3	0.7%	3	0.7%	6	0.7%
**Overall Treatment Time**						
1 week	0	0.0%	86	20.7%	86	10.2%
2 weeks	0	0.0%	305	73.5%	305	36.0%
3 weeks	0	0.0%	18	4.3%	18	2.1%
4 weeks	136	31.5%	2	0.5%	138	16.3%
5 weeks	162	37.5%	4	1.0%	166	19.6%
6 weeks	4	0.9%	0	0.0%	4	0.5%
7 weeks	2	0.5%	0	0.0%	2	0.2%
8 weeks	61	14.1%	0	0.0%	61	7.2%
9 weeks	66	15.3%	0	0.0%	66	7.8%
10 weeks	1	0.2%	0	0.0%	1	0.1%

**Alpha Blockers Prescribed in Acute Window**						
No	308	71.3%	299	72.0%	607	71.7%
Yes	50	11.6%	39	9.4%	89	10.5%
Using at Randomisation	68	15.7%	67	16.1%	135	15.9%
Unknown	6	1.4%	10	2.4%	16	1.9%
**Anticholinergics Prescribed in Acute Window**						
No	406	94.0%	392	94.5%	798	94.2%
Yes	7	1.6%	8	1.9%	15	1.8%
Using at Randomisation	16	3.7%	10	2.4%	26	3.1%
Unknown	3	0.7%	5	1.2%	8	0.9%
**PDE5 Inhibitor Prescribed in Acute Window**						
No	402	93.1%	383	92.3%	785	92.7%
Yes	10	2.3%	8	1.9%	18	2.1%
Using at Randomisation	12	2.8%	6	1.4%	18	2.1%
Unknown	8	1.9%	18	4.3%	26	3.1%
**Totals**	432	100%	415	100%	847	100%

Supplementary Appendix 5. CTV to PTV Margins Used By Treatment Arm

The CTV to PTV margins varied by treatment arm. The protocol recommendations were as follows:

- CFMHRT non-posterior margins: 5-9mm
- CFMHRT posterior margin: 3-7mm
- SBRT non-posterior margins: 4-5mm
- SBRT posterior margin: 3-5mm

Actual margins used were as follows:

CFMHRT, non-posterior margins:

- <5 mm (n=5, 1·2%)
- 5-9 mm (n=406, 94·0%)
- 10 mm (n=5, 1·2%)
- Unknown (n=16, 3·7%)

CFMHRT posterior margins:

- <3 mm (n=9, 2·1%)
- 3-7 mm (n=407, 94·2%)
- Unknown (n=16, 3·7%)

SBRT non-posterior margins:

- <4 mm (n=40, 9·6%)
- 4-5 mm (n=366, 88·2%)
- >5 mm (n=6, 1·4%)
- Unknown (n=3, 0·7%)

SBRT posterior margins:

- <3 mm (n=53, 12·8%)
- 3-5 mm (n=357, 86·0%)
- >5 mm (n=2, 0·5%)
- Unknown (n=3, 0·7%).

Supplementary Table 5. RTOG Assessment Completion Rates

RTOG assessment counted as assessed if any useable toxicity data recorded.

RTOG Assessment	Per Protocol Treatment				Total	
	CFMHRT		SBRT			
	n	%	n	%	n	%
**RTOG Baseline**						
Assessed	402	93.1%	390	94.0%	792	93.5%
**RTOG RT Week 2 (CFMHRT Only)**						
Assessed	409	94.7%	N/A	N/A	409	94.7%
**RTOG RT Week 4 (CFMHRT Only)**						
Assessed	413	95.6%	N/A	N/A	413	95.6%
**RTOG RT Week 6 (CFMHRT >25 Fractions Only)**						
Assessed	116	89.9%	N/A	N/A	116	89.9%
**RTOG RT Week 8 (CFMHRT >35 Fractions Only)**						
Assessed	116	90.6%	N/A	N/A	116	90.6%
**RTOG End of Treatment (SBRT Only)**						
Assessed	N/A	N/A	400	96.4%	400	96.4%
**RTOG Post-RT Week 2**						
Returned	388	89.8%	389	93.7%	777	91.7%
**RTOG Post-RT Week 4**						
Returned	409	94.7%	403	97.1%	812	95.9%
**RTOG Post-RT Week 8**						
Returned	391	90.5%	372	89.6%	763	90.1%
**RTOG Post-RT Week 12**						
Returned	418	96.8%	402	96.9%	820	96.8%

Supplementary Table 6. CTCAE Assessment Completion Rates

CTCAE assessment counted as assessed if any useable toxicity data recorded.

CTCAE Assessment	Per Protocol Treatment				Total	
	CFMHRT		SBRT			
	n	%	n	%	n	%
**CTCAE Baseline**						
Assessed	430	99.5%	413	99.5%	843	99.5%
**CTCAE End of Treatment (SBRT Only)**						
Assessed	N/A	N/A	399	96.1%	399	96.1%
**CTCAE Post-RT Week 2**						
Assessed	389	90.0%	390	94.0%	779	92.0%
**CTCAE Post-RT Week 4**						
Assessed	410	94.9%	403	97.1%	813	96.0%
**CTCAE Post-RT Week 8**						
Assessed	393	91.0%	374	90.1%	767	90.6%
**CTCAE Post-RT Week 12**						
Assessed	420	97.2%	405	97.6%	825	97.4%

Supplementary Table 7. EPIC-26 Assessment Completion Rates

Assessment for EPIC-26 scored as assessed if any subdomain fully completed, or if overall urinary bother question completed.

EPIC-26 Assessment	Per Protocol Treatment				Total	
	CFMHRT		SBRT			
	n	%	n	%	n	%
**EPIC-26 Baseline**						
Assessed	405	93.8%	387	93.3%	792	93.5%
**EPIC-26 Post-RT Week 4**						
Assessed	354	81.9%	362	87.2%	716	84.5%
**EPIC-26 Post-RT Week 12**						
Assessed	380	88.0%	382	92.0%	762	90.0%

Supplementary Table 8. IPSS Assessment Completion Rates

IPSS assessment counted as assessed if IPSS total score calculable, or if quality of life question completed.

IPSS Assessment	Per Protocol Treatment				Total	
	CFMHRT		SBRT			
	n	%	n	%	n	%
**IPSS Baseline**						
Assessed	399	92.4%	384	92.5%	783	92.4%
**IPSS Post-RT Week 2**						
Assessed	364	84.3%	358	86.3%	722	85.2%
**IPSS Post-RT Week 4**						
Assessed	347	80.3%	351	84.6%	698	82.4%
**IPSS Post-RT Week 8**						
Assessed	354	81.9%	346	83.4%	700	82.6%
**IPSS Post-RT Week 12**						
Assessed	371	85.9%	371	89.4%	742	87.6%

Supplementary Table 9. IIEF-5 Assessment Completion Rates

IIEF-5 assessment counted as assessed if IIEF-5 total score calculable.

IPSS Assessment	Per Protocol Treatment				Total	
	CFMHRT		SBRT			
	n	%	n	%	n	%
**IIEF-5 Baseline**						
Assessed	322	74.5%	309	74.5%	631	74.5%
**IIEF-5 Post-RT Week 12**						
Assessed	280	64.8%	286	68.9%	566	66.8%

Supplementary Table 10. Vaizey Assessment Completion Rates

Vaizey assessment counted as assessed if Vaizey total score calculable.

Vaizey Assessment	Per Protocol Treatment				Total	
	CFMHRT		SBRT			
	n	%	n	%	n	%
**Vaizey Baseline**						
Assessed	373	86.3%	358	86.3%	731	86.3%
**Vaizey Post-RT Week 4**						
Assessed	267	61.8%	276	66.5%	543	64.1%
**Vaizey Post-RT Week 12**						
Assessed	349	80.8%	352	84.8%	701	82.8%

Supplementary Table 11. Baseline, Worst and Worst Exceeding Baseline for Acute Gastrointestinal RTOG Toxicity

n.b Percentages may differ by 0.1% in sums due to all cells being rounded to 1 decimal place

RTOG Gastrointestinal (GI) Toxicity	Per Protocol Treatment						Statistical Comparisons
	CFMHRT			SBRT			
	No.	%	Grade X+ %	No.	%	Grade X+ %	
**Baseline GI Grade**							
Grade 0	377	93.8%	**100.0%**	365	93.6%	**100.0%**	p=0·90 Mann-Whitney comparing grade frequencies
Grade 1	23	5.7%	**6.2%**	22	5.6%	**6.4%**	
Grade 2	2	0.5%	**0.5%**	3	0.8%	**0.8%**	
Grade 3	0	0.0%	**0.0%**	0	0.0%	**0.0%**	
Grade 4	0	0.0%	**0.0%**	0	0.0%	**0.0%**	
Missing	30	N/A	**N/A**	25	N/A	**N/A**	
							**Comparisons of Grade X+ %**
**Worst GI Grade**							
Grade 0	115	26.6%	**100.0%**	153	36.9%	**100.0%**	
Grade 1	264	61.1%	**73.4%**	219	52.8%	**63.1%**	
Grade 2	49	11.3%	**12.3%**	42	10.1%	**10.4%**	−1.9% difference 95% CI −6.2 to 2.4% p=0.38 (Chi-square)
Grade 3	4	0.9%	**0.9%**	1	0.2%	**0.2%**	−0.7% difference 95% CI −1.7 to 0.3% p=0.37 (Fisher's)
Grade 4	0	0.0%	**0.0%**	0	0.0%	**0.0%**	
**Worst GI Grade, Exceeding Baseline Grade**							
No Baseline Data	30	N/A	**N/A**	25	N/A	**N/A**	
Baseline Not Exceeded	123	30.6%	**100.0%**	160	41.0%	**100.0%**	
Grade 1	230	57.2%	**69.4%**	195	50.0%	**59.0%**	
Grade 2	45	11.2%	**12.2%**	34	8.7%	**9.0%**	−3.2% difference 95% CI −7.5 to 1.1% p=0.14 (Chi-square)
Grade 3	4	1.0%	**1.0%**	1	0.3%	**0.3%**	−0.7% difference 95% CI −1.8 to 0.4% p=0.37 (Fisher's)
Grade 4	0	0.0%	**0.0%**	0	0.0%	**0.0%**	
**Week 12 Post-RT GI Grade**							
Grade 0	350	83.7%	**100.0%**	330	82.1%	**100.0%**	
Grade 1	66	15.8%	**16.3%**	65	16.2%	**17.9%**	
Grade 2	2	0.5%	**0.5%**	7	1.7%	**1.7%**	1.3% difference 95% CI −0.2 to 2.7% p=0.10 (Fishers')
Grade 3	0	0.0%	**0.0%**	0	0.0%	**0.0%**	N/A
Grade 4	0	0.0%	**0.0%**	0	0.0%	**0.0%**	
Missing	14	N/A	**N/A**	13	N/A	**N/A**	
**Total**	**432**			**415**			

Supplementary Table 12. Baseline, Worst and Worst Exceeding Baseline for Acute Genitourinary RTOG Toxicity

RTOG Genitourinary (GU) Toxicity	Per Protocol Treatment						Statistical Comparisons
	CFMHRT			SBRT			
	No.	%	Grade X+ %	No.	%	Grade X+ %	
**Baseline GU Grade**							
Grade 0	318	79.1%	**100.0%**	295	75.6%	**100.0%**	p=0·24 Mann-Whitney comparing grade frequencies
Grade 1	74	18.4%	**20.9%**	83	21.3%	**24.4%**	
Grade 2	10	2.5%	**2.5%**	10	2.6%	**3.1%**	
Grade 3	0	0.0%	**0.0%**	2	0.5%	**0.5%**	
Grade 4	0	0.0%	**0.0%**	0	0.0%	**0.0%**	
Missing	30	N/A	**N/A**	25	N/A	**N/A**	
							**Comparisons of Grade X+ %**
**Worst GU Grade**							
Grade 0	60	13.9%	**100.0%**	83	20.0%	**100.0%**	
Grade 1	254	58.8%	**86.1%**	236	56.9%	**80.0%**	
Grade 2	111	25.7%	**27.3%**	86	20.7%	**23.1%**	−4.2% difference 95% CI −10.0 to 1.7% p=0·16 (Chi-square)
Grade 3	6	1.4%	**1.6%**	8	1.9%	**2.4%**	0.8% difference 95% CI −1.1 to 2.7% p=0·47 (Fisher's)
Grade 4	1	0.2%	**0.2%**	2	0.5%	**0.5%**	
**Worst GU Grade, Exceeding Baseline Grade**							
No Baseline Data	30	N/A	**N/A**	25	N/A	**N/A**	
Baseline Not Exceeded	116	28.9%	**100.0%**	149	38.2%	**100.0%**	
Grade 1	186	46.3%	**71.1%**	162	41.5%	**61.8%**	
Grade 2	93	23.1%	**24.9%**	69	17.7%	**20.3%**	−4.6% difference 95% CI −10.4 to 1.2% p=0.12 (Chi-square)
Grade 3	6	1.5%	**1.7%**	8	2.1%	**2.6%**	0.8% difference 95% CI −1.2 to 2.8% p=0·47 (Fisher's)
Grade 4	1	0.2%	**0.2%**	2	0.5%	**0.5%**	
**Week 12 Post-RT GU Grade**							
Grade 0	291	69.6%	**100.0%**	278	69.2%	**100.0%**	
Grade 1	112	26.8%	**30.4%**	104	25.9%	**30.8%**	
Grade 2	14	3.3%	**3.6%**	20	5.0%	**5.0%**	1.4% difference 95% CI −1.4 to 4.2% p=0·33 (Chi-square)
Grade 3	1	0.2%	**0.2%**	0	0.0%	**0.0%**	p=1.0 (Fisher's)
Grade 4	0	0.0%	**0.0%**	0	0.0%	**0.0%**	
Missing	14	N/A	**N/A**	13	N/A	**N/A**	
**Total**	**432**			**415**			

Supplementary Figure 1. Acute RTOG Toxicity By Treatment Duration: Gastrointestinal and Genitourinary

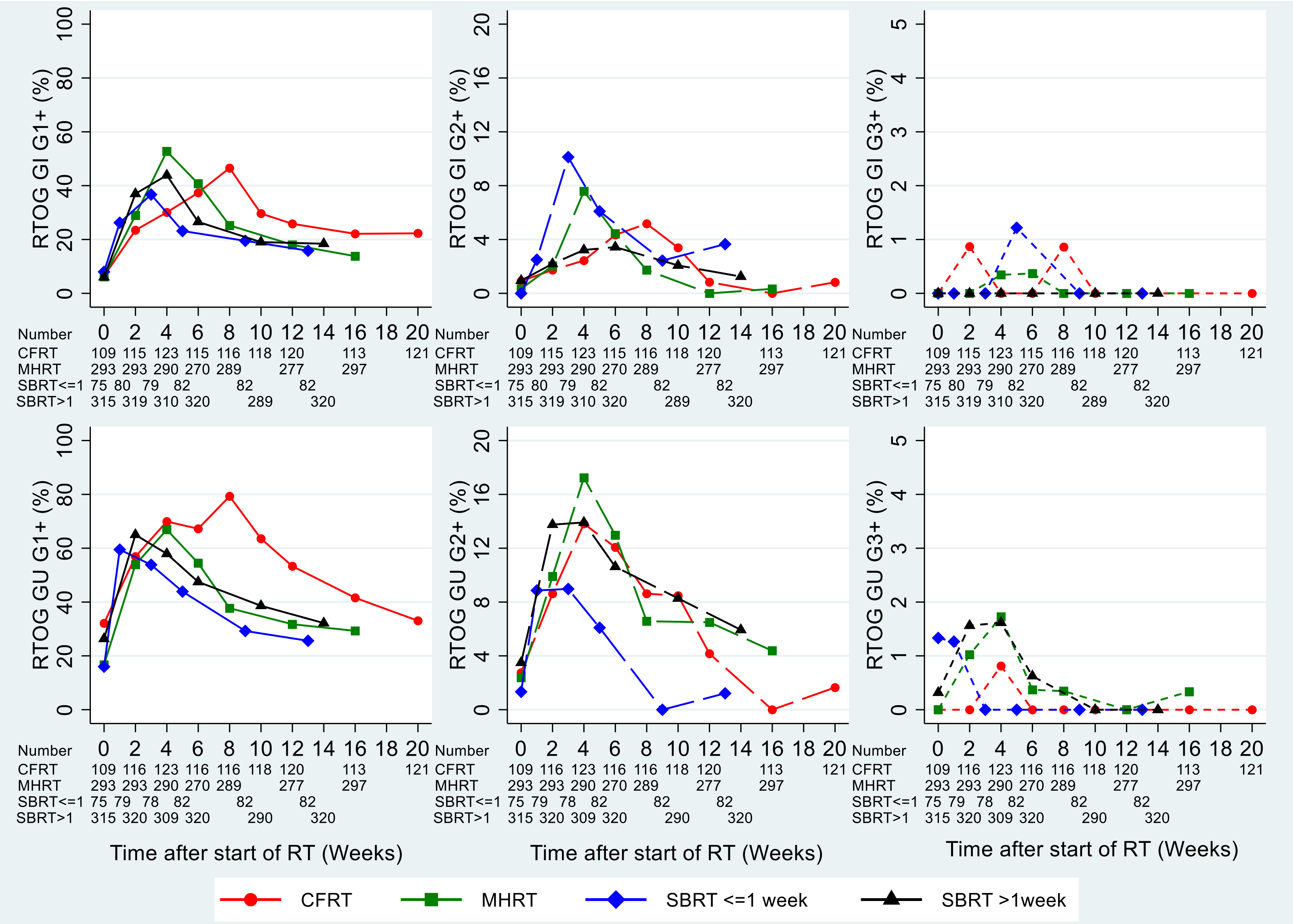

Acute RTOG toxicity, separated into four different overall treatment times permitted. For ease of display, SBRT patients receiving their treatment over more than the maximum recommended 2 weeks (n=24) are displayed in the same line as the 2-week SBRT patients. Week 0 is the baseline toxicity score taken before start of radiotherapy. Numbers at risk for each arm are asynchronous because they are shown only at data collection timepoints (which are non-simultaneous relative to the start of radiotherapy. Abbreviations: RTOG = Radiation Therapy Oncology Group; GX+ = Grade X or more; CFRT = conventionally fractionated radiotherapy; MHRT = moderately hypofractionated radiotherapy; SBRT = Stereotactic body radiotherapy; RT = radiotherapy.

Supplementary Table 13. Standardised Summary Table of CTCAE Adverse Events

Summarising the worst CTCAE grade occurring up to 12 weeks post radiotherapy (the acute toxicity window). Items included are those with ≥10% G1-2 in either arm, or any G3+ event.

CTCAE = Common Terminology Criteria for Adverse Events; G = grade; CFMHRT = conventionally fractionated or moderately hypofractionated radiotherapy; SBRT = Stereotactic body radiotherapy

CTCAE Toxicity	CFMHRT (n=432)					SBRT (n=415)				
	G1	G2	G3	G4	G5	G1	G2	G3	G4	G5
	n (%)	n (%)	n (%)	n (%)	n (%)	n (%)	n (%)	n (%)	n (%)	n (%)
**Gastrointestinal**										
Colitis	25 (5.8)	4 (0.9)	0 (0)	0 (0)	0 (0)	28 (6.7)	4 (1.0)	1 (0.2)	0 (0)	0 (0)
Constipation	62 (14.4)	14 (3.2)	0 (0)	0 (0)	0 (0)	107 (25.8)	15 (3.6)	0 (0)	0 (0)	0 (0)
Diarrhoea	107 (24.8)	4 (0.9)	2 (0.5)	0 (0)	0 (0)	154 (37.1)	26 (6.3)	1 (0.2)	0 (0)	0 (0)
Nausea	14 (3.2)	2 (0.5)	0 (0)	0 (0)	0 (0)	40 (9.6)	2 (0.5)	0 (0)	0 (0)	0 (0)
Proctitis	102 (23.6)	10 (2.3)	1 (0.2)	0 (0)	0 (0)	117 (28.2)	23 (5.5)	1 (0.2)	0 (0)	0 (0)
GI Haemorrhage	62 (14.4)	2 (0.5)	0 (0)	0 (0)	0 (0)	96 (23.1)	4 (1.0)	0 (0)	0 (0)	0 (0)
Rectal Pain	49 (11.3)	4 (0.9)	0 (0)	0 (0)	0 (0)	103 (24.8)	7 (1.7)	1 (0.2)	0 (0)	0 (0)
**Genitourinary**	**G1**	**G2**	**G3**	**G4**	**G5**	**G1**	**G2**	**G3**	**G4**	**G5**
Cystitis	180 (41.7)	19 (4.4)	0 (0)	0 (0)	0 (0)	248 (59.8)	43 (10.4)	3 (0.7)	0 (0)	0 (0)
Haematuria	14 (3.2)	1 (0.2)	1 (0.2)	0 (0)	0 (0)	37 (8.9)	3 (0.7)	0 (0)	0 (0)	0 (0)
Urinary Frequency	270 (62.5)	54 (12.5)	0 (0)	0 (0)	0 (0)	280 (67.5)	86 (20.7)	2 (0.5)	0 (0)	0 (0)
Urinary Incontinence	91 (21.1)	6 (1.4)	1 (0.2)	0 (0)	0 (0)	78 (18.8)	13 (3.1)	1 (0.2)	0 (0)	0 (0)
Urinary Retention	78 (18.1)	29 (6.7)	1 (0.2)	0 (0)	0 (0)	105 (25.3)	33 (8.0)	2 (0.5)	0 (0)	0 (0)
Urinary tract infection	0 (0)	2 (0.5)	0 (0)	1 (0.2)	0 (0)	4 (1.0)	2 (0.5)	1 (0.2)	0 (0)	0 (0)
Urinary Urgency	243 (56.3)	35 (8.1)	0 (0)	0 (0)	0 (0)	257 (61.9)	44 (10.6)	1 (0.2)	0 (0)	0 (0)
**Sexual, Hormonal & Skin**	**G1**	**G2**	**G3**	**G4**	**G5**	**G1**	**G2**	**G3**	**G4**	**G5**
Erectile Dysfunction	144 (33.3)	96 (22.2)	50 (11.6)	0 (0)	0 (0)	149 (35.9)	80 (19.3)	50 (12.0)	0 (0)	0 (0)
Fatigue	234 (54.2)	14 (3.2)	0 (0)	0 (0)	0 (0)	275 (66.3)	32 (7.7)	2 (0.5)	0 (0)	0 (0)
Radiation Dermatitis	20 (4.6)	0 (0)	1 (0.2)	0 (0)	0 (0)	17 (4.1)	0 (0)	0 (0)	0 (0)	0 (0)
**Other**	**G1**	**G2**	**G3**	**G4**	**G5**	**G1**	**G2**	**G3**	**G4**	**G5**
Arthralgia	15 (3.5)	0 (0)	0 (0)	0 (0)	0 (0)	18 (4.3)	1 (0.2)	1 (0.2)	0 (0)	0 (0)
Chest Pain (Cardiac)	0 (0)	0 (0)	0 (0)	0 (0)	0 (0)	0 (0)	0 (0)	1 (0.2)	1 (0.2)	0 (0)
Dyspnoea	2 (0.5)	0 (0)	0 (0)	0 (0)	0 (0)	3 (0.7)	1 (0.2)	1 (0.2)	0 (0)	0 (0)
Fall	0 (0)	0 (0)	0 (0)	0 (0)	0 (0)	0 (0)	0 (0)	2 (0.5)	0 (0)	0 (0)
Generalised Muscle Weakness	0 (0)	0 (0)	0 (0)	0 (0)	0 (0)	2 (0.5)	0 (0)	1 (0.2)	0 (0)	0 (0)

Hearing Impairment	0 (0)	0 (0)	0 (0)	0 (0)	0 (0)	1 (0.2)	0 (0)	1 (0.2)	0 (0)	0 (0)
Hyperkalaemia	0 (0)	0 (0)	0 (0)	0 (0)	0 (0)	0 (0)	1 (0.2)	1 (0.2)	0 (0)	0 (0)
Hypertension	1 (0.2)	6 (1.4)	3 (0.7)	0 (0)	0 (0)	1 (0.2)	8 (1.9)	1 (0.2)	0 (0)	0 (0)
Hyponatraemia	0 (0)	0 (0)	0 (0)	0 (0)	0 (0)	0 (0)	0 (0)	1 (0.2)	0 (0)	0 (0)

Supplementary Table 14. Summary of All Serious Adverse Events and Serious Adverse Reactions Occurring During Acute Toxicity Period

Abbreviations: Pt = patient; SAE = Serious Adverse Event; SAR = Serious Adverse Reaction; Fidx = Fiducials; G = grade; RT = Radiotherapy; CFMHRT = conventionally fractionated or moderately hypofractionated radiotherapy; SBRT = Stereotactic body radiotherapy

Pt	ITT	Per Protocol	Class	SAE/R Report Type	SAE Form Reported Toxicity	Fidx	Timing	Exact Sequence	Causality Evaluation	Expectedness
1	CFMHRT	CFMHRT	SAR	Hospitalisation	Sepsis (G4)	Yes	Pre-RT	1 day after fiducials	3. Possible	Expected
2	CFMHRT	SBRT	SAR	Prolongation of Hospitalisation	Urosepsis (G4)	Yes	Pre-RT	2 days after fiducials	5. Definite	Expected
3	SBRT	SBRT	SAR	Hospitalisation	Prostate infection (G3)	Yes	Pre-RT	3 days after fiducials	5. Definite	Expected
4	SBRT	SBRT	SAR	Hospitalisation	Urosepsis following gold seeds (G3)	Yes	Pre-RT	4 days after fiducials	5. Definite	Expected
**5**	SBRT	SBRT	SAR	Hospitalisation	Urosepsis (G2)<br>Pyretic 39.5°C (G2)<br>Chills (G1)	Yes	Pre-RT	7 days after fiducials	4. Probable	Expected
6	SBRT	SBRT	SAR	Other	Urinary retention (G2)	Yes	During RT	2 days after first fraction	4. Probable	Expected
7	CFMHRT	CFMHRT	SAR	Hospitalisation	Urinary Retention (G2)<br>Haematuria (G3)<br>Urinary Obstruction (G2)	No	During RT	2 days after first fraction	4. Probable	Expected
8	SBRT	SBRT	SAR	Hospitalisation	Haematuria (G2)<br>Urinary Retention (G2)	No	During RT	11 days after first fraction	4. Probable	Expected
9	CFMHRT	CFMHRT	SAR	Hospitalisation	Urinary retention (G2)	Yes	During RT	23 days after first fraction	4. Probable	Expected
10	CFMHRT	CFMHRT	SAR	Hospitalisation	Haematuria (G2)	No	Post-RT	2 days after last fraction	4. Probable	Expected
11	SBRT	SBRT	SAE	Hospitalisation	Chest pain (G3)	No	Post-RT	6 days after last fraction	2. Unlikely	N/A
**5**	SBRT	SBRT	SAR	Hospitalisation	Urinary tract infection (G3)<br>Hyponatraemia (G3)<br>Hyperkalaemia (G3)	Yes	Post-RT	6 days after last fraction	4. Probable	Expected
12	SBRT	SBRT	SAR	Other	Urinary retention (G2)	Yes	Post-RT	8 days after last fraction	4. Probable	Expected
13	SBRT	SBRT	SAR	Hospitalisation	Urinary retention (G2)	No	Post-RT	15 days after last fraction	4. Probable	Expected
14	CFMHRT	CFMHRT	SAR	Life Threatening	Urosepsis (G4)	Yes	Post-RT	27 days after last fraction	4. Probable	Expected
15	SBRT	SBRT	SAE	Hospitalisation	Cardiac Arrest (G4)	Yes	Post RT	42 days after last fraction	1. Unrelated	N/A
16	SBRT	SBRT	SAR	Other	Rectal pain (G3)	Yes	Post-RT	44 days after last fraction	3. Possible	Expected

Supplementary Figure 2. Acute CTCAE Toxicity By Treatment Duration: Gastrointestinal and Genitourinary

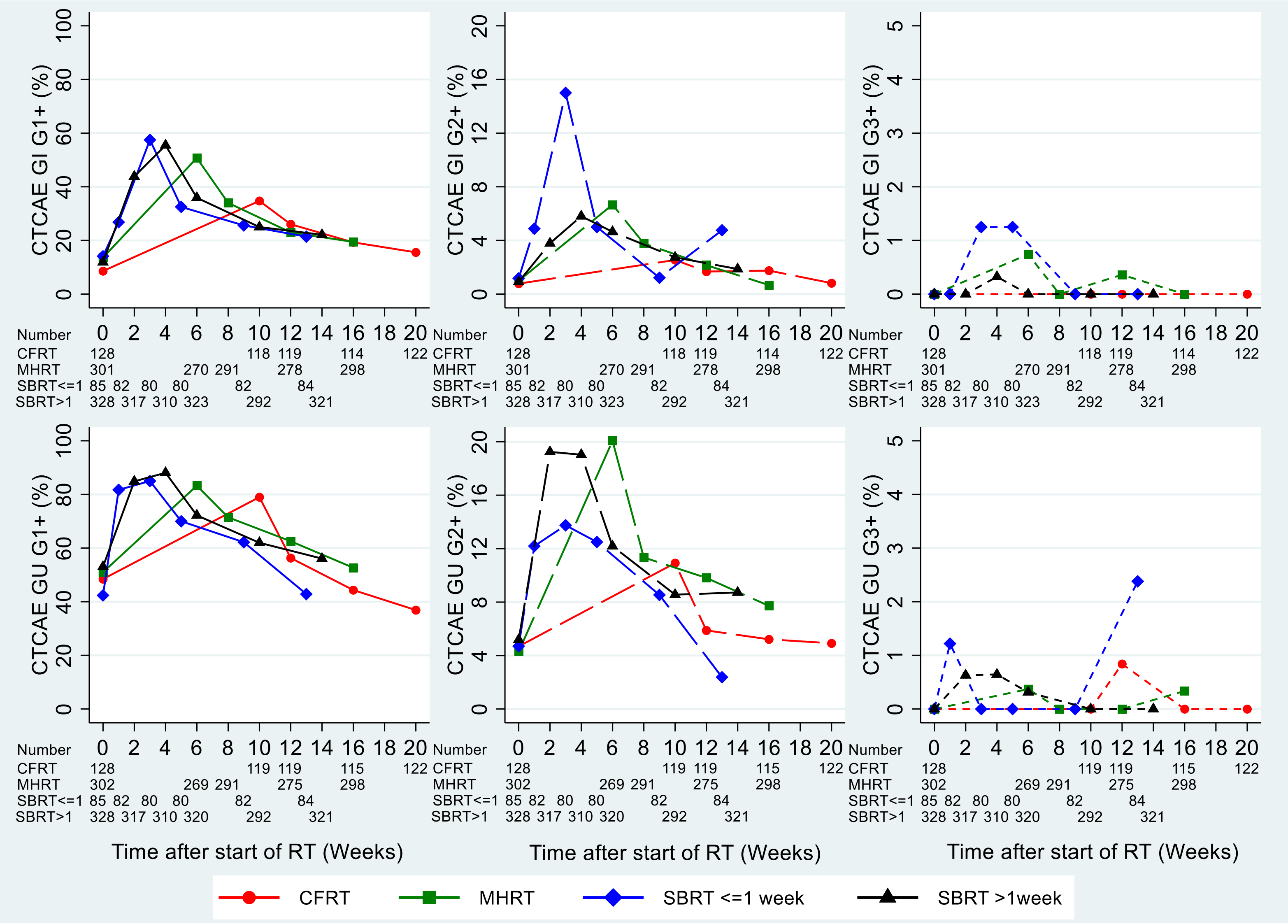

Acute CTCAE toxicity, separated into four different overall treatment times permitted. For ease of display, SBRT patients receiving their treatment over more than the maximum recommended 2 weeks (n=24) are displayed in the same line as the 2-week SBRT patients. Week 0 is the baseline toxicity score taken before start of radiotherapy. Numbers at risk for each arm are asynchronous because they are shown only at data collection timepoints (which are non-simultaneous relative to the start of radiotherapy. Abbreviations: CTCAE = Common Terminology Criteria for Adverse Events; GX+ = Grade X or more; CFRT = conventionally fractionated radiotherapy; MHRT = moderately hypofractionated radiotherapy; SBRT = Stereotactic body radiotherapy; RT = radiotherapy.

Supplementary Table 15. Summary of CTCAE GI Composite Toxicity

CTCAE Gastrointestinal (GI) Composite Toxicity	Per Protocol Treatment						Statistical Comparisons
	CFMHRT			SBRT			
	**No.**	**%**	**Grade X+ %**	**No.**	**%**	**Grade X+ %**	
**Baseline**							
Grade 0	377	87.9%	100.0%	362	87.7%	100.0%	p=0·92 Mann-Whitney comparing grade frequencies
Grade 1	48	11.2%	12.1%	47	11.4%	12.3%	
Grade 2	4	0.9%	0.9%	4	1.0%	1.0%	
Grade 3	0	0.0%	0.0%	0	0.0%	0.0%	
Grade 4	0	0.0%	0.0%	0	0.0%	0.0%	
Not Graded	3	N/A	N/A	2	N/A	N/A	
							**Comparisons of Grade X+ %**
**Worst**							
Grade 0	181	42.1%	100.0%	109	26.3%	100.0%	
Grade 1	213	49.5%	57.9%	241	58.1%	73.7%	
Grade 2	33	7.7%	8.4%	62	14.9%	15.7%	Difference 7.3%<br>95% CI 2.9 to 11.7%<br>**p=0.0011 (Chi-square)**
Grade 3	3	0.7%	0.7%	3	0.7%	0.7%	Difference 0.03%<br>95% CI −1.1 to 1.2%<br>p=1.0 (Fisher's)
Grade 4	0	0.0%	0.0%	0	0.0%	0.0%	
Not Graded	2	N/A	N/A	0	N/A	N/A	
**Worst, Exceeding Baseline**							
Baseline Not Exceeded	212	49.6%	100.0%	145	35.1%	100.0%	
Grade 1	181	42.4%	50.4%	205	49.6%	64.9%	
Grade 2	31	7.3%	8.0%	60	14.5%	15.3%	Difference 7.3%<br>95% CI 3.0 to 11.6%<br>**p=0.00095 (Chi-square)**
Grade 3	3	0.7%	0.7%	3	0.7%	0.7%	Difference 0.02%<br>95% CI −1.1 to 1.2%<br>p=1.0 (Fisher's)
Grade 4	0	0.0%	0.0%	0	0.0%	0.0%	
Missing Data	5	N/A	N/A	2	N/A	N/A	
**Week 12 Post-RT**							
Grade 0	343	81.7%	100.0%	316	78.0%	100.0%	
Grade 1	74	17.6%	18.3%	79	19.5%	22.0%	
Grade 2	3	0.7%	0.7%	10	2.5%	2.5%	Difference 1.8%<br>95% CI 0.04 to 3.4%<br>p=0.052 (Fisher's)
Grade 3	0	0.0%	0.0%	0	0.0%	0.0%	N/A
Grade 4	0	0.0%	0.0%	0	0.0%	0.0%	
Not Graded	12	N/A	N/A	10	N/A	N/A	
**Total**	**432**			**415**			

Supplementary Table 16. Summary of CTCAE GU Composite Toxicity

CTCAE Genitourinary (GU) Composite Toxicity	Per Protocol Treatment						Statistical Comparisons
	CFMHRT			SBRT			
	No.	%	Grade X+ %	No.	%	Grade X+ %	
**Baseline**							
Grade 0	214	49.8%	100.0%	203	49.2%	100.0%	p=0.79 Mann-Whitney comparing grade frequencies
Grade 1	197	45.8%	50.2%	189	45.8%	50.8%	
Grade 2	19	4.4%	4.4%	21	5.1%	5.1%	
Grade 3	0	0.0%	0.0%	0	0.0%	0.0%	
Grade 4	0	0.0%	0.0%	0	0.0%	0.0%	
Not Graded	2	N/A	N/A	2	N/A	N/A	
							**Comparisons of Grade X+ %**
**Worst**							
Grade 0	48	11.2%	100.0%	15	3.6%	100.0%	
Grade 1	283	65.8%	88.8%	272	65.5%	96.4%	
Grade 2	96	22.3%	23.0%	121	29.2%	30.8%	Difference 7.8% 95% CI 1.9 to 13.8 p=0.010 (Chi-square)
Grade 3	3	0.7%	0.7%	7	1.7%	1.7%	Difference 1.0% 95% CI −0.5 to 2.5% p=0.22 (Fisher's)
Grade 4	0	0.0%	0.0%	0	0.0%	0.0%	
Not Graded	2	N/A	N/A	0	N/A	N/A	
**Worst, Exceeding Baseline**							
Baseline Not Exceeded	198	46.3%	100.0%	164	39.7%	100.0%	
Grade 1	142	33.2%	53.7%	136	32.9%	60.3%	
Grade 2	85	19.9%	20.6%	106	25.7%	27.4%	Difference 6.8% 95% CI 1.0 to 12.6% p=0.021 (Chi-square)
Grade 3	3	0.7%	0.7%	7	1.7%	1.7%	Difference 1.0% 95% CI −0.5 to 2.5% p=0.22 (Fisher's)
Grade 4	0	0.0%	0.0%	0	0.0%	0.0%	
Missing Data	4	N/A	N/A	2	N/A	N/A	
**Week 12 Post-RT**							
Grade 0	218	51.9%	100.0%	189	46.7%	100.0%	
Grade 1	173	41.2%	48.1%	186	45.9%	53.3%	
Grade 2	28	6.7%	6.9%	28	6.9%	7.4%	Difference 0.5% 95% CI −3.0 to 4.0 p=0.78 (Chi-square)
Grade 3	1	0.2%	0.2%	2	0.5%	0.5%	Difference 0.3% 95% CI −0.6 to 1.1% p=0.62 (Fisher's)
Grade 4	0	0.0%	0.0%	0	0.0%	0.0%	
Not Graded	12	N/A	N/A	10	N/A	N/A	
**Total**	**431**			**414**			

Supplementary Table 17. Baseline CTCAE Gastrointestinal (GI) Toxicity

Note that some items were not explicitly asked for, instead being retrieved from free text CTCAE reporting. Hence high numbers of "non-graded" entries for these items (diverticulitis, haemorrhoids). Hence, patients not graded are excluded from percentage calculation for the composite score, but not from individual toxicity items.

Baseline CTCAE GI Toxicity	Per Protocol Treatment				Total	
	CFMHRT		SBRT			
	No.	%	No.	%	No.	%
**Composite GI**						
Not Graded	3	N/A	2	N/A	5	N/A
Grade 0	377	87.9%	362	87.7%	739	87.8%
Grade 1	48	11.2%	47	11.4%	95	11.3%
Grade 2	4	0.9%	4	1.0%	8	1.0%
Grade 3	0	0.0%	0	0.0%	0	0.0%
Grade 4	0	0.0%	0	0.0%	0	0.0%
**GI COMPOSITE FORMED FROM WORST OF FOLLOWING ITEMS**						
**Colitis**						
Not Graded	3	0.7%	2	0.5%	5	0.6%
Grade 0	428	99.1%	409	98.6%	837	98.8%
Grade 1	1	0.2%	3	0.7%	4	0.5%
Grade 2	0	0.0%	1	0.2%	1	0.1%
**Constipation**						
Not Graded	3	0.7%	2	0.5%	5	0.6%
Grade 0	404	93.5%	383	92.3%	787	92.9%
Grade 1	22	5.1%	29	7.0%	51	6.0%
Grade 2	3	0.7%	1	0.2%	4	0.5%
**Diarrhoea**						
Not Graded	3	0.7%	2	0.5%	5	0.6%
Grade 0	418	96.8%	402	96.9%	820	96.8%
Grade 1	11	2.5%	10	2.4%	21	2.5%
Grade 2	0	0.0%	1	0.2%	1	0.1%
**Diverticulitis**						
Not Graded	430	99.5%	415	100.0%	845	99.8%
Grade 1	1	0.2%	0	0.0%	1	0.1%
Grade 2	1	0.2%	0	0.0%	1	0.1%
**Fistula**						
Not Graded	4	0.9%	2	0.5%	6	0.7%
Grade 0	427	98.8%	413	99.5%	840	99.2%
Grade 1	1	0.2%	0	0.0%	1	0.1%
**Gastrointestinal pain**						
Not Graded	430	99.5%	413	99.5%	843	99.5%
Grade 1	2	0.5%	1	0.2%	3	0.4%
Grade 2	0	0.0%	1	0.2%	1	0.1%

**Haemorrhoids**						
Not Graded	432	100.0%	414	99.8%	846	99.9%
Grade 1	0	0.0%	1	0.2%	1	0.1%
**GI Haemorrhage**						
Not Graded	4	0.9%	3	0.7%	7	0.8%
Grade 0	421	97.5%	410	98.8%	831	98.1%
Grade 1	7	1.6%	2	0.5%	9	1.1%
**Proctitis**						
Not Graded	3	0.7%	3	0.7%	6	0.7%
Grade 0	427	98.8%	407	98.1%	834	98.5%
Grade 1	2	0.5%	5	1.2%	7	0.8%
**Rectal Pain**						
Not Graded	4	0.9%	3	0.7%	7	0.8%
Grade 0	423	97.9%	406	97.8%	829	97.9%
Grade 1	5	1.2%	6	1.4%	11	1.3%
**Total**	**432**	**100.0%**	**415**	**100.0%**	**847**	**100.0%**

Supplementary Table 18. Baseline CTCAE Genitourinary (GU) Toxicity

Note that some items were not explicitly asked for, instead being retrieved from free text CTCAE reporting. Hence high numbers of "non-graded" entries for these items (bladder spasm, prostatic obstruction). Hence, patients not graded are excluded from percentage calculation for the composite score, but not from individual toxicity items.

Baseline CTCAE GU Toxicity	Per Protocol Treatment				Total	
	CFMHRT		SBRT			
	No.	%	No.	%	No.	%
**Composite GU**						
Not Graded	2	N/A	2	N/A	4	N/A
Grade 0	214	49.8%	203	49.2%	417	49.5%
Grade 1	197	45.8%	189	45.8%	386	45.8%
Grade 2	19	4.4%	21	5.1%	40	4.7%
Grade 3	0	0.0%	0	0.0%	0	0.0%
Grade 4	0	0.0%	0	0.0%	0	0.0%
**GU COMPOSITE FORMED FROM WORST OF FOLLOWING ITEMS**						
**Bladder Spasm**						
Not Graded	431	99.8%	415	100.0%	846	99.9%
Grade 2	1	0.2%	0	0.0%	1	0.1%
**Cystitis**						
Not Graded	3	0.7%	2	0.5%	5	0.6%
Grade 0	398	92.1%	383	92.3%	781	92.2%
Grade 1	31	7.2%	28	6.7%	59	7.0%
Grade 2	0	0.0%	2	0.5%	2	0.2%
**Haematuria**						
Not Graded	4	0.9%	2	0.5%	6	0.7%
Grade 0	426	98.6%	407	98.1%	833	98.3%
Grade 1	1	0.2%	6	1.4%	7	0.8%
Grade 2	1	0.2%	0	0.0%	1	0.1%
**Prostatic obstruction**						
Not Graded	430	99.5%	412	99.3%	842	99.4%
Grade 1	2	0.5%	3	0.7%	5	0.6%
**Urinary Frequency**						
Not Graded	4	0.9%	3	0.7%	7	0.8%
Grade 0	271	62.7%	260	62.7%	531	62.7%
Grade 1	149	34.5%	143	34.5%	292	34.5%
Grade 2	8	1.9%	9	2.2%	17	2.0%
**Urinary Incontinence**						
Not Graded	3	0.7%	2	0.5%	5	0.6%
Grade 0	399	92.4%	388	93.5%	787	92.9%
Grade 1	29	6.7%	23	5.5%	52	6.1%
Grade 2	1	0.2%	2	0.5%	3	0.4%
**Urinary Retention**						
Not Graded	3	0.7%	2	0.5%	5	0.6%

Grade 0	387	89.6%	381	91.8%	768	90.7%
Grade 1	34	7.9%	22	5.3%	56	6.6%
Grade 2	8	1.9%	10	2.4%	18	2.1%
**Urinary Urgency**						
Not Graded	2	0.5%	3	0.7%	5	0.6%
Grade 0	345	79.9%	309	74.5%	654	77.2%
Grade 1	79	18.3%	102	24.6%	181	21.4%
Grade 2	6	1.4%	1	0.2%	7	0.8%
**Total**	**432**	**100.0%**	**415**	**100.0%**	**847**	**100.0%**

Supplementary Table 19. Worst Acute CTCAE Gastrointestinal (GI) Toxicity

Note that some items were not explicitly asked for, instead being retrieved from free text CTCAE reporting. Hence high numbers of "non-graded" entries for these items (anal pain, diverticulitis, faecal incontinence, haemorrhoids, GI unspecified, rectal prolapse). Hence, patients not graded are excluded from percentage calculation for the composite score, but not from individual toxicity items.

Worst Acute CTCAE GI Toxicity	Per Protocol Treatment				Total	
	CFMHRT		SBRT			
	No.	%	No.	%	No.	%
**Composite GI**						
Not Graded	2	N/A	0	N/A	2	N/A
Grade 0	181	42.1%	109	26.3%	290	34.3%
Grade 1	213	49.5%	241	58.1%	454	53.7%
Grade 2	33	7.7%	62	14.9%	95	11.2%
Grade 3	3	0.7%	3	0.7%	6	0.7%
**GI COMPOSITE FORMED FROM WORST OF FOLLOWING ITEMS**						
**Anal Pain**						
Not Graded	431	99.8%	412	99.3%	843	99.5%
Grade 1	1	0.2%	3	0.7%	4	0.5%
**Colitis**						
Not Graded	2	0.5%	1	0.2%	3	0.4%
Grade 0	401	92.8%	381	91.8%	782	92.3%
Grade 1	25	5.8%	28	6.7%	53	6.3%
Grade 2	4	0.9%	4	1.0%	8	0.9%
Grade 3	0	0.0%	1	0.2%	1	0.1%
**Constipation**						
Not Graded	2	0.5%	1	0.2%	3	0.4%
Grade 0	354	81.9%	292	70.4%	646	76.3%
Grade 1	62	14.4%	107	25.8%	169	20.0%
Grade 2	14	3.2%	15	3.6%	29	3.4%
**Diarrhoea**						
Not Graded	2	0.5%	1	0.2%	3	0.4%
Grade 0	317	73.4%	233	56.1%	550	64.9%
Grade 1	107	24.8%	154	37.1%	261	30.8%
Grade 2	4	0.9%	26	6.3%	30	3.5%
Grade 3	2	0.5%	1	0.2%	3	0.4%
**Diverticulitis**						
Not Graded	431	99.8%	415	100.0%	846	99.9%
Grade 2	1	0.2%	0	0.0%	1	0.1%
**Faecal incontinence**						
Not Graded	431	99.8%	411	99.0%	842	99.4%
Grade 1	1	0.2%	2	0.5%	3	0.4%
Grade 2	0	0.0%	2	0.5%	2	0.2%
**Fistula**						
Not Graded	7	1.6%	2	0.5%	9	1.1%

Grade 0	424	98.1%	411	99.0%	835	98.6%
Grade 1	1	0.2%	2	0.5%	3	0.4%
**Gastrointestinal pain**						
Not Graded	421	97.5%	399	96.1%	820	96.8%
Grade 1	11	2.5%	15	3.6%	26	3.1%
Grade 2	0	0.0%	1	0.2%	1	0.1%
**Haemorrhoids**						
Not Graded	426	98.6%	413	99.5%	839	99.1%
Grade 1	5	1.2%	2	0.5%	7	0.8%
Grade 2	1	0.2%	0	0.0%	1	0.1%
**GI Haemorrhage**						
Not Graded	2	0.5%	1	0.2%	3	0.4%
Grade 0	366	84.7%	314	75.7%	680	80.3%
Grade 1	62	14.4%	96	23.1%	158	18.7%
Grade 2	2	0.5%	4	1.0%	6	0.7%
**Proctitis**						
Not Graded	2	0.5%	0	0.0%	2	0.2%
Grade 0	317	73.4%	274	66.0%	591	69.8%
Grade 1	102	23.6%	117	28.2%	219	25.9%
Grade 2	10	2.3%	23	5.5%	33	3.9%
Grade 3	1	0.2%	1	0.2%	2	0.2%
**Rectal Pain**						
Not Graded	2	0.5%	1	0.2%	3	0.4%
Grade 0	377	87.3%	303	73.0%	680	80.3%
Grade 1	49	11.3%	103	24.8%	152	17.9%
Grade 2	4	0.9%	7	1.7%	11	1.3%
Grade 3	0	0.0%	1	0.2%	1	0.1%
**GI Unspecified**						
Not Graded	432	100.0%	414	99.8%	846	99.9%
Grade 1	0	0.0%	1	0.2%	1	0.1%
**Rectal Prolapse**						
Not Graded	432	100.0%	414	99.8%	846	99.9%
Grade 1	0	0.0%	1	0.2%	1	0.1%
**Total**	**432**	**100.0%**	**415**	**100.0%**	**847**	**100.0%**

Supplementary Table 20. Worst Acute CTCAE Genitourinary (GU) Toxicity

Note that some items were not explicitly asked for, instead being retrieved from free text CTCAE reporting. Hence high numbers of "non-graded" entries for these items (bladder spasm, prostatic obstruction, urethral stricture). Hence, patients not graded are excluded from percentage calculation for the composite score, but not from individual toxicity items.

Worst Acute CTCAE GU Toxicity	Per Protocol Treatment				Total	
	CFMHRT		SBRT			
	No.	%	No.	%	No.	%
**Composite GU**						
Not Graded	2	N/A	0	N/A	2	N/A
Grade 0	48	11.2%	15	3.6%	63	7.5%
Grade 1	283	65.8%	272	65.5%	555	65.7%
Grade 2	96	22.3%	121	29.2%	217	25.7%
Grade 3	3	0.7%	7	1.7%	10	1.2%
**GU COMPOSITE FORMED FROM WORST OF FOLLOWING ITEMS**						
**Bladder Spasm**						
Not Graded	429	99.3%	414	99.8%	843	99.5%
Grade 1	0	0.0%	1	0.2%	1	0.1%
Grade 2	3	0.7%	0	0.0%	3	0.4%
**Cystitis**						
Not Graded	2	0.5%	1	0.2%	3	0.4%
Grade 0	231	53.5%	120	28.9%	351	41.4%
Grade 1	180	41.7%	248	59.8%	428	50.5%
Grade 2	19	4.4%	43	10.4%	62	7.3%
Grade 3	0	0.0%	3	0.7%	3	0.4%
**Haematuria**						
Not Graded	2	0.5%	1	0.2%	3	0.4%
Grade 0	414	95.8%	374	90.1%	788	93.0%
Grade 1	14	3.2%	37	8.9%	51	6.0%
Grade 2	1	0.2%	3	0.7%	4	0.5%
Grade 3	1	0.2%	0	0.0%	1	0.1%
**Prostatic obstruction**						
Not Graded	414	95.8%	388	93.5%	802	94.7%
Grade 1	17	3.9%	24	5.8%	41	4.8%
Grade 2	1	0.2%	3	0.7%	4	0.5%
**Urinary Frequency**						
Not Graded	2	0.5%	0	0.0%	2	0.2%
Grade 0	106	24.5%	47	11.3%	153	18.1%
Grade 1	270	62.5%	280	67.5%	550	64.9%
Grade 2	54	12.5%	86	20.7%	140	16.5%
Grade 3	0	0.0%	2	0.5%	2	0.2%
**Urinary Incontinence**						
Not Graded	2	0.5%	1	0.2%	3	0.4%
Grade 0	332	76.9%	322	77.6%	654	77.2%

Grade 1	91	21.1%	78	18.8%	169	20.0%
Grade 2	6	1.4%	13	3.1%	19	2.2%
Grade 3	1	0.2%	1	0.2%	2	0.2%
**Urinary Retention**						
Not Graded	2	0.5%	1	0.2%	3	0.4%
Grade 0	322	74.5%	274	66.0%	596	70.4%
Grade 1	78	18.1%	105	25.3%	183	21.6%
Grade 2	29	6.7%	33	8.0%	62	7.3%
Grade 3	1	0.2%	2	0.5%	3	0.4%
**Urinary Urgency**						
Not Graded	2	0.5%	1	0.2%	3	0.4%
Grade 0	152	35.2%	112	27.0%	264	31.2%
Grade 1	243	56.3%	257	61.9%	500	59.0%
Grade 2	35	8.1%	44	10.6%	79	9.3%
Grade 3	0	0.0%	1	0.2%	1	0.1%
**Urethral Stricture**						
Not Graded	431	99.8%	415	100.0%	846	99.9%
Grade 1	1	0.2%	0	0.0%	1	0.1%
**Total**	**432**	**100.0%**	**415**	**100.0%**	**847**	**100.0%**

Supplementary Table 21. Worst (Exceeding Baseline) Acute CTCAE Gastrointestinal (GI) Toxicity
Note that some items were not explicitly asked for, instead being retrieved from free text CTCAE reporting. Hence high numbers of "non-graded" entries for these items (anal pain, diverticulitis, faecal incontinence, haemorrhoids, GI unspecified, rectal prolapse). Hence, patients not graded are excluded from percentage calculation for the composite score, but not from individual toxicity items. Missing data indicates either baseline or worst follow-up scores missing.

Worst (Exceeding Baseline) Acute CTCAE GI Toxicity	Per Protocol Treatment				Total	
	CFMHRT		SBRT			
	No.	%	No.	%	No.	%
**Composite GI**						
Baseline Not Exceeded	212	49.6%	145	35.1%	357	42.5%
Grade 1	181	42.4%	205	49.6%	386	46.0%
Grade 2	31	7.3%	60	14.5%	91	10.8%
Grade 3	3	0.7%	3	0.7%	6	0.7%
Missing Data	5	N/A	2	N/A	7	N/A
**GI COMPOSITE FORMED FROM WORST OF FOLLOWING ITEMS**						
**Anal Pain**						
Grade 1	1	0.2%	3	0.7%	4	0.5%
Missing Data	431	99.8%	412	99.3%	843	99.5%
**Colitis**						
Baseline Not Exceeded	401	92.8%	384	92.5%	785	92.7%
Grade 1	25	5.8%	25	6.0%	50	5.9%
Grade 2	4	0.9%	4	1.0%	8	0.9%
Grade 3	0	0.0%	1	0.2%	1	0.1%
Missing Data	2	0.5%	1	0.2%	3	0.4%
**Constipation**						
Baseline Not Exceeded	368	85.2%	309	74.5%	677	79.9%
Grade 1	49	11.3%	90	21.7%	139	16.4%
Grade 2	13	3.0%	15	3.6%	28	3.3%
Missing Data	2	0.5%	1	0.2%	3	0.4%
**Diarrhoea**						
Baseline Not Exceeded	326	75.5%	235	56.6%	561	66.2%
Grade 1	98	22.7%	152	36.6%	250	29.5%
Grade 2	4	0.9%	26	6.3%	30	3.5%
Grade 3	2	0.5%	1	0.2%	3	0.4%
Missing Data	2	0.5%	1	0.2%	3	0.4%
**Diverticulitis**						
Baseline Not Exceeded	1	0.2%	0	0.0%	1	0.1%
Missing Data	431	99.8%	415	100.0%	846	99.9%
**Faecal incontinence**						
Grade 1	1	0.2%	2	0.5%	3	0.4%
Grade 2	0	0.0%	2	0.5%	2	0.2%
Missing Data	431	99.8%	411	99.0%	842	99.4%

**Fistula**						
Baseline Not Exceeded	424	98.1%	411	99.0%	835	98.6%
Grade 1	1	0.2%	2	0.5%	3	0.4%
Missing Data	7	1.6%	2	0.5%	9	1.1%
**Gastrointestinal pain**						
Baseline Not Exceeded	1	0.2%	1	0.2%	2	0.2%
Grade 1	10	2.3%	15	3.6%	25	3.0%
Missing Data	421	97.5%	399	96.1%	820	96.8%
**Haemorrhoids**						
Baseline Not Exceeded	0	0.0%	1	0.2%	1	0.1%
Grade 1	5	1.2%	1	0.2%	6	0.7%
Grade 2	1	0.2%	0	0.0%	1	0.1%
Missing Data	426	98.6%	413	99.5%	839	99.1%
**GI Haemorrhage**						
Baseline Not Exceeded	367	85.0%	315	75.9%	682	80.5%
Grade 1	61	14.1%	95	22.9%	156	18.4%
Grade 2	2	0.5%	4	1.0%	6	0.7%
Missing Data	2	0.5%	1	0.2%	3	0.4%
**Proctitis**						
Baseline Not Exceeded	318	73.6%	276	66.5%	594	70.1%
Grade 1	101	23.4%	115	27.7%	216	25.5%
Grade 2	10	2.3%	23	5.5%	33	3.9%
Grade 3	1	0.2%	1	0.2%	2	0.2%
Missing Data	2	0.5%	0	0.0%	2	0.2%
**Rectal Pain**						
Baseline Not Exceeded	378	87.5%	308	74.2%	686	81.0%
Grade 1	48	11.1%	98	23.6%	146	17.2%
Grade 2	4	0.9%	7	1.7%	11	1.3%
Grade 3	0	0.0%	1	0.2%	1	0.1%
Missing Data	2	0.5%	1	0.2%	3	0.4%
**GI Unspecified**						
Grade 1	0	0.0%	1	0.2%	1	0.1%
Missing Data	432	100.0%	414	99.8%	846	99.9%
**Rectal Prolapse**						
Grade 1	0	0.0%	1	0.2%	1	0.1%
Missing Data	432	100.0%	414	99.8%	846	99.9%
**Total**	**432**	**100.0%**	**415**	**100.0%**	**847**	**100.0%**

Supplementary Table 22. Worst (Exceeding Baseline) Acute CTCAE Genitourinary (GU) Toxicity

Note that some items were not explicitly asked for, instead being retrieved from free text CTCAE reporting. Hence high numbers of "non-graded" entries for these items (bladder spasm, prostatic obstruction, urethral stricture). Hence, patients not graded are excluded from percentage calculation for the composite score, but not from individual toxicity items.

Missing data indicates either baseline or worst follow-up scores missing.

Worst (Exceeding Baseline) Acute CTCAE GU Toxicity	Per Protocol Treatment				Total	
	CFMHRT		SBRT			
	No.	%	No.	%	No.	%
**Composite GU**						
Baseline Not Exceeded	198	46.3%	164	39.7%	362	43.0%
Grade 1	142	33.2%	136	32.9%	278	33.1%
Grade 2	85	19.9%	106	25.7%	191	22.7%
Grade 3	3	0.7%	7	1.7%	10	1.2%
Missing Data	4	N/A	2	N/A	6	N/A
**GU COMPOSITE FORMED FROM WORST OF FOLLOWING ITEMS**						
**Bladder Spasm**						
Baseline Not Exceeded	1	0.2%	0	0.0%	1	0.1%
Grade 1	0	0.0%	1	0.2%	1	0.1%
Grade 2	2	0.5%	0	0.0%	2	0.2%
Missing Data	429	99.3%	414	99.8%	843	99.5%
**Cystitis**						
Baseline Not Exceeded	250	57.9%	142	34.2%	392	46.3%
Grade 1	161	37.3%	228	54.9%	389	45.9%
Grade 2	19	4.4%	41	9.9%	60	7.1%
Grade 3	0	0.0%	3	0.7%	3	0.4%
Missing Data	2	0.5%	1	0.2%	3	0.4%
**Haematuria**						
Baseline Not Exceeded	415	96.1%	375	90.4%	790	93.3%
Grade 1	13	3.0%	36	8.7%	49	5.8%
Grade 2	1	0.2%	3	0.7%	4	0.5%
Grade 3	1	0.2%	0	0.0%	1	0.1%
Missing Data	2	0.5%	1	0.2%	3	0.4%
**Prostatic obstruction**						
Baseline Not Exceeded	0	0.0%	3	0.7%	3	0.4%
Grade 1	17	3.9%	21	5.1%	38	4.5%
Grade 2	1	0.2%	3	0.7%	4	0.5%
Missing Data	414	95.8%	388	93.5%	802	94.7%
**Urinary Frequency**						
Baseline Not Exceeded	215	49.8%	160	38.6%	375	44.3%
Grade 1	164	38.0%	172	41.4%	336	39.7%
Grade 2	51	11.8%	81	19.5%	132	15.6%
Grade 3	0	0.0%	2	0.5%	2	0.2%

Missing Data	2	0.5%	0	0.0%	2	0.2%
**Urinary Incontinence**						
Baseline Not Exceeded	353	81.7%	337	81.2%	690	81.5%
Grade 1	70	16.2%	63	15.2%	133	15.7%
Grade 2	6	1.4%	13	3.1%	19	2.2%
Grade 3	1	0.2%	1	0.2%	2	0.2%
Missing Data	2	0.5%	1	0.2%	3	0.4%
**Urinary Retention**						
Baseline Not Exceeded	345	79.9%	296	71.3%	641	75.7%
Grade 1	61	14.1%	92	22.2%	153	18.1%
Grade 2	23	5.3%	24	5.8%	47	5.5%
Grade 3	1	0.2%	2	0.5%	3	0.4%
Missing Data	2	0.5%	1	0.2%	3	0.4%
**Urinary Urgency**						
Baseline Not Exceeded	205	47.5%	193	46.5%	398	47.0%
Grade 1	191	44.2%	176	42.4%	367	43.3%
Grade 2	34	7.9%	44	10.6%	78	9.2%
Grade 3	0	0.0%	1	0.2%	1	0.1%
Missing Data	2	0.5%	1	0.2%	3	0.4%
**Urethral Stricture**						
Grade 1	1	0.2%	0	0.0%	1	0.1%
Missing Data	431	99.8%	415	100.0%	846	99.9%
**Total**	**432**	**100.0%**	**415**	**100.0%**	**847**	**100.0%**

Supplementary Table 23. Week 12 Acute CTCAE Gastrointestinal (GI) Toxicity

Note that some items were not explicitly asked for, instead being retrieved from free text CTCAE reporting. Hence high numbers of "non-graded" entries for these items (faecal incontinence, haemorrhoids, GI unspecified, rectal prolapse). Hence, patients not graded are excluded from percentage calculation for the composite score, but not from individual toxicity items.

Week 12 Acute CTCAE GI Toxicity	Per Protocol Treatment				Total	
	CFMHRT		SBRT			
	No.	%	No.	%	No.	%
**Composite GI**						
Not Graded	12	N/A	10	N/A	22	N/A
Grade 0	343	81.7%	316	78.0%	659	79.9%
Grade 1	74	17.6%	79	19.5%	153	18.5%
Grade 2	3	0.7%	10	2.5%	13	1.6%
**GI COMPOSITE FORMED FROM WORST OF FOLLOWING ITEMS**						
**Colitis**						
Not Graded	14	3.2%	10	2.4%	24	2.8%
Grade 0	412	95.4%	401	96.6%	813	96.0%
Grade 1	6	1.4%	4	1.0%	10	1.2%
**Constipation**						
Not Graded	13	3.0%	10	2.4%	23	2.7%
Grade 0	400	92.6%	380	91.6%	780	92.1%
Grade 1	17	3.9%	22	5.3%	39	4.6%
Grade 2	2	0.5%	3	0.7%	5	0.6%
**Diarrhoea**						
Not Graded	12	2.8%	10	2.4%	22	2.6%
Grade 0	396	91.7%	371	89.4%	767	90.6%
Grade 1	24	5.6%	31	7.5%	55	6.5%
Grade 2	0	0.0%	3	0.7%	3	0.4%
**Faecal incontinence**						
Not Graded	431	99.8%	415	100.0%	846	99.9%
Grade 1	1	0.2%	0	0.0%	1	0.1%
**Fistula**						
Not Graded	22	5.1%	17	4.1%	39	4.6%
Grade 0	410	94.9%	398	95.9%	808	95.4%
**Gastrointestinal pain**						
Not Graded	430	99.5%	413	99.5%	843	99.5%
Grade 1	2	0.5%	2	0.5%	4	0.5%
**Haemorrhoids**						
Not Graded	432	100.0%	414	99.8%	846	99.9%
Grade 1	0	0.0%	1	0.2%	1	0.1%
**GI Haemorrhage**						
Not Graded	13	3.0%	10	2.4%	23	2.7%
Grade 0	405	93.8%	387	93.3%	792	93.5%
Grade 1	14	3.2%	17	4.1%	31	3.7%

Grade 2	0	0.0%	1	0.2%	1	0.1%
**Proctitis**						
Not Graded	12	2.8%	11	2.7%	23	2.7%
Grade 0	402	93.1%	379	91.3%	781	92.2%
Grade 1	18	4.2%	23	5.5%	41	4.8%
Grade 2	0	0.0%	2	0.5%	2	0.2%
**Rectal Pain**						
Not Graded	12	2.8%	10	2.4%	22	2.6%
Grade 0	410	94.9%	398	95.9%	808	95.4%
Grade 1	9	2.1%	6	1.4%	15	1.8%
Grade 2	1	0.2%	1	0.2%	2	0.2%
**Total**	**432**	**100.0%**	**415**	**100.0%**	**847**	**100.0%**

Supplementary Table 24. Week 12 CTCAE Genitourinary (GU) Toxicity

Note that some items were not explicitly asked for, instead being retrieved from free text CTCAE reporting. Hence high numbers of "non-graded" entries for these items (bladder spasm, prostatic obstruction). Hence, patients not graded are excluded from percentage calculation for the composite score, but not from individual toxicity items.

Week 12 Acute CTCAE GU Toxicity	Per Protocol Treatment				Total	
	CFMHRT		SBRT			
	No.	%	No.	%	No.	%
**Composite GU**						
Not Graded	12	N/A	10	N/A	22	N/A
Grade 0	218	51.9%	189	46.7%	407	49.3%
Grade 1	173	41.2%	186	45.9%	359	43.5%
Grade 2	28	6.7%	28	6.9%	56	6.8%
Grade 3	1	0.2%	2	0.5%	3	0.4%
**GU COMPOSITE FORMED FROM WORST OF FOLLOWING ITEMS**						
**Bladder Spasm**						
Not Graded	430	99.5%	415	100.0%	845	99.8%
Grade 2	2	0.5%	0	0.0%	2	0.2%
**Cystitis**						
Not Graded	12	2.8%	10	2.4%	22	2.6%
Grade 0	377	87.3%	361	87.0%	738	87.1%
Grade 1	40	9.3%	41	9.9%	81	9.6%
Grade 2	3	0.7%	3	0.7%	6	0.7%
**Haematuria**						
Not Graded	13	3.0%	10	2.4%	23	2.7%
Grade 0	416	96.3%	399	96.1%	815	96.2%
Grade 1	2	0.5%	5	1.2%	7	0.8%
Grade 2	1	0.2%	1	0.2%	2	0.2%
**Prostatic obstruction**						
Not Graded	428	99.1%	412	99.3%	840	99.2%
Grade 1	4	0.9%	3	0.7%	7	0.8%
**Urinary Frequency**						
Not Graded	12	2.8%	13	3.1%	25	3.0%
Grade 0	290	67.1%	257	61.9%	547	64.6%
Grade 1	117	27.1%	140	33.7%	257	30.3%
Grade 2	13	3.0%	5	1.2%	18	2.1%
**Urinary Incontinence**						
Not Graded	12	2.8%	16	3.9%	28	3.3%
Grade 0	381	88.2%	370	89.2%	751	88.7%
Grade 1	37	8.6%	24	5.8%	61	7.2%
Grade 2	1	0.2%	4	1.0%	5	0.6%
Grade 3	1	0.2%	1	0.2%	2	0.2%
**Urinary Retention**						
Not Graded	12	2.8%	13	3.1%	25	3.0%

Grade 0	384	88.9%	361	87.0%	745	88.0%
Grade 1	24	5.6%	22	5.3%	46	5.4%
Grade 2	12	2.8%	18	4.3%	30	3.5%
Grade 3	0	0.0%	1	0.2%	1	0.1%
**Urinary Urgency**						
Not Graded	12	2.8%	14	3.4%	26	3.1%
Grade 0	303	70.1%	301	72.5%	604	71.3%
Grade 1	112	25.9%	94	22.7%	206	24.3%
Grade 2	5	1.2%	6	1.4%	11	1.3%
**Total**	**432**	**100.0%**	**415**	**100.0%**	**847**	**100.0%**

Supplementary Figure 3. Mean average EPIC-26 subdomain scores in the acute toxicity setting, separated by delivered radiotherapy technique

The urinary bother question is graphed separately, as it does not form part of the urinary incontinence or obstructive subdomain scores. Error bars show 95% confidence interval for estimates of mean subdomain scores. Note that the time period between baseline scoring and week 4 post radiotherapy follow-up is variable, since the total time of radiotherapy delivery varied (SBRT in 1 or 2 weeks; CFMHRT in 4 or 7.8 weeks). Week 0 is the baseline toxicity score taken before start of radiotherapy.

Abbreviations: EPIC-26 = Expanded Prostate Cancer Index Composite (26 question); CFMHRT = Conventionally or Moderately Hypofractionated Radiotherapy; SBRT = Stereotactic Body Radiotherapy; BL = Baseline Pre-Radiotherapy.

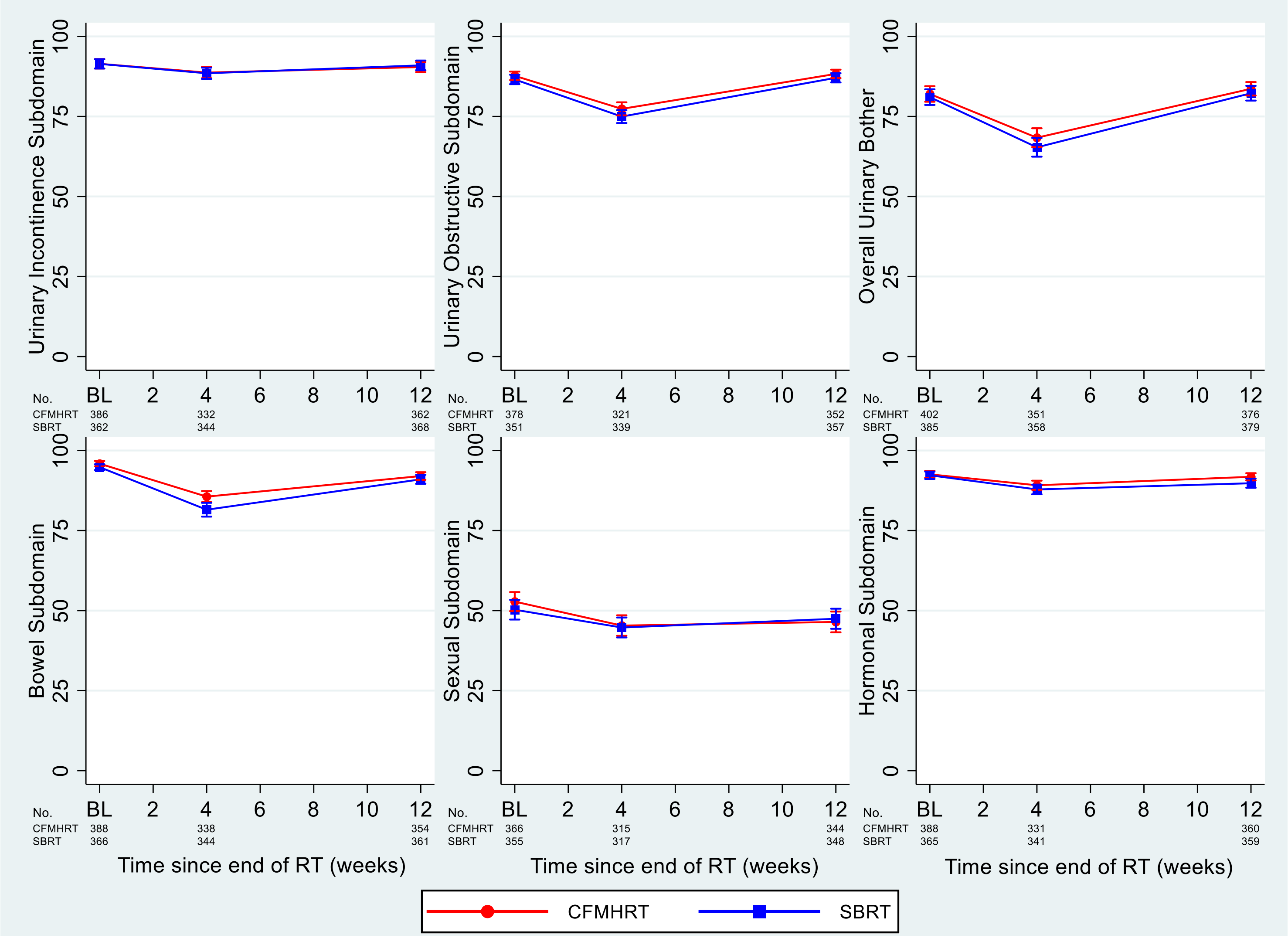

Supplementary Table 25. Comparison of Median Scores for EPIC-26 Subdomains

Abbreviations: EPIC-26 = Expanded Prostate Cancer Index Composite (26 question); CFMHRT = Conventionally or Moderately Hypofractionated Radiotherapy; SBRT = Stereotactic Body Radiotherapy; IQR = Interquartile range; RT = radiotherapy.

EPIC-26 Subdomain	Per Protocol Treatment						Mann-Whitney
	CFMHRT			SBRT			
	n	Median	IQR	n	Median	IQR	p-value
**Urinary Incontinence**							
Baseline	386	100	85.5 – 100	362	100	85.5 – 100	0.75
Worst	406	93.75	79.25 – 100	400	93.75	77.25 – 100	0.84
Worst Minus Baseline	368	0	- 8.375 – 0	355	0	- 8.25 – 0	0.91
12 weeks post RT	362	100	85.5 – 100	368	100	85.5 – 100	0.72
**Urinary Obstructive**							
Baseline	378	87.5	81.25 – 100	351	87.5	81.25 – 100	0.33
Worst	399	81.25	68.75 – 93.75	399	81.25	62.5 – 87.5	0.053
Worst Minus Baseline	354	-6.25	-18.75 – 0	342	-6.25	-18.75 – 0	0.50
12 weeks post RT	352	93.75	81.25 – 100	357	87.5	81.25 – 100	0.28
**Urinary Bother**							
Baseline	402	100	75 – 100	385	100	75 – 100	0.40
Worst	413	75	50 – 100	403	75	50 – 75	0.15
Worst Minus Baseline	390	0	-25 – 0	378	0	-25 – 0	0.32
12 weeks post RT	376	100	75 – 100	379	100	75 – 100	0.65
**Bowel**							
Baseline	388	100	95.8 – 100	366	100	91.7 – 100	0.014
Worst	404	91.7	75 – 100	400	87.5	75 – 95.8	0.024
Worst Minus Baseline	369	-4.2	-16.7 – 0	359	-8.3	-20.8 – 0	0.081
12 weeks post RT	354	95.8	87.5 – 100	361	95.8	87.5 – 100	0.61
**Sexual**							
Baseline	366	52.8	26.3 – 75	355	48.7	22.2 – 75	0.23
Worst	388	39.6	16.7 – 65.3	376	36.2	16.7 – 65.3	0.80
Worst Minus Baseline	342	-9.7	-25 – 0	333	-5.7	-21.1 – 1.3	0.081
12 weeks post RT	344	44.5	18 – 72.1	348	44.5	18 – 74	0.63
**Hormonal**							
Baseline	388	97.5	90 – 100	365	95	90 – 100	0.74
Worst	403	93.75	80 – 100	391	90	80 – 100	0.019
Worst Minus Baseline	370	0	-10 – 0	350	-5	-12.5 – 0	0.020
12 weeks post RT	360	95	85 – 100	359	95	85 – 100	0.11

Supplementary Table 26. EPIC-26 Score Reductions at Any Timepoint (up to 12 weeks post radiotherapy) Exceeding Minimal Clinically Important Differences

Per main manuscript, a clinically important point reduction in EPIC-26 subdomain score was defined separately by subdomain: urinary incontinence (8 point) urinary obstructive (6 point), bowel (5 point), sexual (11 point), hormonal (5 point). Data missing if either baseline and/or all follow-up data points missing.

Abbreviations: EPIC-26 = Expanded Prostate Cancer Index Composite (26 question); MCID = Minimal Clinically Important Difference; CFMHRT = Conventionally or Moderately Hypofractionated Radiotherapy; SBRT = Stereotactic Body Radiotherapy; RT = radiotherapy.

EPIC-26 MCID Reduction at Any Timepoint	Per Protocol Treatment				Comparison
	CFMHRT		SBRT		
	n	%	n	%	
**Urinary Incontinence**					
No	255	69.3%	255	71.8%	Difference −2.5%<br>95% CI −9.2 to 4.1%<br>p=0.45
Yes	113	30.7%	100	28.2%	
Missing Data	64	N/A	60	N/A	
**Urinary Obstructive**					
No	137	38.7%	129	37.7%	Difference 1.0%<br>95% CI −6.2 to 8.2%<br>p=0.79
Yes	217	61.3%	213	62.3%	
Missing Data	78	N/A	73	N/A	
**Bowel**					
No	189	51.2%	161	44.8%	Difference 6.4%<br>95% CI −0.9 to 13.6%<br>p=0.085
Yes	180	48.8%	198	55.2%	
Missing Data	63	N/A	56	N/A	
**Sexual**					
No	174	50.9%	194	58.3%	Difference −7.4%<br>95% CI −14.9 to 0.1%<br>p=0.054
Yes	168	49.1%	139	41.7%	
Missing Data	90	N/A	82	N/A	
**Hormonal**					
No	198	53.5%	162	46.3%	Difference 7.2%<br>95% CI −0.06 to 14.5%<br>p=0.053
Yes	172	46.5%	188	53.7%	
Missing Data	61	N/A	65	N/A	

Supplementary Table 27. EPIC-26 Score Reductions at 12 Weeks post Radiotherapy Exceeding Minimal Clinically Important Differences

Per main manuscript, a clinically important point reduction in EPIC-26 subdomain score was defined separately by subdomain: urinary incontinence (8 point) urinary obstructive (6 point), bowel (5 point), sexual (11 point), hormonal (5 point). Data missing if either baseline and/or week 12 post radiotherapy follow-up data points missing.

Abbreviations: EPIC-26 = Expanded Prostate Cancer Index Composite (26 question); MCID = Minimal Clinically Important Difference; CFMHRT = Conventionally or Moderately Hypofractionated Radiotherapy; SBRT = Stereotactic Body Radiotherapy; IQR = Interquartile range; RT = radiotherapy.

EPIC-26 MCID Reduction at Week 12	Per Protocol Treatment				Comparison
	CFMHRT		SBRT		
	n	%	n	%	
**Urinary Incontinence**					
No	261	79.3%	275	84.1%	Difference −4.8%<br>95% CI −10.7 to 1.1%<br>p=0.11
Yes	68	20.7%	52	15.9%	
Missing Data	103	N/A	88	N/A	
**Urinary Obstructive**					
No	206	65.6%	204	65.8%	Difference −0.2%<br>95% CI −7.7 to 7.2%<br>p=0.96
Yes	108	34.4%	106	34.2%	
Missing Data	118	N/A	105	N/A	
**Bowel**					
No	233	71.7%	235	71.9%	Difference −0.2%<br>95% CI −7.1 to 6.7%<br>p=0.96
Yes	92	28.3%	92	28.1%	
Missing Data	107	N/A	88	N/A	
**Sexual**					
No	187	61.1%	216	69.5%	Difference −8.3%<br>95% CI −15.8 to 0.9%<br>p=0.029
Yes	119	38.9%	95	30.5%	
Missing Data	126	N/A	104	N/A	
**Hormonal**					
No	227	68.6%	199	60.7%	Difference 7.9%<br>95% CI 0.6 to 15.2%<br>p=0.034
Yes	104	31.4%	129	39.3%	
Missing Data	101	N/A	87	N/A	

Supplementary Figure 4. Change from Baseline IPSS Scores

Changes from baseline IPSS scores, by time, for CFMHRT and SBRT. Patients included at any timepoint if both baseline and relevant timepoint score available. The IPSS total is formed by the sum of all subscores except for urinary QoL. Note that the time period between baseline scoring and week 2 post radiotherapy follow-up is variable, since the total time of radiotherapy delivery varied (SBRT in 1 or 2 weeks; CFMHRT in 4 or 7.8 weeks). 95% confidence intervals for each point mean estimate are displayed. Week 0 is the baseline toxicity score taken before start of radiotherapy. Abbreviations: IPSS = International Prostate Symptom Score; QoL = Quality of Life; CFMHRT = Conventionally or Moderately Hypofractionated Radiotherapy; SBRT = Stereotactic Body Radiotherapy; BL = Baseline Pre-Radiotherapy; RT = Radiotherapy.

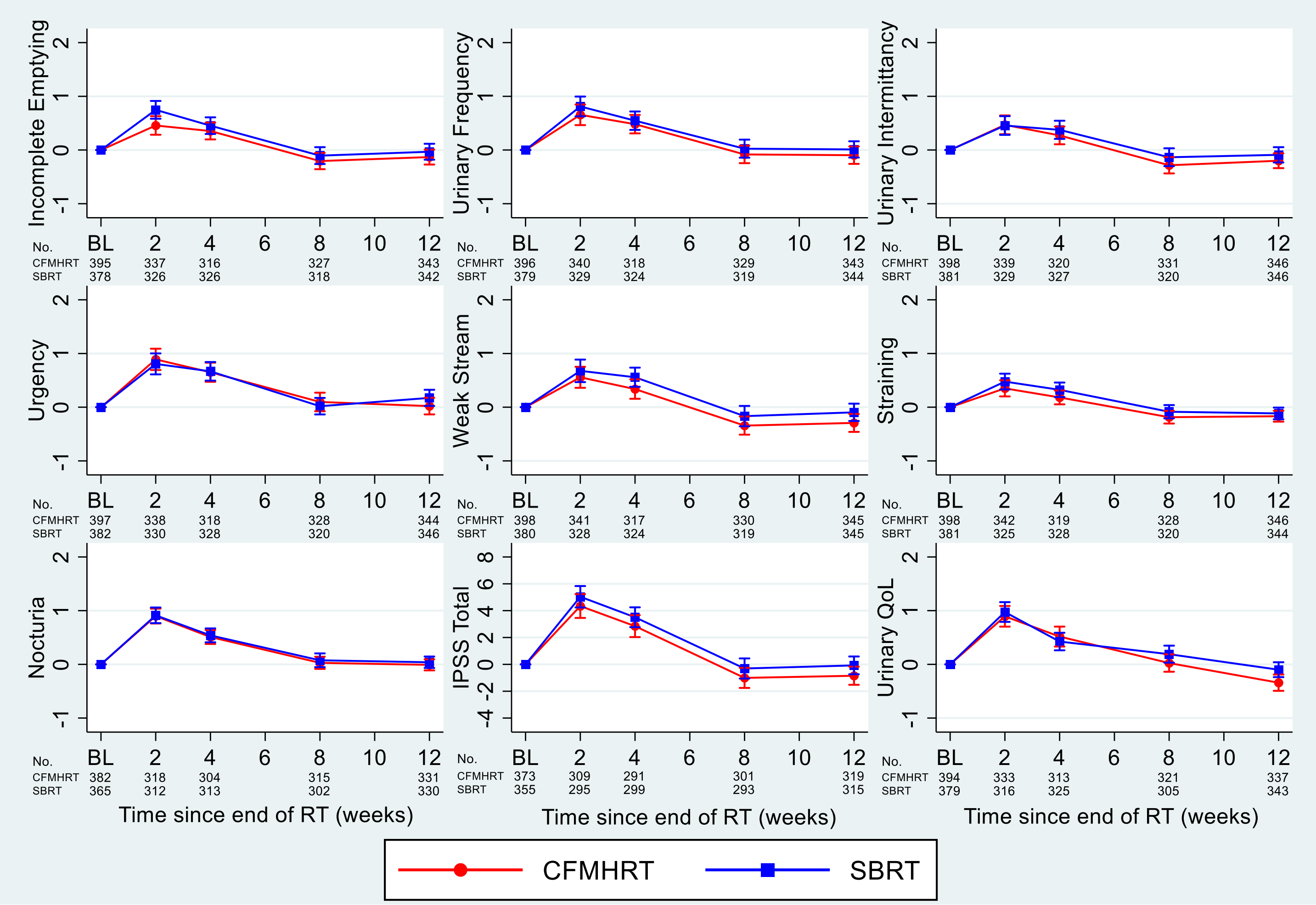

Supplementary Figure 5. Average IPSS Subscores, Total & Quality of Life

Averages for IPSS subscores, total and quality of life score, by time, for CFMHRT and SBRT. The IPSS total is formed by the sum of all subscores except for urinary QoL. Note that the time period between baseline scoring and week 4 post radiotherapy follow-up is variable, since the total time of radiotherapy delivery varied (SBRT in 1 or 2 weeks; CFMHRT in 4 or 7.8 weeks). 95% confidence intervals for each point mean estimate are displayed. Week 0 is the baseline toxicity score taken before start of radiotherapy.

Abbreviations: IPSS = International Prostate Symptom Score; QoL = Quality of Life; CFMHRT = Conventionally or Moderately Hypofractionated Radiotherapy; SBRT = Stereotactic Body Radiotherapy; BL = Baseline Pre-Radiotherapy.

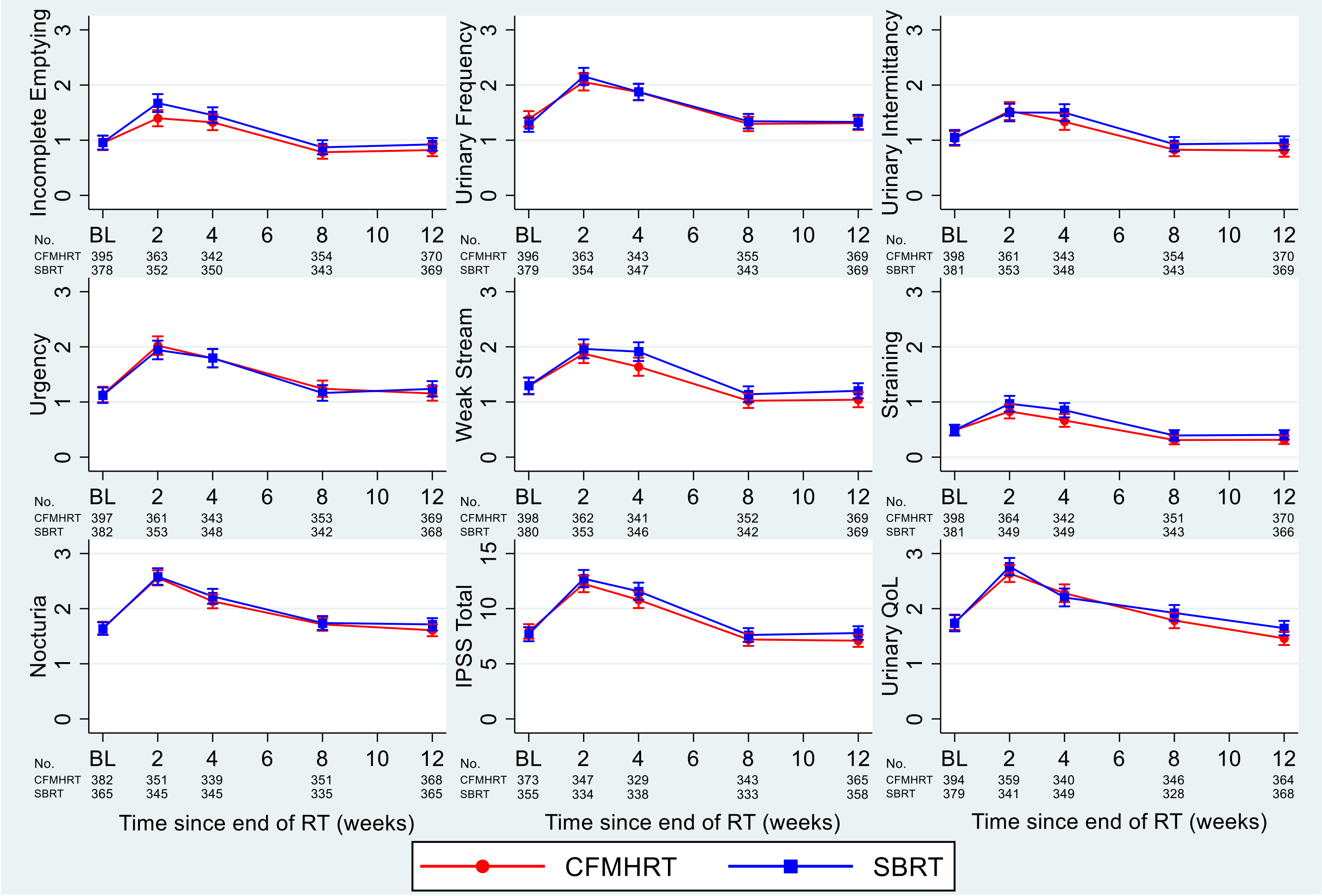

Supplementary Table 28. Comparison of Median IPSS Total Scores and Quality of Life Scores

Abbreviations: IPSS = International Prostate Symptom Score; CFMHRT = Conventionally or Moderately Hypofractionated Radiotherapy; SBRT = Stereotactic Body Radiotherapy; IQR = Interquartile range; RT = radiotherapy.

IPSS Parameter	Per Protocol Treatment						Mann-Whitney
	CFMHRT			SBRT			
	n	Median	IQR	n	Median	IQR	p-value
**IPSS Total Score**							
Baseline	373	6	3 – 11	355	6	3 – 12	0.70
Worst	420	13	7 – 19	402	13	8 – 19	0.076
Worst Minus Baseline	365	5	1 – 10	348	6	2 – 10	0.035
12 weeks post RT	365	6	3 – 10	358	6.5	3 – 11	0.13
**IPSS QoL Score**							
Baseline	394	2	1 – 3	379	2	1 – 3	0.74
Worst	423	3	2 – 4	409	3	2 – 4	0.41
Worst Minus Baseline	387	1	0 – 2	376	1	0 – 2	0.20
12 week post RT	364	1	1 – 2	368	2	1 – 2	0.044

Supplementary Figure 6. IPSS Severity Categories Over Time

Changes in IPSS severity categories after radiotherapy. Categories are defined by the IPSS total score; none (score 0), mild (score 1-7), moderate (score 8-19), severe (score 20-35). Note that the time period between baseline scoring and week 4 post radiotherapy follow-up is variable, since the total time of radiotherapy delivery varied (SBRT in 1 or 2 weeks; CFMHRT in 4 or 7.8 weeks). Week 0 is the baseline toxicity score taken before start of radiotherapy.

Abbreviations: IPSS = International Prostate Symptom Score; CFMHRT = Conventionally or Moderately Hypofractionated Radiotherapy; SBRT = Stereotactic Body Radiotherapy; BL = Baseline Pre-Radiotherapy.

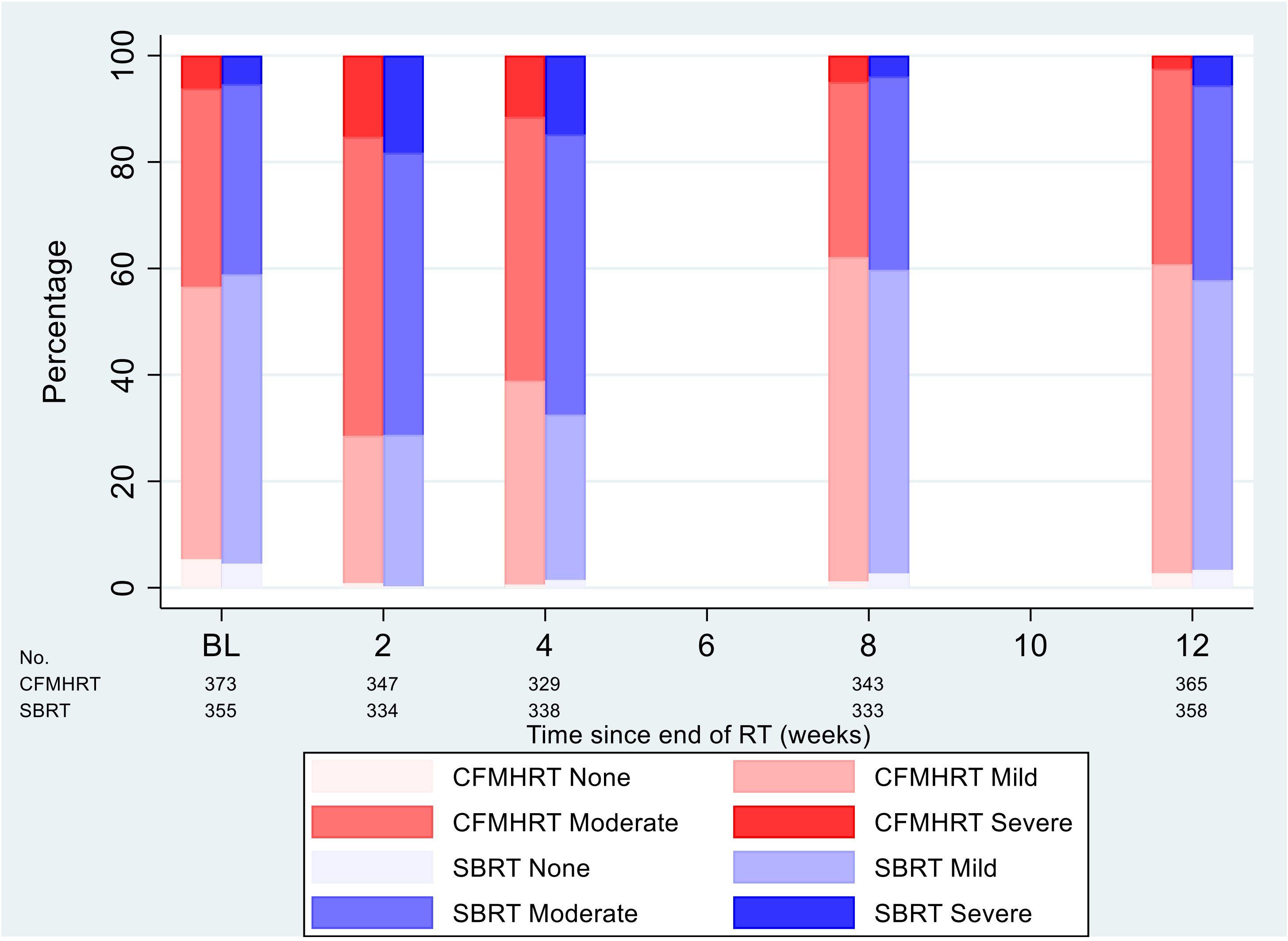

Supplementary Table 29. Comparison of IPSS Total Score Categories

Abbreviations: IPSS = International Prostate Symptom Score; CFMHRT = Conventionally or Moderately Hypofractionated Radiotherapy; SBRT = Stereotactic Body Radiotherapy; IQR = Interquartile range; RT = radiotherapy.

IPSS Total Score Categories at Timepoint	Per Protocol Treatment				Chi-Square / Fishers
	CFMHRT		SBRT		
	n	%	n	%	p-value
**Baseline**					
None	20	5.4%	16	4.5%	0.82 (Chi-square)
Mild (1-7)	191	51.2%	193	54.4%	
Moderate (8-19)	139	37.3%	127	35.8%	
Severe (20-35)	23	6.2%	19	5.4%	
**Worst**					
None	1	0.2%	1	0.2%	0.15 (Fisher's)
Mild (1-7)	107	25.5%	80	19.9%	
Moderate (8-19)	232	55.2%	227	56.5%	
Severe (20-35)	80	19.0%	94	23.4%	
**Week 12 Post RT**					
None	10	2.7%	12	3.4%	0.17 (Chi-square)
Mild (1-7)	212	58.1%	195	54.5%	
Moderate (8-19)	134	36.7%	131	36.6%	
Severe (20-35)	9	2.5%	20	5.6%	

Supplementary Table 30. Comparison of Median IIEF-5 Scores

Abbreviations: IIEF-5 = International Index of Erectile Function (5 questions); CFMHRT = Conventionally or Moderately Hypofractionated Radiotherapy; SBRT = Stereotactic Body Radiotherapy; IQR = Interquartile range; RT = radiotherapy.

IIEF – 5 Scores	Per Protocol Treatment						Mann-Whitney
	CFMHRT			SBRT			
	n	Median	IQR	n	Median	IQR	p-value
**Baseline**	322	16	7 – 21	309	14	7 – 20	0.13
**Week 12**	280	12	5 – 20	286	12.5	5 – 20	0.86

Supplementary Figure 7. Change from Baseline Vaizey Total Scores

Changes from baseline Vaizey total scores, by time, for CFMHRT and SBRT. Patients included at any timepoint if both baseline and relevant timepoint score available. Note that the time period between baseline scoring and week 4 post radiotherapy follow-up is variable, since the total time of radiotherapy delivery varied (SBRT in 1 or 2 weeks; CFMHRT in 4 or 7.8 weeks). 95% confidence intervals for each point mean estimate are displayed. Week 0 is the baseline toxicity score taken before start of radiotherapy. N.B. Higher score for Vaizey is worse: 0 = perfect continence; maximum score = 24 = totally incontinent.

Abbreviations: Conventionally or Moderately Hypofractionated Radiotherapy; SBRT = Stereotactic Body Radiotherapy; BL = Baseline Pre-Radiotherapy.

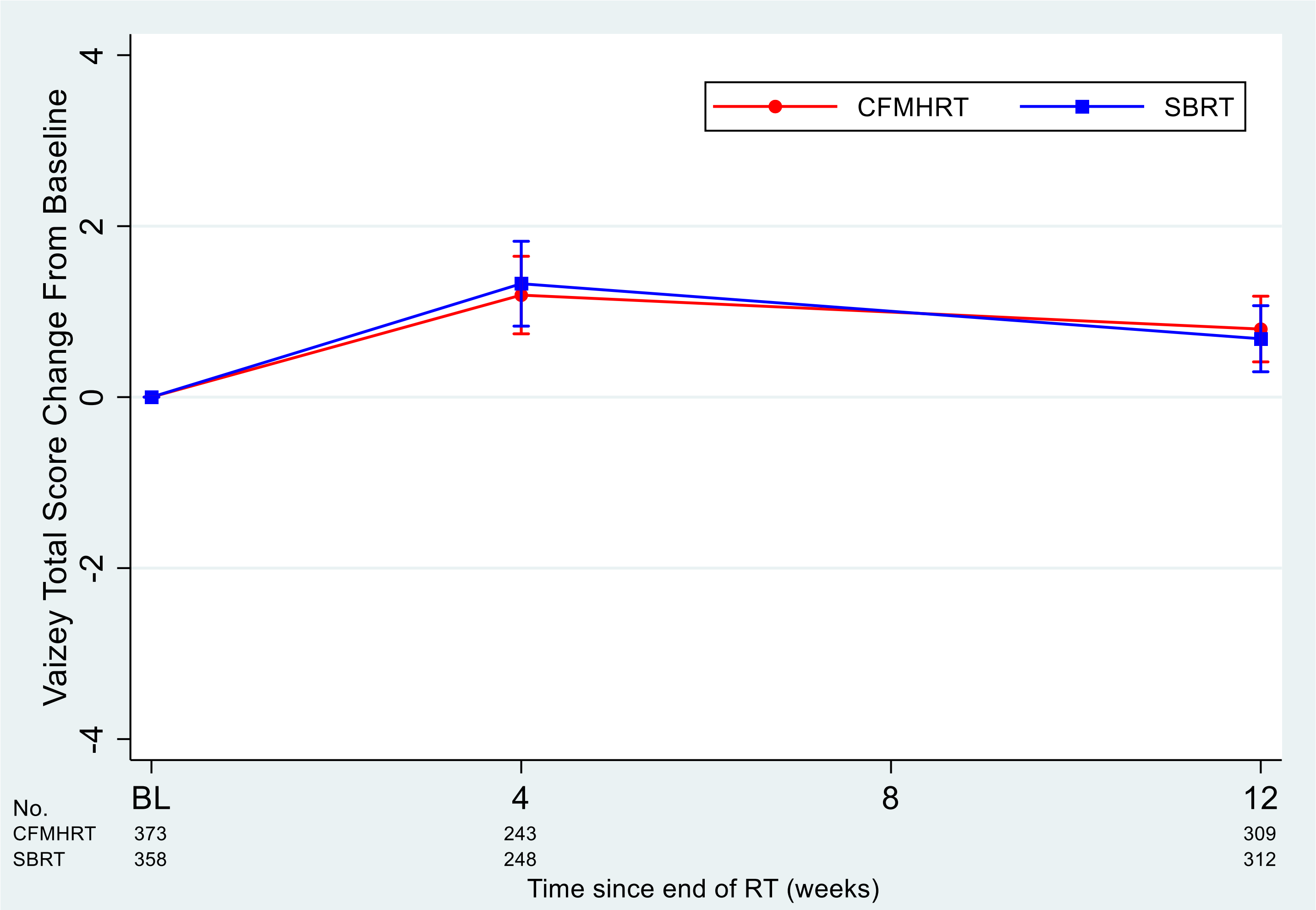

Supplementary Figure 8. Vaizey Total Scores Between Baseline and Week 12 Post Radiotherapy

Averages for Vaizey total scores, at baseline and week 12 post radiotherapy, for CFMHRT and SBRT. Note that the time period between baseline scoring and week 12 post radiotherapy follow-up is variable, since the total time of radiotherapy delivery varied (SBRT in 1 or 2 weeks; CFMHRT in 4 or 7.8 weeks). 95% confidence intervals for each point mean estimate are displayed. Week 0 is the baseline toxicity score taken before start of radiotherapy. N.B. Higher score for Vaizey is worse: 0 = perfect continence; maximum score = 24 = totally incontinent.

Abbreviations: CFMHRT = Conventionally or Moderately Hypofractionated Radiotherapy; SBRT = Stereotactic Body Radiotherapy; BL = Baseline Pre-Radiotherapy.

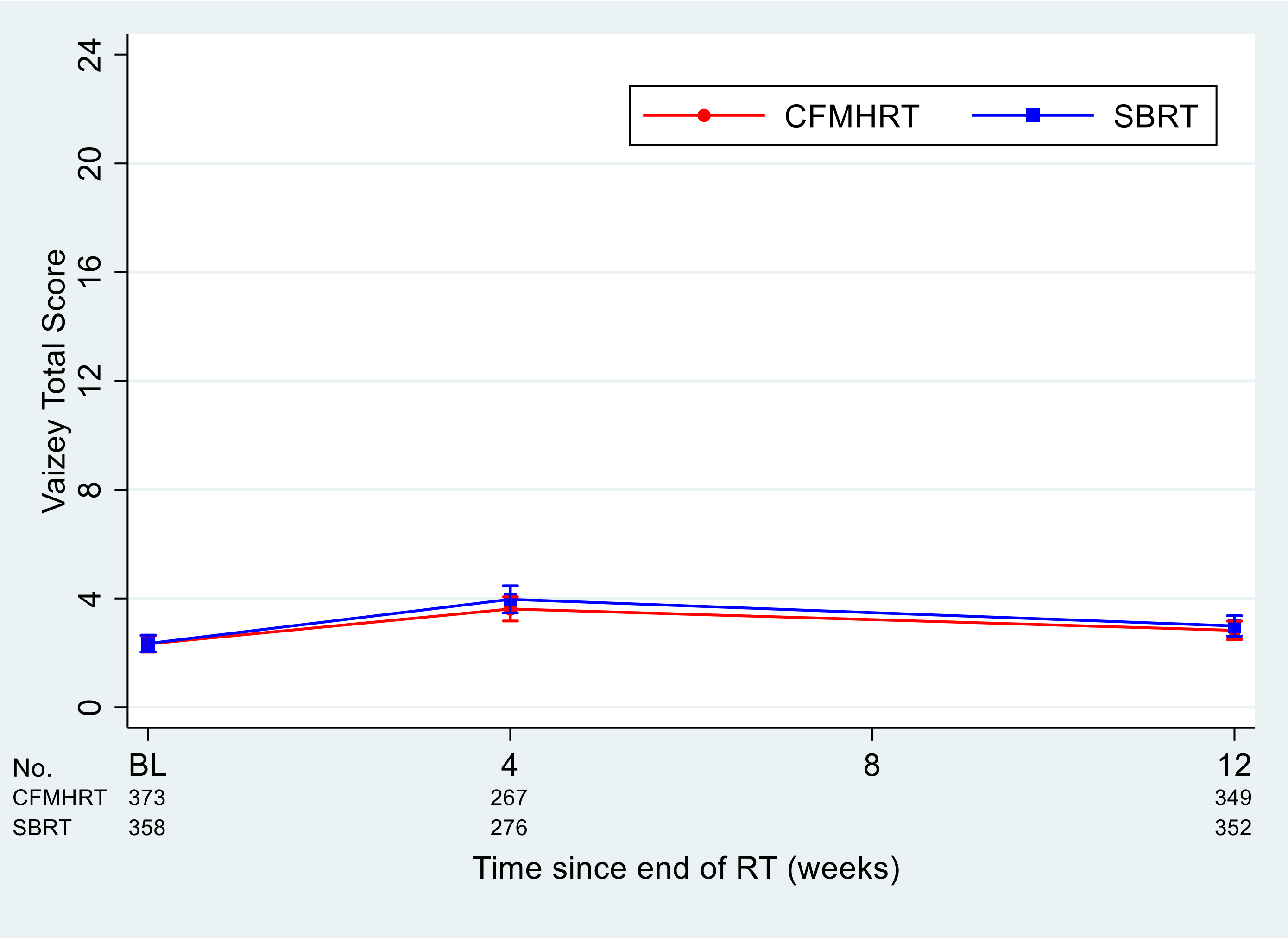

Supplementary Table 31. Comparison of Median Vaizey Total Scores

Abbreviations: Conventionally or Moderately Hypofractionated Radiotherapy; SBRT = Stereotactic Body Radiotherapy; IQR = Interquartile range; RT = radiotherapy.

Vaizey Scores	Per Protocol Treatment						Mann-Whitney
	CFMHRT			SBRT			
	n	Median	IQR	n	Median	IQR	p-value
**Baseline**	373	1	0 – 4	358	1	0 – 4	0.99
**Worst**	384	4	1 – 6	381	4	0 – 6	0.82
**Worst Change cf. Baseline**	214	2	0 – 4	223	1	0 – 4	0.84
**Week 12 Post RT**	349	2	0 – 4	352	2	0 – 4	0.75

<u>Supplementary Figure 9. RTOG Gastrointestinal Acute Toxicity (For SBRT Patients Only) By Delivery Platform</u>

RTOG acute gastrointestinal toxicity presented only for patients receiving SBRT, separated into those receiving CyberKnife and those receiving non-CyberKnife radiotherapy. Because the trial allowed two different treatment durations (1 or 2 weeks) it was necessary to interpolate data where assessments did not overlap, as described in **Supplementary Appendix 2**. X-axis scale matched to other RTOG graphs to facilitate comparison. Week 0 is the baseline toxicity score taken before start of radiotherapy. Abbreviations: RTOG = Radiation Therapy Oncology Group; RT = Radiotherapy.

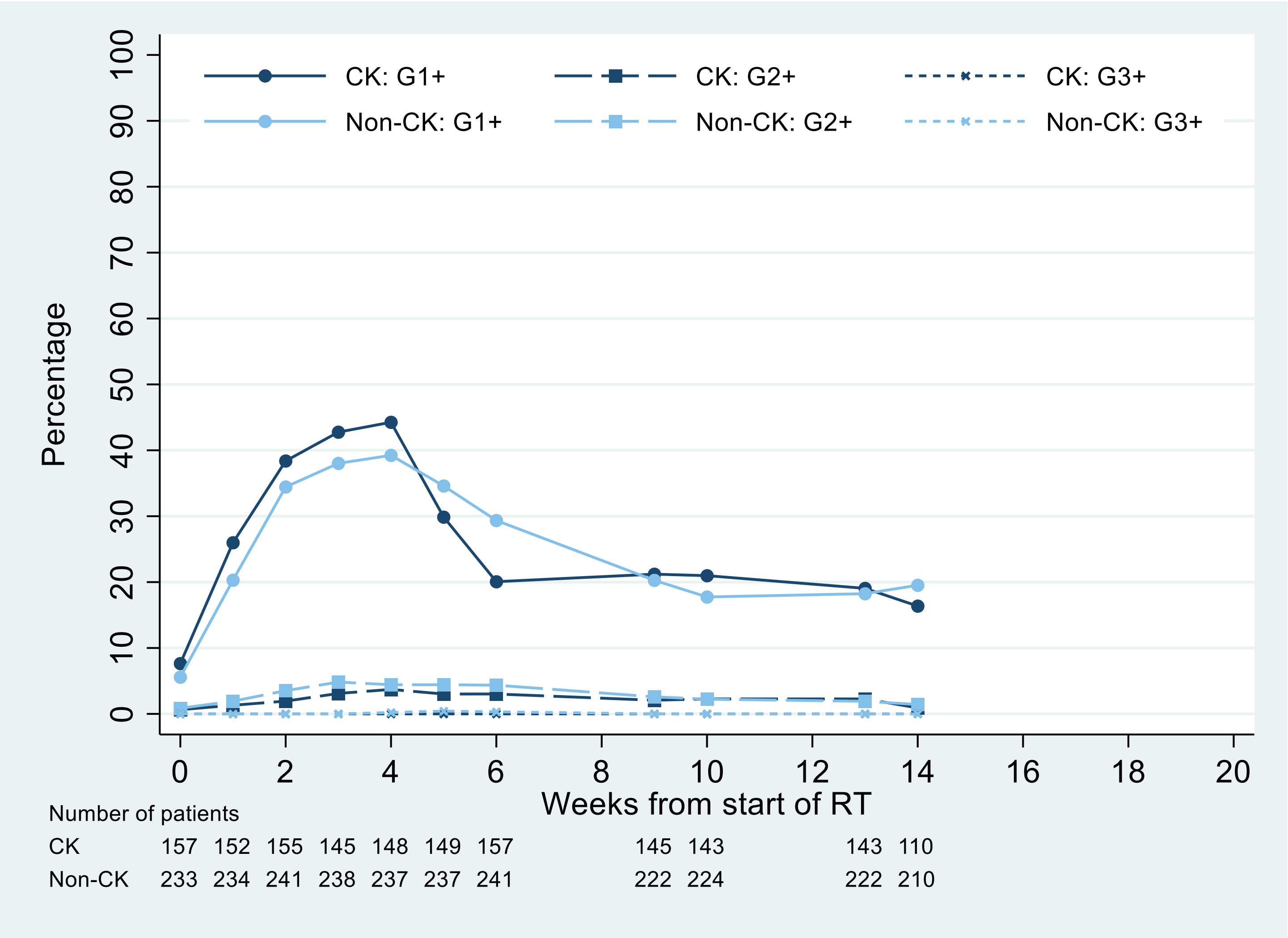

Supplementary Figure 10. RTOG Genitourinary Acute Toxicity (For SBRT Patients Only) By Delivery Platform

RTOG acute genitourinary toxicity presented only for patients receiving SBRT, separated into those receiving CyberKnife and those receiving non-CyberKnife radiotherapy. Because the trial allowed two different treatment durations (1 or 2 weeks) it was necessary to interpolate data where assessments did not overlap, as described in **Supplementary Appendix 2**. X-axis scale matched to other RTOG graphs to facilitate comparison. Week 0 is the baseline toxicity score taken before start of radiotherapy. Numbers at risk for each arm are asynchronous because they are shown only at data collection timepoints (which are non-simultaneous relative to the start of radiotherapy. Abbreviations: RTOG = Radiation Therapy Oncology Group; RT = Radiotherapy.

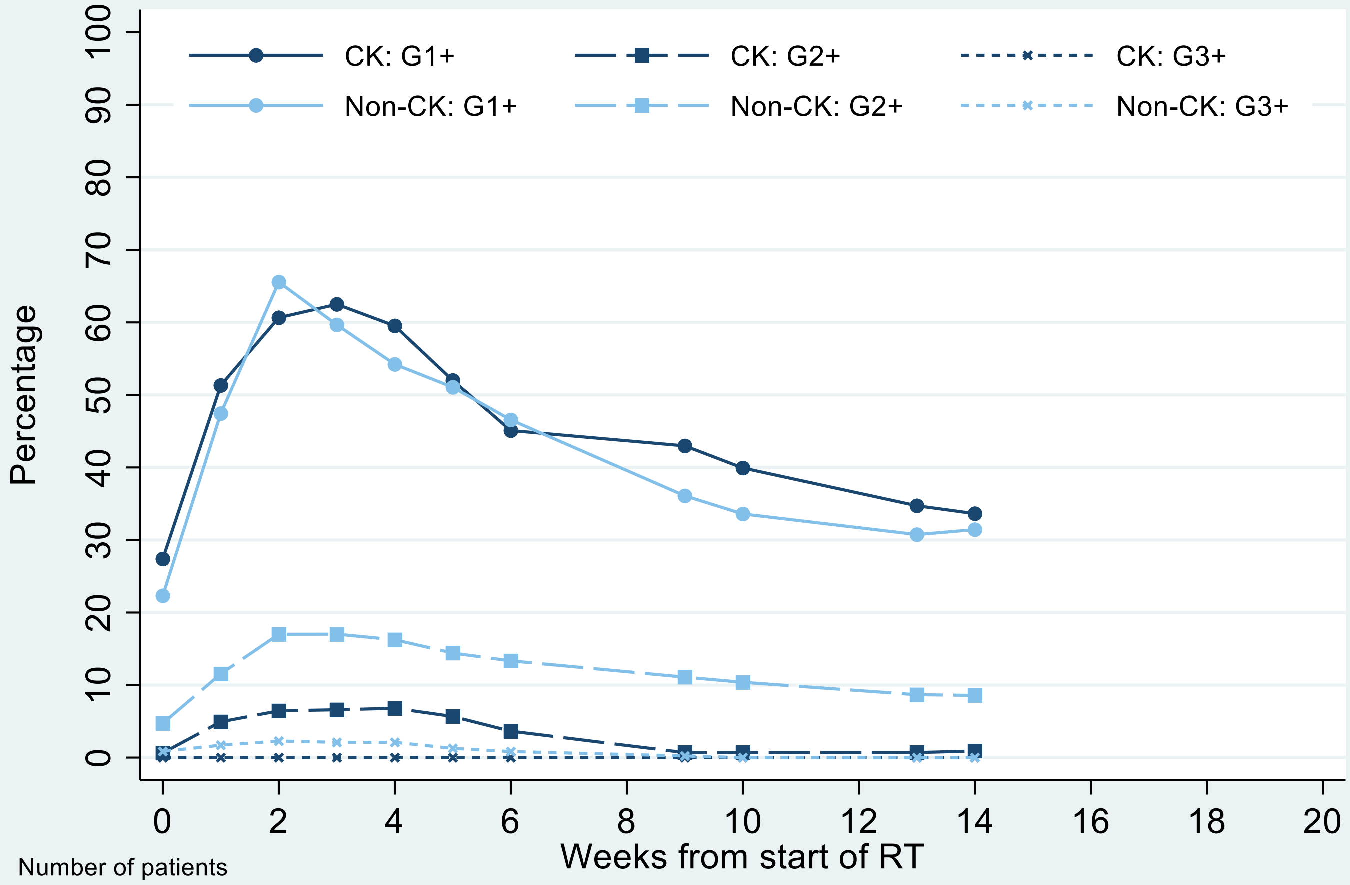

Number of patients

CK	157	152	155	144	147	150	158	146	144	144	110
Non-CK	233	234	241	238	237	236	240	221	224	222	210

Supplementary Figure 11. RTOG Gastrointestinal Acute Toxicity (For CFMHRT Patients Only) By Treating Centre CyberKnife Status

RTOG acute gastrointestinal toxicity presented only for patients receiving CFMHRT, separated into those receiving radiotherapy at a centre which performed their SBRT treatments on CyberKnife versus non-CyberKnife platforms. Because the trial allowed two different treatment durations (1 or 2 weeks) it was necessary to interpolate data where assessments did not overlap, as described in **Supplementary Appendix 2**. Week 0 is the baseline toxicity score taken before start of radiotherapy. Numbers at risk for each arm are asynchronous because they are shown only at data collection timepoints (which are non-simultaneous relative to the start of radiotherapy. Abbreviations: RTOG = Radiation Therapy Oncology Group; RT = Radiotherapy; CK = CyberKnife.

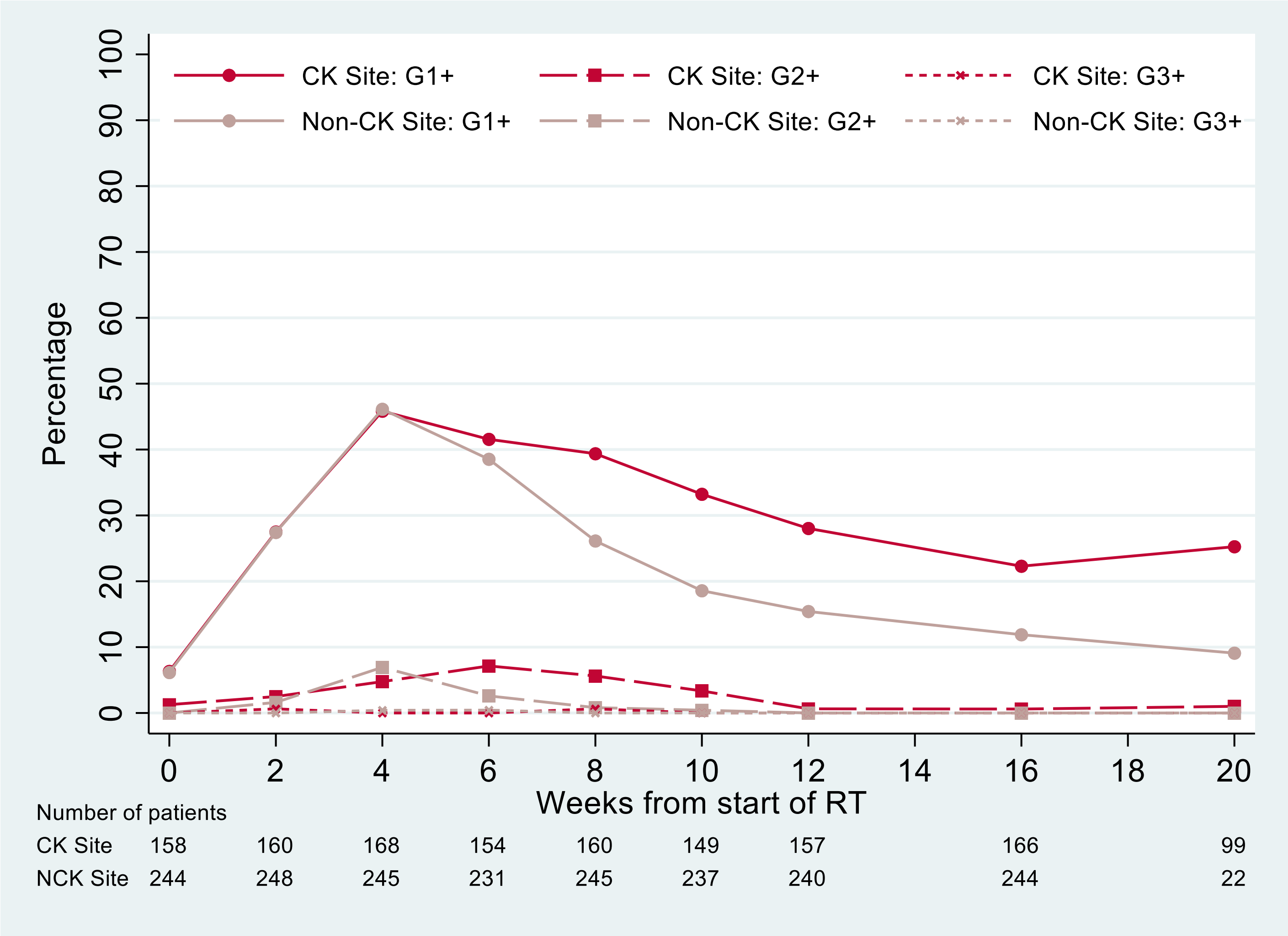

Supplementary Figure 12. RTOG Genitourinary Acute Toxicity (For CFMHRT Patients Only) By Treating Centre CyberKnife Status

RTOG acute genitourinary toxicity presented only for patients receiving CFMHRT, separated into those receiving radiotherapy at a centre which performed their SBRT treatments on CyberKnife versus non-CyberKnife platforms. Because the trial allowed two different treatment durations (1 or 2 weeks) it was necessary to interpolate data where assessments did not overlap, as described in **Supplementary Appendix 2**. Week 0 is the baseline toxicity score taken before start of radiotherapy. Numbers at risk for each arm are asynchronous because they are shown only at data collection timepoints (which are non-simultaneous relative to the start of radiotherapy. Abbreviations: RTOG = Radiation Therapy Oncology Group; RT = Radiotherapy; CK = CyberKnife.

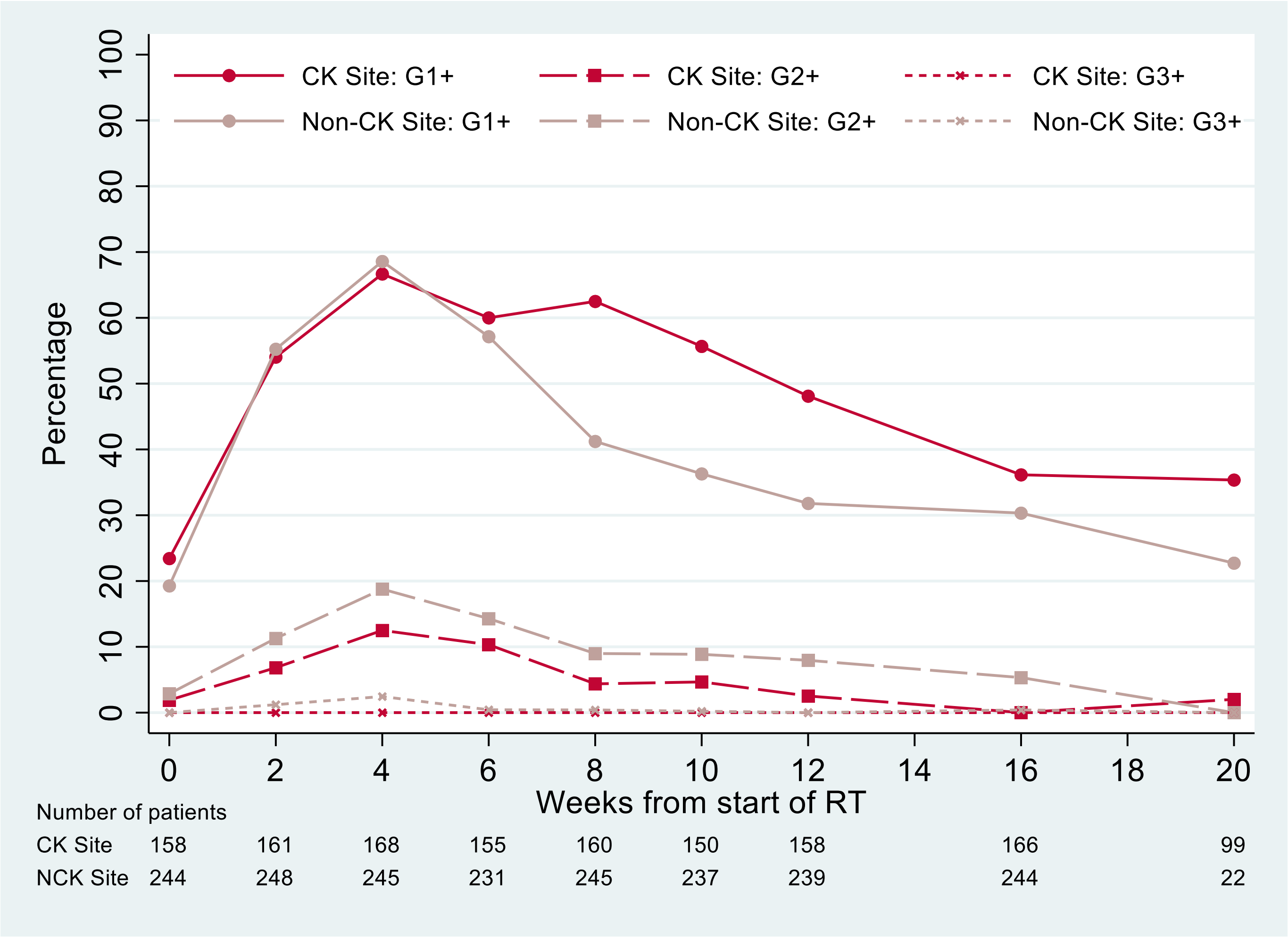

Supplementary Table 32. PACE RTOG Toxicity with Reference to Comparable Hypofractionation Trials

Trial and Fractionation	RTOG G2+ Acute Toxicity	
	GI (%)	GU (%)
**PACE**		
78 Gy in 39f **OR**<br>62 Gy in 20f	12.3	27.3
36.25 Gy in 5f	10.4	23.1
**CHHiP**		
74 Gy / 37f	25	46
60 Gy / 20f	38	49
57 Gy / 19f	38	46
**PROFIT**		
78 Gy / 39f	10.5	27.4
60 Gy / 20f	16.7	30.9

Supplementary Table 33. Reported Toxicity After SBRT to the Prostate in Low-Intermediate Risk Patients

Trial	C.	n	Risk	ADT	CTV	Margins	Plan Extras	Machine/IGRT	PTV Dose	Fr	Frequency	Score	Acute GI		Acute GU	
						(mm)			(Gy)				G2 (%)	G3/4 (%)	G2 (%)	G3/4 (%)
Widmark [1]*<br>ISRCTN45905321	12	589	IR-HR	No	Pros. Only	7	MRI	LINAC<br>80% 3DCRT<br>20% IMRT<br>BeamCath/fidx	42.7	7	2.5 weeks	RTOG	7.5	1	22	6
**PACE<br>SBRT Arm**	**37**	**415**	**LR-IR**	**No**	**LR - Pros. Only<br>IR - Pros. + 1cm SV**	**Mostly<br>≤ 5mm**	**± MRI**	**CK / LINAC<br>Various IGRT**	**36.25**	**5**	**Either daily or<br>alt days**	**RTOG<br>CTCAE**	**10.4<br>14.9**	**0.2<br>0.7**	**20.7<br>29.2**	**2.4<br>1.7**
Meier [2]<br>NCT00643994	21	309	LR-IR	No	Pros. Only	5 (3 Post.)	MRI	CK<br>fidx + Intra-kV	36.25	5	Daily/Alt Days	CTCAE	8.1	0	26	0
Katz [3] †	1	304	LR-HR	18.8%	LR - Pros. Only<br>IR - Pros. + Prox SV	3-5<br>Some 8	± MRI	CK<br>fidx + Intra-kV	35 (n=50)<br>36.25 (n=254)	5	Daily	RTOG	4<br>3.6	0<br>0	4<br>4.7	0<br>0
Fuller [4]<br>NCT00643617	7	259	LR-IR	No	LR - Pros. Only<br>IR - Pros. + 1cm SV	2 (0 Post.)	Foley-CT<br>± MRI	CK<br>fidx + Intra-kV	38	4	Daily	CTCAE	6.9	0	35.1	1.1
Quon [5]<br>NCT01423474	3	152	LR-Low IR	<5%	Pros. Only	0·3	No	LINAC IMRT<br>fidx + kV/CBCT	38	5	Alt Days (Arm 1)<br>Weekly (Arm 2)	RTOG	18·4<br>10·8	0<br>0	31·6<br>33·8	1.3<br>2.7
Zelefsky [6]	1	136	LR-IR	No	Pros. + SV	5 (3 Post.)	No	LINAC IMRT<br>Calypso or fidx	32.5-40	5	Alt Days	CTCAE	4.4	0	16.2	0
Mantz [7]	1	102	LR	No	Pros. Only	2	No	LINAC IMRT<br>Calpyso	40	5	Alt Days	CTCAE	?	?	?	2 Pts
Hannan [8]	5	91	LR-Low IR	16.5%	Pros. Only	3	± MRI	LINAC IMRT<br>Calypso or fidx	45-50	5	Alt Days	CTCAE	17	0	20	0
Loblaw [9]<br>NCT01578902	1	84	LR	1%	Pros. Only	4	No	LINAC IMRT<br>fidx + kV	33.25	5	Weekly	CTCAE	10	0	19	1
Jackson [10]<br>NCT01288534	5	66	LR-Low IR	No	Pros. Only	3	MRI or Foley-CT	LINAC IMRT<br>Calypso	37	5	Every 3 Days	CTCAE	4	0	23	0
Boyer [11]<br>NCT00941915	3	60	LR-Low IR	No	Pros. Only	5 (3 Post.)	MRI	LINAC IMRT<br>Various IGRT	37	5	Alt Days	CTCAE	5	0	25	0
McBride [12]	4	45	LR	No	Pros. Only	5 (3 Post.)	± MRI<br>± Foley-CT	CK<br>fidx + Intra-kV	36.25-37.5	5	Max 10 days	CTCAE	7	0	19	2.2
Bolzicco [13]	1	45	LR-IR	37%	Pros. Only	5 (3 Post.)	Catheter	CK<br>fidx + Intra-kV	35	5	Daily	RTOG	24.4	0	11.1	0
Alongi [14]	NS	42	LR-IR	Some	NS	NS	NS	LINAC IMRT<br>CBCT±fidx	35 (LR)<br>37.5 (IR)	5	Daily	CTCAE	5	0	13	0
Alongi [15]	NS	40	LR-IR	Some	LR - Pros. Only<br>IR - Pros. + 1/3 SV	3-5	MRI	LINAC IMRT<br>CBCT±fidx	35	5	Alt Days	CTCAE	10	0	40	0
Madsen [16]	1	40	LR	NS	Pros. Only	4-5	MRI	3DCRT<br>fidx + kV	33.5	5	Daily	CTCAE	13	0	20.5	2.5

* Grade percentages estimated from figures in paper
† More recent re-analysis not included due to less information on acute toxicity data

Abbreviations: NS = Not Stated; SBRT = Stereotactic Body Radiotherapy; C. = centres; n = number of patients; ADT = androgen deprivation therapy; IGRT = image guided radiotherapy; Fr = fractions; GI = gastrointestinal; GU = genitourinary; GX = grade X; LR = low risk; IR = intermediate risk; HR = high risk; Pros. = prostate; SV = Seminal Vesicles; Post. = Posterior; CT = Computerised Tomography; Foley-CT = CT with Foley Catheter in-situ; MRI = magnetic resonance imaging; CK = CyberKnife; fidx = fiducials; kV = kilovoltage planar film; Intra-kV = intra-fractional kV; LINAC = linear accelerator; IMRT = intensity modulated radiotherapy; CBCT = cone beam CT; 3DCRT = 3-dimensional conformal radiotherapy; Alt days = alternate days; CTCAE = Common Terminology Criteria for Adverse Events; RTOG = Radiation Therapy Oncology Group.

Bibliography for Supplementary Table 33

1 Widmark A, Gunnlaugsson A, Beckman L, *et al.* Ultra-hypofractionated versus conventionally fractionated radiotherapy for prostate cancer: 5-year outcomes of the HYPO-RT-PC randomised, non-inferiority, phase 3 trial. *Lancet* 2019; **0**. DOI:10.1016/S0140-6736(19)31131-6.

2 Meier RM, Bloch DA, Cotrutz C, *et al.* Multicenter Trial of Stereotactic Body Radiation Therapy for Low- and Intermediate-Risk Prostate Cancer: Survival and Toxicity Endpoints. *Int J Radiat Oncol Biol Phys* 2018; **102**: 296–303.

3 Katz AJ, Santoro M, Diblasio F, Ashley R. Stereotactic body radiotherapy for localized prostate cancer: disease control and quality of life at 6 years. *Radiat Oncol* 2013; **8**: 118.

4 Fuller DB, Falchook AD, Crabtree T, *et al.* Phase 2 Multicenter Trial of Heterogeneous-dosing Stereotactic Body Radiotherapy for Low- and Intermediate-risk Prostate Cancer: 5-year Outcomes. *Eur Urol Oncol* 2018; **1**: 540–7.

5 Quon HC, Ong A, Cheung P, *et al.* Once-weekly versus every-other-day stereotactic body radiotherapy in patients with prostate cancer (PATRIOT): A phase 2 randomized trial. *Radiother Oncol* 2018; **127**: 206–12.

6 Zelefsky MJ, Kollmeier M, McBride S, *et al.* 5-Year Outcomes of a Phase I Dose Escalation Study Using Stereotactic Body Radiosurgery for Patients with Low and Intermediate Risk Prostate Cancer. *Int J Radiat Oncol* 2019; published online Jan 4. DOI:10.1016/J.IJROBP.2018.12.045.

7 Mantz C. A Phase II Trial of Stereotactic Ablative Body Radiotherapy for Low-Risk Prostate Cancer Using a Non-Robotic Linear Accelerator and Real-Time Target Tracking: Report of Toxicity, Quality of Life, and Disease Control Outcomes with 5-Year Minimum Follow-Up. *Front Oncol* 2014; **4**: 279.

8 Hannan R, Tumati V, Xie XJ, *et al.* Stereotactic body radiation therapy for low and intermediate risk prostate cancer - Results from a multi-institutional clinical trial. *Eur J Cancer* 2016; **59**: 142–51.

9 Loblaw A, Cheung P, D'Alimonte L, *et al.* Prostate stereotactic ablative body radiotherapy using a standard linear accelerator: Toxicity, biochemical, and pathological outcomes. *Radiother Oncol* 2013; **107**: 153–8.

10 Jackson WC, Dess RT, Litzenberg DW, *et al.* A multi-institutional phase 2 trial of prostate stereotactic body radiation therapy (SBRT) using continuous real-time evaluation of prostate motion with patient-reported quality of life. *Pract Radiat Oncol* 2018; **8**: 40–7.

11 Boyer MJ, Papagikos MA, Kiteley R, Vujaskovic Z, Wu J, Lee WR. Toxicity and quality of life report of a phase II study of stereotactic body radiotherapy (SBRT) for low and intermediate risk prostate cancer. *Radiat Oncol* 2017; **12**: 14.

12 McBride SM, Wong DS, Dombrowski JJ, *et al.* Hypofractionated stereotactic body radiotherapy in low-risk prostate adenocarcinoma: Preliminary results of a multi-institutional phase 1 feasibility trial. *Cancer* 2012; **118**: 3681–90.

13 Bolzicco G, Favretto MS, Scremin E, Tambone C, Tasca A, Guglielmi R. Image-guided stereotactic body radiation therapy for clinically localized prostate cancer: Preliminary clinical results. *Technol Cancer Res Treat* 2010; **9**: 473–7.

14 Alongi F, Fersino S, Mazzola R, *et al.* Phase 2 Study With FFF LINAC-Based SBRT in Five Consecutive Fractions for Localized Prostate Cancer. In: International Journal of Radiation Oncology*Biology*Physics. 2017: E210–1.

15 Alongi F, Cozzi L, Arcangeli S, *et al.* Linac based SBRT for prostate cancer in 5 fractions with VMAT and flattening filter free beams: Preliminary report of a phase II study. *Radiat Oncol* 2013; **8**: 171.

16 Madsen BL, Hsi RA, Pham HT, Fowler JF, Esagui L, Corman J. Stereotactic hypofractionated accurate radiotherapy of the prostate (SHARP), 33.5 Gy in five fractions for localized disease: First clinical trial results. *Int J Radiat Oncol Biol Phys* 2007; **67**: 1099–105.

The PACE Trial

(Prostate Advances in Comparative Evidence)
International randomised study of prostatectomy vs stereotactic body radiotherapy (SBRT) and conventional radiotherapy vs SBRT for early stage organ-confined prostate cancer

PROTOCOL

Version: 9
Dated: 14th June 2017

Chief Investigator:	Dr Nicholas van As
Sponsor:	Royal Marsden NHS Foundation Trust<br>Fulham Road, London, SW3 6JJ
Funders:	Accuray
Coordinating Trials Unit:	ICR Clinical Trials and Statistics Unit (ICR-CTSU)<br>The Institute of Cancer Research

Main REC Reference Number: LO/11/1915
ICR-CTSU Protocol Number: ICR-CTSU/2015/10053
CCR Number: CCR3766
ISRCTN: 17627211
ClinicalTrials.gov Identifier: NCT01584258
CRUK Reference Number: CRUKE/12/025

PACE is part of the National Institute for Health Research Clinical Research Network Trial Portfolio
PACE-B is endorsed by Cancer Research UK

This protocol is a controlled document and should not be copied, distributed or reproduced without the written permission of the ICR-CTSU

CONFIDENTIAL

1 Trial management contact information

ICR-CTSU (a UKCRC registered CTU and an NCRI cancer clinical trials unit) is responsible for the central coordination of the trial on behalf of the Sponsor.

ICR-CTSU Scientific Lead : Emma Hall
Tel: +44 (0)20 8722 4013
Emma.Hall@icr.ac.uk

ICR-CTSU Statistician: Clare Griffin/ Keeley Tomkinson
Tel: +44 (0)20 8722 4101
Clare.Griffin@icr.ac.uk
Keeley.Tomkinson@icr.ac.uk

ICR-CTSU Senior Trial Manager: Stephanie Burnett
Tel: +44 (0)20 722 4261
Stephanie.Burnett@icr.ac.uk

PACE Trial Manager: Stephanie Brown
Tel: +44 (0)20 8722 4467
pace-icrctsu@icr.ac.uk

Clinical Research Fellow: Dr Kirsty Morrison
Tel: +44 (0)20 7811 8469
Kirsty.Morrison@rmh.nhs.uk

Radiotherapy Trials QA Physicist: Olivia Naismith
Tel: +44(0)20 7808 2500
Oliva.naismith@rmh.nhs.uk

PACE trial Sponsor: Royal Marsden NHS Foundation Trust
Address: Fulham Road, London, SW3 6JJ
Telephone: +44 (0) 208 642 6011

Any questions relating to this protocol should be addressed in the first instance to the PACE Trial Manager within ICR-CTSU:

PACE Trial Manager
ICR Clinical Trials & Statistics Unit (ICR-CTSU),
Division of Clinical Studies,
The Institute of Cancer Research,
Sir Richard Doll Building,
Cotswold Road,
Sutton, Surrey SM2 5NG

Email: pace-icrctsu@icr.ac.uk
General enquiries: +44(0)20 8722 4467
Fax: +44(0)20 8770 7876

PROTOCOL DEVELOPMENT GROUP

Dr Shafak Aluwini
Professor Volker Budach
Dr Luca Incrocci
Professor Eric Lartigau
Dr Peter Ostler
Mr Alan Thompson
Dr Alison Tree
Dr Daniel Henderson
Dr Irving Kaplan
Dr Martin Sanda
Dr Mischa Hoogeman

Version: 9, 14th June 2017

CONFIDENTIAL

2 Protocol signature page

The PACE Trial: International randomised study of prostatectomy vs stereotactic body radiotherapy (SBRT) and conventional radiotherapy vs SBRT for early stage organ-confined prostate cancer

The Trial Management Group (TMG) will be constituted from members of the Protocol Development Group and will include the Chief Investigator, ICR-CTSU Scientific Lead, Co-investigators and identified collaborators, the Trial Statistician and Trial Manager. Principal Investigators and key study personnel will be invited to join the TMG as appropriate to ensure representation from a range of sites and professional groups. Where possible, membership will include a lay/consumer representative. A copy of the current membership of the TMG can be obtained from the PACE Trial Manager at ICR-CTSU.

Protocol Authorised by:

Name & Role	Signature	Date
Dr Nicholas van As<br>(Chief Investigator)		

This protocol describes the PACE trial and provides information about procedures for entering participants into this trial. The protocol should not be used as a guide for the treatment of patients outside of this trial.

Every care was taken in the preparation of this protocol, but corrections or amendments may be necessary. Protocol amendments will be circulated to participating sites as they occur, but sites entering patients for the first time are advised to contact ICR-CTSU to confirm they have the most recent version.

Table of contents

1 TRIAL MANAGEMENT CONTACT INFORMATION 2

2 PROTOCOL SIGNATURE PAGE 3

3 STUDY SUMMARY 5

4 STUDY SCHEMA 8

5 BACKGROUND 9

6 RATIONALE 9

7 STUDY OBJECTIVES 19

8 STUDY DESIGN 19

9 PATIENT SELECTION 22

10 STUDY ASSESSMENTS AND RANDOMISATION PROCEDURES 24

11 TREATMENT 33

12 ADVERSE EVENT REPORTING 41

13 STATISTICAL CONSIDERATIONS 45

14 QUALITY ASSURANCE (QA) 53

15 ETHICAL AND REGULATORY ASPECTS 54

16 STUDY MANAGEMENT AND OVERSIGHT 57

17 STUDY SPONSORSHIP 58

18 PUBLICATION POLICY 58

19 APPENDICES 59

20 REFERENCES 63

3 Study Summary

**Title**	The PACE trial: International Randomised Study of Prostatectomy vs Stereotactic Body Radiotherapy (SBRT) and Conventional Radiotherapy vs SBRT for Early Stage Organ-Confined Prostate Cancer
**Aim**	In the primary management of early stage organ-confined prostate cancer, to assess whether hypofractionated stereotactic body radiotherapy (SBRT) offers benefit over prostatectomy or conventional radiotherapy.
**Design**	Multicentre, international phase 3 randomised controlled study comprising two parallel randomisations with a common experimental arm.
**Objectives**	<u>In PACE-A:</u><br>Primary: To determine whether there is improved quality of life following prostate SBRT compared with prostatectomy two years from completion of trial treatment.<br><br><u>In PACE-B:</u><br>Primary: To determine whether prostate SBRT is non inferior to conventional radiotherapy for freedom from biochemical/clinical failure in low/ intermediate risk prostate cancer.<br><br><u>In PACE-A and PACE-B, common secondary objectives:</u><br>To determine the relative benefits of surgery, radiotherapy and prostate SBRT in terms of local failure, distant failure, disease-free survival, disease-specific survival, overall survival, toxicity, quality of life in generic and organ specific domains.
**Primary end-points**	<u>In PACE-A:</u><br>Co-primary patient reported outcomes::<br>(1) Urinary incontinence (number of absorbent pads required per day to control leakage) measured by The Expanded Prostate Cancer Index (EPIC) questionnaire.<br>(2) Bowel bother summary score from the EPIC questionnaire.<br><br>The main time point of interest is 2 years post treatment.<br><br><u>In PACE-B:</u><br>Freedom from biochemical (Phoenix definition for conventional radiotherapy and SBRT arms, >0.2 ng/ml for surgical arm) or clinical (commencement of androgen deprivation therapy) failure. The main time point of interest is 5 years from randomisation.
**Secondary end-points**	<u>In PACE-A:</u><br>• Freedom from biochemical (Phoenix definition for conventional radiotherapy and SBRT arms, >0.2 ng/ml for surgical arm) or clinical (commencement of androgen deprivation therapy) failure. The main time point of interest is 5 years post treatment.<br><br><u>In PACE-A and B:</u><br>• Clinician reported acute toxicity using CTCAE, RTOG (SBRT and conventional radiotherapy patients only) and Clavien (surgical patients only) scales.<br>• Clinician reported late toxicity using CTCAE and RTOG (SBRT and conventional radiotherapy patients only) scales.<br>• Patient reported acute and late bowel, bladder and erectile dysfunction symptoms. Assessed using IIEF-5, IPSS, Vaizey score and EPIC-26 instruments.<br>• Disease-specific and overall survival<br>• Progression-free survival– radiographic, clinical or biochemical evidence of local

	or distant failure.<br>• Commencement of androgen deprivation therapy (LHRH analogues, anti-androgens, orchidectomy).
**Hypothesis**	• Profound hypofractionation with SBRT has the potential to achieve equivalent tumour control rates compared to surgery and conventional radiotherapy while reducing radiation to normal tissues (bladder, rectal and penile bulb) and minimising radiation-induced side effects.<br>• Profound hypofractionation with SBRT has the potential to improve quality of life compared with prostatectomy.
**Treatment**	<u>In PACE A</u>: Patients considered candidates for surgery, agreed by both the physician and patient, are randomised to either prostatectomy or prostate SBRT delivered with 36.25 Gy in 5 fractions.<br><br><u>In PACE B</u>: Nonsurgical candidates or patients who decline surgery will be randomised to either prostate SBRT (36.25 Gy in 5 fractions) or conventional radiotherapy (investigators choice between 78 Gy in 39 fractions or 62 Gy in 20 fractions).
**Eligibility criteria**	*Inclusion criteria:*<br>• Histological confirmation of prostate adenocarcinoma with a minimum of 10 biopsy cores taken within the last 18 months (unless on active surveillance and not clinically indicated – see section 9, Patient selection).<br>• Gleason score ≤ 3+4<br>• Men aged ≥18 years at randomisation<br>• Clinical and/or MRI stage T1c –T2c, N0-X, M0-X<br>• PSA ≤ 20 ng/ml<br>• Pre-enrollment PSA must be completed within 60 days of randomisation<br>• Patients belonging in one of the following risk groups according to the National Comprehensive Cancer Network (www.nccn.org): See Appendix 2<br>○ Low risk: Clinical stage T1-T2a and Gleason ≤ 6 and PSA < 10 ng/ml, or<br>○ Intermediate risk includes any one of the following:<br>• Clinical stage T2b orT2c<br>• PSA 10-20 ng/ml or<br>• Gleason 7 (3+4 for PACE)<br>• WHO performance status 0 - 2<br>• Ability of the research subject to understand and the willingness to sign a written informed consent document<br><br>*Exclusion Criteria:*<br>• Clinical stage T3 or greater<br>• Gleason score ≥ 4 + 3<br>• High risk disease defined by National Comprehensive Cancer Network (www.nccn.org): See Appendix 2<br>• Previous malignancy within the last 2 years (except basal cell carcinoma or squamous cell carcinoma of the skin), or if previous malignancy is expected to significantly compromise 5 year survival.<br>• Prior pelvic radiotherapy<br>• Prior androgen deprivation therapy (including androgen agonists and antagonists)<br>• Any prior active treatment for prostate cancer. Patients previously on active surveillance are eligible if they continue to meet all other eligibility criteria.<br>• Life expectancy <5 years

	• Bilateral hip prostheses or any other implants/hardware that would introduce substantial CT artifacts<br>• Medical conditions likely to make radiotherapy inadvisable eg inflammatory bowel disease, significant urinary symptoms<br>• For patients having fiducials inserted: Anticoagulation with warfarin/ bleeding tendency making fiducial placement or surgery unsafe in the opinion of the clinician (see section 11, Treatment).<br>• Participation in another concurrent treatment protocol for prostate cancer
**Target sample size**	PACE A: 234 (117 patients per arm)<br>PACE B: 858 (429 patients per arm)

4 Study schema

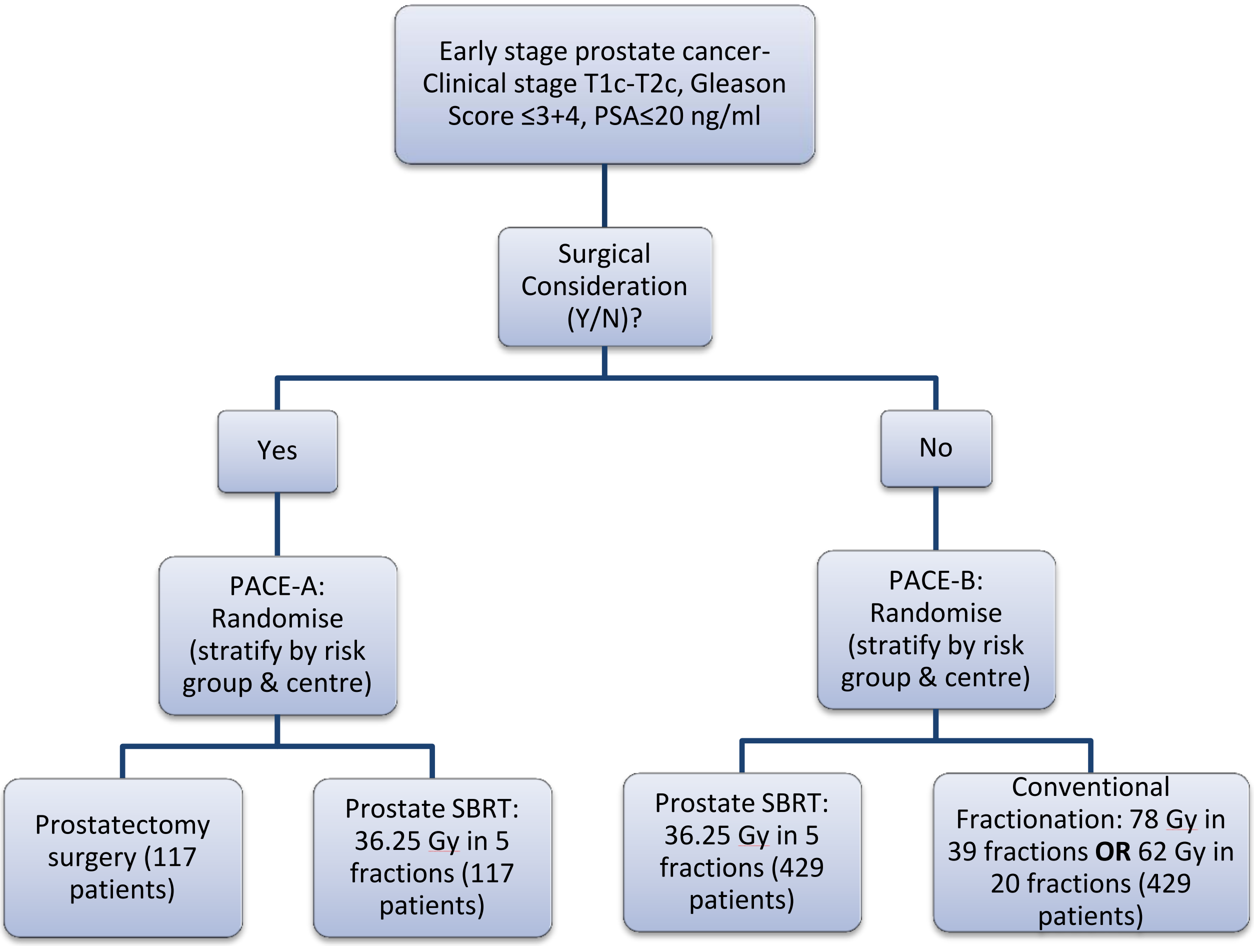

Patients seen 4 times within the first 3 months of follow-up with clinician reported (RTOG bladder and bowel toxicity and CTCAE) and patient reported (IPSS and/or EPIC-26, IIEF-5 and Vaizey) acute toxicity assessed.

Thereafter patients are seen 3-12 monthly with clinician reported (RTOG bladder and bowel toxicity and CTCAE until 10 years post-treatment) and patient reported (IPSS, EPIC-26, IIEF-5 and Vaizey until 5 years post-treatment) late toxicity assessed.

Statistical design:
PACE-A: 234 patients provides 80% power to detect an 11% difference in urinary incontinence at 2 years assuming 15% in the control arm. This number of patients also provides over 90% power to detect a 5 point difference in mean bowel bother scores.

PACE-B: 858 patients provides 80% power to rule out a detriment of at most 6% (non-inferiority margin) in biochemical or clinical failure at 5 years assuming the proportion of patients biochemical progression-free is 85% in the control arm (critical hazard ratio = 1.45).

5 Background

There are several treatment options for men with early stage prostate cancer. At present these are held in a therapeutic equipoise as neither surgery nor radiotherapy has been proven to be superior. Historically, most trials that have attempted to randomise between surgical and radiation treatments have been unsuccessful and have failed due to the inherent difficulties in convincing patients to accept such different treatment modalities by chance. One study has successfully randomised between surgery and radiotherapy in prostate cancer. The ProtecT study has successfully recruited over 500 men to each of the study arms, which are external beam radiotherapy, surgery and active surveillance. This impressive feat has been made possible by conducting detailed studies of recruitment interviews and the way patients decide on their treatment. It is thought that the key to this trial's success is funding dedicated well trained trial nurses who conduct the recruitment interviews. This robust academic approach should form the backdrop to this trial.

As physicians caring for men with prostate cancer we wish to be able to offer patients the best advice. At present we cannot tell them whether surgery, or radiotherapy or SBRT would be the more efficacious or safer treatment choice or whether there is improved quality of life with SBRT. This study is designed to help to answer these questions.

In addition, there are many radiobiological, technological, economic and practical reasons why a 5 fraction hypofractionated SBRT treatment regimen may be advantageous for patients, but before clinical practice changes we must establish conclusively if profound hypofractionation is at least as good as conventional regimens. This study is also designed to answer that question

6 Rationale

6.1 Epidemiology and background

Prostate cancer is the most common non-cutaneous cancer in men, and since the introduction of serum prostate specific antigen (PSA) testing, the majority of cases are diagnosed with early stage, organ confined disease, which is often asymptomatic. In Europe the incidence of prostate cancer was 370,733 new cases in 2008 [1], and the rates across Europe and in the USA are amongst the highest in the world [2]. Almost 60% of new cases of prostate cancer will be in men over the age of 70 years. Despite the volume of cases, the assessment and management of organ-confined prostate cancer remains challenging and controversial. Radical prostatectomy has been shown in a good quality randomised controlled trial to have an overall survival advantage compared with watchful waiting [3]. There are however several treatment options for early prostate cancer in addition to surgery. Fractionated external-beam radiotherapy, brachytherapy (HDR or LDR) and, for selected patients, active surveillance are all considered to be effective methods for treating prostate cancer. No superiority has yet been shown In terms of survival, and so all suitable options are discussed with men to enable treatment tailored to their circumstances and preferences.

The Prostate Testing for Cancer and Treatment (ProtecT) trial randomised over 1600 men aged 50 to 69 years with localized prostate cancer to active monitoring, prostatectomy or external beam radiotherapy (74Gy in 37 fractions) with neoadjuvant hormones. Results demonstrate a low prostate cancer specific mortality of less than 2% at 10 years median follow up, with no significant difference between the three treatment arms. The active monitoring group had a higher rate of disease progression and development of metastases in comparison to the radical treatment arms, however there was no difference between surgery and radiotherapy[4].

Radiotherapy is an extremely effective treatment for prostate cancer, but conventional treatments are protracted over 7-9 weeks, which impact the patient's quality of life and utilization of hospital resources. There is a compelling argument for treating prostate cancer using hypofractionation. In general, increased radiation fractionation provides an increasing therapeutic advantage between tumour control and late treatment related side effects. However, studies deriving the alpha-beta ratio for prostate cancer from low dose rate brachytherapy treatments have suggested the alpha-beta ratio is possibly as low as 1.5 Gy (see Section 6.6 below). If these estimates are accurate, they would predict that hypofractionated schedules for prostate cancer should produce tumour control and late treatment related sequelae that are at least as good or better than those currently achieved with current conventional schedules using 1.8-2.0Gy daily fractions.

More recently, three large studies have reported outcomes in patients treated with moderate hypofractionation. Most importantly, the CHHiP trial randomised more than 3200 patients between 74 Gy in 37 fractions (the control arm), 60 Gy in 20 fractions, and 57 Gy in 19 fractions [5]. A short course of androgen deprivation was given to all patients. The majority of patients (88%) were NCCN intermediate or low risk. At a median follow-up of 62 months, estimated 5 year PSA progression-free survival was 88.3%, 90.6%, and 85.9% for the 74Gy, 60Gy and 57Gy groups respectively. Although the investigators found an increase in grade 2+ RTOG acute bowel toxicity in the hypofractionated groups (24.6% for 74 Gy, 38.5% for 60 Gy, and 37.9% for 57 Gy; $p < 0.001$), the differences had disappeared 18 weeks after the start of radiotherapy and late toxicity was low and less than 4% in all groups at 2 years. By five years, there was no significant difference in RTOG bowel toxcity between the three groups (1.3%, 2.3%, and 2.0%, respectively). No significant differences between the groups were found with respect to acute or late urinary toxicity. The investigators concluded that 60 Gy in 20 fractions was non-inferior to 74 Gy (HR 0.84, 90% CI 0.68, 1.03 with HR<1.0 being in favour of 60Gy group) and could be recommended as a new standard of care. However, 57 Gy in 19 fractions was not shown to be non-inferior (HR 1.20, 90% CI 0.99, 1.45). It is expected that the 60 Gy in 20 fraction dose (given with hormones) will be widely adopted in the UK, particularly in view of its favourable impact on radiotherapy resource use.

In the HYPRO study [6]. 820 patients were randomised between 78 Gy in 39 fractions (the control arm) and 64.6 Gy in 19 fractions over 6.5 weeks (treating three times per week). This group found that the incidence of acute G2+ RTOG rectal toxicity was significantly higher in the hypofractionated cohort (31.2% vs 42.0%; $p = 0.0015$). However, this difference was not maintained to 3 months and the authors themselves point out that their trial was underpowered for this comparison. It is also important to note that 64.6 Gy in 19 fractions dose is a significantly higher biologically equivalent dose than that used in the CHHiP trial. No significant differences were found in acute urinary toxicity between the groups. At 5 years median follow-up, there were no significant differences in biochemical relapse free survival [7]. However, the late follow-up is only published in abstract form, and further toxicity data are awaited.

Finally RTOG Trial 0415 [8] established that hypofractionated delivery of 70.0 Gy in 28 fractions over 5.6 weeks is noninferior to conventional delivery of 73.8 Gy in 41 fractions over 8.2 weeks. 1115 patients with low-risk disease were randomly assigned to the conventional RT schedule or the hypofractionated schedule. No androgen suppression was permitted. After a median follow-up of 5.9 years, the 7-year disease-free survival (DFS) rate for hypofractionated RT was not lower than that for conventional RT by more than 7% (hazard ratio [HR] < 1.52) with the estimated 7-year DFS rate of 82% for hypofractionated RT compared with the rate of 76% for conventional RT (HR 0.85, 95% CI [0.64, 1.14]). There was also noninferiority for the biochemical recurrence endpoint (HR 0.77, 95% CI [0.51, 1.17]). Hypofractionated RT delivery produced an increase in late gastrointestinal and genitourinary toxicity however there was no statistically signifanct difference in the risk of Grade 3 or more GI events (relative risk (RR) 1.53, 95% CI [0.86, 2.83]) or GU events (RR 1.43, 95% CI [0.86, 2.37]).

The next logical question to answer is whether "profound" hypofractionation could produce non-inferior results. Historically, delivery of larger fraction sizes is limited by normal tissue constraints and the requirement for large planning margins. SBRT however offers the opportunity to accurately deliver larger fractions with a high degree of accuracy. Early data from the two Accuray sponsored studies of either 5 fractions with a homogenous dose distribution [9] or 4 fractions with an HDR like heterogenous dose distribution [10] show that early toxicity is low. A large series of over 1000 patients now confirm that SBRT results in 5-year biochemical control rates similar to those seen with conventional fractionation, and is associated with only transient declines in quality of life [11, 12]. Whilst this data is encouraging, without a phase III trial we cannot conclude that SBRT is equivalent to conventional therapies.

6.2 Surgical management of organ-confined prostate cancer

Radical prostatectomy and radiotherapy are considered to be treatments of choice for early prostate cancer.

A large Spanish center has published its experience of treating 505 men with early prostate cancer (approximately half were low risk and half were intermediate risk). The 5-year biochemical relapse free survival (bRFS) for the radical prostatectomy and external beam radiation therapy (EBRT) cohorts were 79% and 86% respectively [13]. However, many of the subjects in the EBRT cohort received what would today be considered as sub-optimal doses of radiotherapy. A total of 25% of subjects who underwent surgery reported urinary incontinence (measured using IPSS and EPIC questionnaires).

Kupelian et al. published a retrospective cohort of 1877 patients who received either prostatectomy or radical radiotherapy (median dose 70.2Gy)[14]. Both treatments resulted in a similar bRFS (70-72%), despite the radiation dose used now being considered suboptimal. In a further analysis with nearly twice as many patients, they documented a higher 5-year bRFS of 81%, which was the same for radical prostatectomy and EBRT if >72 Gy was given [15]. Biochemical relapse was defined as 0.2ng/ml for the surgical arm and three successive PSA rises (ASTRO definition) for the radiotherapy arm.

A similar retrospective study, looking at the Memorial Sloan Kettering experience revealed a 7-year bRFS rate of 79% for radical prostatectomy and 77% for EBRT [16].

For the cohort of patients eligible for this study prostate cancer specific mortality (PCSM) is likely to be low. In a large retrospective series, PCSM for patients with Gleason 7 or less was 2-5% at 15 years [17]. Another earlier study documented an 82% metastasis free survival at 15 years in patients treated at a single center [18]. The median time to metastasis from PSA elevation was 8 years (these men received no salvage therapy prior to documented metastatic disease). Once metastatic disease had been diagnosed, the median time to death was 5 years. The ProtecT trial has demonstrated no significant differnence in prostate-cancer specific survival after 10 years follow up between the three randomised groups: surgery (RP), radiotherapy (RT) and active monitoring (AM) (RT vs. AM: hazard ratio (HR): 0.51 (95%CI: 0.15 to 1.69), RT vs. RP: HR: 0.80 (0.22 to 2.99), RP vs AM: HR: 0.63 (0.21 to 1.93). Within the prostatectomy group the 10 year prostate cancer specific survival was 99% (95% CI: 97.2 to 99.6)[4].

6.3 Toxicity of prostatectomy and radiotherapy

The relative toxicity of the treatment options is currently an important parameter for men deciding upon treatment. The surgical literature often reports a 'Trifecta' outcome of biochemical control with continence and return of erectile function. This is the gold-standard outcome for surgery [19].

6.3.1 Urinary toxicity

After the so-called learning curve for laparoscopic procedures, the 12-month urinary continence post prostatectomy rates vary from 75-95%, depending on age and definition of continence (leak free vs pad free) [20].

Patient reported outcomes in the ProtecT trial included urinary incontinence measured using the EPIC questionnaire at baseline, 6 and 12 months and then annually to six years. At 2 years, 20% (80/399) of patients who had radical prostatectomy reported any use of absorbent pads compared with 4% (16/394) in those who had radical radiotherapy[21]. Scores for voiding symptoms were seen to be worse in the radiotherapy group at 6 months follow up but then returned to baseline levels similar to other treatment groups. The CHHiP hypofractionation trial also collected information on urinary pad use using the EPIC questionnaire and reported 2.8% (36/1272) of patients receiving radiotherapy (across all radiotherapy regimens) using at least one absorbent pad at 2 years.

6.3.2 Erectile function

Erectile function post-prostatectomy has also been reviewed by Ficarra and colleagues [20]. They identified two studies which used a validated questionnaire International Index of Erectile Function (IIEF) and these found rates of potency sufficient for intercourse of between 33 and 46% at 3 months post surgery. There was no significant difference between techniques.

It is important to note that radiotherapy, as well as surgery, can produce erectile dysfunction. Dose-volume parameters have not been well established for the prevention of erectile dysfunction due to radiotherapy. Traditionally the penile bulb is contoured and a dose-constraint applied to this volume, but the penile bulb itself plays a minor role in erectile function, and correlations between dose and function have not been consistently shown [22]. Data from a small cohort of the RT01 patients did show a correlation between D90 >50 Gy to the penile bulb [23].

Roach and colleagues in a recent review of the subject, agree that the data is conflicting but present their own and others data supporting a correlation between dose and function [24]. They advise that the mean dose to 95% of the penile bulb should be treated to <50 Gy with conventional fractionation.

A meta-analysis of rates of erectile dysfunction after treatment [25] compared various modalities of treatment. The chance of maintaining erectile function at 2 years post-treatment, assessed using patient questionnaires, was 25% (18 -33% confidence intervals) for nerve-sparing prostatectomy and 52% for EBRT (95% confidence intervals 48-56%). The average age of men undergoing EBRT was 69.5 years and 61 years for nerve-sparing prostatectomy.

Although some of the data included in the above meta-analysis is older, newer series of radical prostatectomies indicate similar levels of erectile dysfunction. A single institution study from Germany reports that at 1-year post surgery, only 26% of men had returned to their baseline potency rates, although the rate of nerve-sparing surgery was only 54% [26]. For the subgroup who had nerve-sparing surgery and were potent at baseline, the rate of potency at 12 months was 56%.

The relative risk of erectile dysfunction with radiotherapy compared to radical prostatectomy is still hotly contested [27] but a large prospective study of 1201 patients treated with surgery, EBRT or brachytherapy has shown that sexual function parameters for quality of life were worse for surgical patients (and their partners) compared with radiotherapy patients [28].

Within the ProtecT trial, baseline erectile function were similar across treatment groups with 67.5% of men reporting an erection firm enough in the AM group, 65.7% in the RP group and 68.4 in the RT group. At 2 years follow up, the AM group had 47.1% of men with erections firm enough for intercourse compared to 34.0% in the RT group and 18.9% in the RP group (p<0.001). This pattern of reduced erectile function post prostatectomy continued into longer term follow up. [21]

The IIEF-5 is a validated diagnostic tool for diagnosing erectile dysfunction in men [29] and will be used to monitor men in this study.

6.3.3 Bowel bother

Bowel function and bother scores were assessed in the ProtecT trial using the EPIC questionaire[30]. At 6 months, the bowel summary score for the AM and RP groups were unchanged from baseline (~9%), however the RT group had scores increased from 7% at baseline to 16% at 6 months (p<0.001). At 2 years, 7.4% of the men in the RT group reported bloody stools about half the time or more frequently compared with 0.3% in the RP group and 0.8% in the AM group (p<0.001). A similar pattern continued into future follow up[21].

6.4 What should be our conventional radiotherapy arm?

Dose escalation studies have proven that higher doses are associated with improved cure rates:

Dearnaley et al. conducted a pilot for a phase III trial randomising to 64 Gy vs 74 Gy and reported 5 year biochemical control rates of 59% (standard dose) and 71% (escalated dose) (HR 0.64, 95% CI 0.38–1.10, P=0.10) with acceptable acute and late toxicity [31]. The subsequent MRC RT01 trial randomised 862 men to the same fractionation regimens and found that at 6 months post-radiotherapy grade 2 or higher toxicity was low [32]. However almost all of this toxicity was seen in the group receiving 74 Gy. In both arms the radiotherapy was given in conjunction with androgen deprivation. This trial did also confirm an increase in biochemical progression-free survival (60% with the lower dose and 71% with the higher dose at 5 years follow-up, hazard ratio of 0.67 for clinical progression in the higher dose arm, CI 0.53-0.85, p=0.0007) and metastasis-free survival, in addition to a reduction in need for salvage androgen suppression [33]. After 10 years follow-up in the MRC RT01 trial, the higher dose continued to show a benefit over the lower dose in terms of biochemical progression free survival with estimates of 55% and 43% respectively [HR 0.69, 95%CI 0.56-0.84, p=0.0003). However, this benefit did not translate into an improvement in overall survival with 71% overall survival in both groups at 10 years [34].

Kupelian et al. pooled the data from nine institutions totalling over 4800 men. Despite the higher dose cohort (>72 Gy) having worse prognostic features, their 5-year biochemical disease-free survival (bDFS) was significantly improved compared to the cohort who received <72 Gy [35].

The MD Anderson group conducted a phase 3 trial comparing 70 Gy to 78Gy without androgen deprivation and found a significant improvement in freedom from failure (including biochemical failure) in the higher dose group (freedom from failure at 6-years 64% vs 70%, p=0.03) [36]. This included a reduction in the incidence of distant metastasis in the subgroup of patients with a PSA >10 ng/ml at 6 years of follow-up. However this trial also confirmed an increase in rectal side effects in the higher dose arm (grade 2 or higher toxicity 26% vs 12%). This trial was conducted in the era before image-guided radiotherapy (IGRT) and intensity-modulated radiotherapy (IMRT) were standard and hence higher doses are likely to be deliverable with less toxicity today.

Peeters et al also conducted a dose escalation trial randomising 664 men 68Gy or 78Gy. The higher dose was associated with a 10% increase in freedom from failure at 5 years (HR 0.74, p= 0.02) [37].

Most studies have shown that a higher dose is associated with more toxicity, but in general 78 Gy has tolerable toxicity. The EORTC 22991 trial showed 1% grade 3 gastrointestinal (GI) toxicity and 6.2% grade 3 GU toxicity, without a significantly increased rate at the higher dose levels (up to 78 Gy) [38]. The 2012 EAU guidelines on prostate cancer suggest 78 Gy is a good compromise of efficacy and tolerability [39].

These data suggest that 78 Gy in 39 fractions would be a suitable radiotherapy dose for use in the control arm for this study.

Following publication of the CHHIP trial results (described in Section 6.1), it is likely that many centres will adopt a 20 fraction schedule as a new standard of care (although a number of centres would be expected to continue using 2 Gy per fraction schedules).

Given that CHHIP has shown that a dose of 60 Gy in 20 fractions is non-inferior to the control arm dose of 74 Gy in 37 fractions it is valid to include an option for investigators to use a moderately hypofractionated treatment in the PACE control arm, at their discretion. The control arm in PACE is 78 Gy in 39 fractions, 5.4% higher than that in the CHHIP control arm (discounting the two additional days of treatment time). Data from the CHHIP trial also implies that the α/β ratio for prostate cancer lies between 1.5 and 2.5 Gy. Keeping to a 20 fraction dose, and using an α/β ratio of 2, a dose 5.4% higher than 60 Gy in 20 fractions (BED = 150 Gy) is 62 Gy in 20 fractions (BED = 158.1). This calculation is relatively insensitive to α/β ratio, being 61.8 Gy for α/β = 1, and 62.1 Gy for α/β = 3.

Therefore, a dose of 62 Gy in 20 fractions over 4 weeks (3.1 Gy per fraction) is a suitable alternative to the conventionally fractionated dose in the control arm of PACE.

6.5 Dose-volume constraints for conventional radiotherapy

Late rectal toxicity increases with the dose and volume of rectum irradiated. There is a wealth of literature on the correlation between dose and rectal complications and this was thoroughly reviewed by Fiorino and colleages in 2009 [40]. It seems that keeping the V70 Gy <25% and the V75 Gy below 5% results in a low incidence of rectal bleeding using conventional fractionation [40].

Other factors can play a role in the risk of late rectal bleeding including diabetes, previous abdomino-pelvic surgery and possibly androgen deprivation therapy [40]. Whilst rectal bleeding seems to be most closely associated with the higher doses received by the rectum, the risk of faecal incontinence, whilst low, seems related to the lower doses [40].

In order to develop constraints for the 62 Gy in 20 fractions control arm option, those used in the 78 Gy in 39 fraction group were scaled using the methods in the CHHiP trial.

6.6 Why hypofractionate at all? The radiobiological argument

As discussed above, it is clear that increasing the dose to the prostate increases cure rates at the expense of increased side effects. However, it may be possible to simultaneously increase cure rates whilst decreasing toxicity by exploiting the unusual radiobiology of prostate cancer. For most cancers the alpha/beta ratio is high (around 10 Gy) indicating that these tissues are more sensitive to total radiation dose, rather than dose per fraction. For the late-reacting surrounding normal tissues the alpha/beta ratio is low (around 3) indicating a higher sensitivity to fraction size.

There is now growing evidence that the alpha/beta ratio for prostate cancer cells is lower than that of surrounding normal tissue, and may be as low as 1.5 Gy. This means that by increasing fraction size and reducing total dose would be expected to increase cure rates and decrease

toxicity. Recent, very large (n>5000) patient datasets have been used to derive the α/β ratio of prostate cancer [41, 42] and estimates consistently fall around the 1.4 Gy mark.

As mentioned above, the α/β ratio of the late-reacting normal tissues is usually assumed to be 3 Gy. For prostate cancer patients, the dose-limiting structure is the rectum which lies in close proximity to the prostate gland.

Marzi et al. randomised patients to 80 Gy in 40- fractions or 62 Gy in 20 fractions and showed similar toxicity [43]. From their data they estimated the α/β ratio of the rectum for late toxicity to be around 3 Gy.

The rectal toxicity data from the RTOG 94-06 trial was analysed and the best fit α/β ratio for late rectal damage was 4.6 Gy although the confidence intervals were wide [44].

Further data has emerged from the CHHIP trial results (see Section 6.1). Although 60 Gy in 20 fractions was non-inferior to 74 Gy in 37 with respect to efficacy, 57 Gy in 19 fractions was not non-inferior. The 60 Gy in 20 fractions dose was calculated to be equivalent to 74 Gy in 37 fractions with an α/β ratio of 2.4 Gy, while the 57 Gy in 19 fractions dose was equivalent with α/β ratio 1.4 Gy. The results of this study suggest (in the absence of a time-factor) that the true α/β ratio lies between 1.4 and 2.4 Gy.

Taken together, the α/β ratio of prostate cancer appears to be significantly lower than that of the rectum, which means that the higher the dose per fraction, the higher the cell kill to the prostate cancer. This should be accompanied by a reduction in the incidence of rectal side effects due to the lower total dose required.

6.7 Existing studies of moderate hypofractionation – what do we already know?

There are now many trials which have investigated the role of hypofractionation in prostate cancer. However there is also historical data supporting the efficacy and tolerability of a hypofractionated regimen. Between 1962 and 1984 Lloyd-Davies et al. treated 209 patients with apparently localised prostate cancer. Over 90% of this cohort received 36 Gy in 6 fractions over 18 days. They report a 5 year survival of 68% [45] and the toxicity of this regimen appears very good [46] especially as the standard fields in that era were non-conformal.

Livesey et al. reported data on 705 men with a wide range of prostate cancer (T1-T4) treated with 50 Gy in 16 fractions in the late 1990s. The bRFS for the low-risk patients was 82% at 5 years, with acceptable GU and gastrointestinal (GI) toxicity of 5% and 9% respectively [[47].

Soete et al. used 56 Gy in 16 fractions over 4 weeks (3.6 Gy/fraction) and noted an increase in acute side effects of grade 1-2 compared to previous cohorts of patients but no acute grade 3 toxicity was recorded [48]. The international prostate symptom index (IPSI) had returned to baseline scores by two months post treatment.

Martin et al. treated 92 patients with 60 Gy in 20 fractions over 4 weeks with acceptable toxicity and a 97% bRFS at 14 months. At a median follow-up of 38 months, no patients had grade 3 toxicity recorded at their last follow-up [49]. This good toxicity data is echoed by Yeoh et al. who treated 217 men to a dose of 55 Gy in 20 fractions or 64 Gy in 32 fractions [50, 51] and found that the toxicity rates were approximately equal but with a superior bRFS at 90 months [50].

Most recently, as discussed in Section 6.1, the CHHIP trial, randomising over 3200 patients has confirmed that 60 Gy in 20 fractions given over 4 weeks is non-inferior to 74 Gy in 37 fraction at 5 years with regard to biochemical/clinical failure. Late G2+ bowel and bladder RTOG toxicity was low, with no significant differences between the groups at 5 years.

6.8 Experience with profound hypofractionation using brachytherapy

Many men, over many years, have been treated with hypofractionated radiotherapy in the form of HDR brachytherapy. Using this technique, fractionation regimens of 48 Gy in 8 fractions or 54 Gy in 9 fractions over 5 days have demonstrated 70% PSA failure-free survival at 5 years, despite the majority of these patients having high risk disease [52]. Relapse-free survival at 3 years was 100% for the low risk patients included in this study. Five percent of patients had grade 3 acute GU toxicity and 21% had grade 2 acute GU toxicity. With regard to late toxicity, one patient had a grade 3 GI toxicity, and 11% had grade 2 GU toxicity. Yoshioka et al. updated their results in 2010 and had treated 112 men with 54 Gy in 9 fractions with HDR brachytherapy [53]. The majority of these patients had high risk disease and also received androgen deprivation therapy (ADT). Overall 5-year bRFS was 83%. This was achieved with 5% acute and 3% late grade 3 toxicity.

Another cohort of 117 consecutive patients were treated with escalating doses of 6 fraction HDR from 36 Gy to 43.5 Gy, delivered in 2 insertions one week apart [54]. They report excellent 8 year bRFS of 94% for this group of low and intermediate-risk prostate cancer patients. Four (3%) patients had grade 3 late urinary toxicity.

Recently Demanes et al. have described their experience of treating 298 men with mostly low and low-intermediate risk prostate cancer [55]. Approximately half were treated to 36 Gy in 6 Gy fractions, and the others received 4 fractions of 9.5 Gy over 2 days. The 8-year bRFS was 97%. The grade 3 GU toxicity was 5% overall, 24 % grade 2, but this was scored per event, not per patient, and hence the same patient with more than one symptom would be scored multiple times. Late GI toxicity was <1%.

Mount Vernon hospital have published outcomes for a group of men, some with locally advanced prostate cancer [56]. This was a dose escalation study so the first cohort received 34 Gy in 4 fractions over 3 days, the second cohort 36 Gy, then the third cohort received 31.5 Gy in 3 fractions over 2 days. Only 25-31% patients had grade 1 or more toxicity at six months and two patients had grade 3 toxicity.

Aluwini et al, working at Erasmus Medical Centre have reported 166 patients treated with 38 Gy in 4 fractions with 35 months median follow-up [57]. Biochemical control was 97.6% and late G2+ urinary and rectal toxicity was 19.7% and 3.3%, respectively.

6.9 Experience with profound hypofractionation with external beam radiotherapy

The largest 5-year follow-up data for men treated with SBRT has recently been published. King et al report on 1100 men treated with Cyberknife, 65% received 36.25 Gy in 5 fractions or above [11]. Median follow-up is 36 months and biochemical control at 5 years is 95%, 84% and 81% for low, intermediate and high risk patients, respectively. Fourteen percent of this cohort received androgen deprivation therapy (ADT) and no correlation between ADT use and biochemical outcome was noted.

A number of other non-randomised studies have examined SBRT using both Cyberknife and gantry-based systems. These have demonstrated medium term outcomes in keeping with conventionally fractionated treatments, both in terms of efficacy and toxicity [58].

6.10 Extra-capsular extension

There is a theorectical concern that with such conformal isodoses and a sharp dose fall-off, undetected extra-capsular extension could be under-treated. Whilst a preponderance of marginal recurrences is not widely recognised with HDR techniques, which achieve similarly sharp dose fall-offs, this is worthy of further discussion. According to the algorithm proposed by Roach et al. [59] the most advanced patients in this cohort will have a 69% risk of extra-capsular extension (ECE).

However, histopathological studies would suggest that the mean length of extra-capsular extension across all stages is 0.8mm with a median of 0.5mm [60]. A margin of 2.5mm would cover 96% of cancers in this cohort of 376 cases, some of which were Gleason 8 or 9 cancers. In addition, the radial extent of invasion is much smaller for the lower risk prostate cancers, with Gleason <7 cancers extending a median of 0.06mm. It appears likely, therefore that a margin of 1-2 mm would cover almost all possible extracapsular spread in the cohort of this trial.

Another more recent study found slightly more extensive ECE [61]. 371 prostatectomy specimens were analysed from patients receiving surgery between 1987 and 2001. They found that PSA, Gleason score and clinical T score were all correlated with the risk of ECE. They found that low-risk patients had a 19% risk of ECE vs 42% for other groups (both of which are lower than the rates predicted by the Roach equation). The median ECE was 2.4mm but the 90% percentile for distance was 5.0 mm. In addition, for patients with a PSA>10 and a Gleason score of 7 or more, the chance of ECE extending more than 4mm was 20%. Almost all ECE occurred in the posterolateral direction, in the direction of the neurovascular bundles

6.11 Margins for SBRT

For Cyberknife SBRT, most of the larger series have used a PTV margin of 5mm around the prostate/SVs, except for posteriorly where a 3mm margin has been used [11, 62-64]. Biochemical efficacy and side effect profiles have been acceptable in these series, suggesting that this margin is sufficient to cover disease without unacceptable dose delivery to normal tissues. The Cyberknife system monitors and corrects for intra-fraction motion every 30-60 seconds, which means that the dosimetric impact of motion is likely to be small.

For systems such as Calypso electromagnetic beacons which track intrafraction motion continuously, similar margins can be used. For systems where continuous intra-fraction motion monitoring is not possible, margins have to be considered carefully.

Several studies, largely using Calypso monitoring have demonstrated that prostate motion over several minutes is largely within 3mm of initial position. Curtis et al observed prostate motion in 31 patients over 1045 fractions. Over a mean fraction length of 7 minutes and 21 seconds, margins of 3mm would result in geometric coverage of the PTV 93.1% of the time and 5mm margins would ensure geometric coverage 99.4% of the time [65]. Within 180 seconds of set-up, the prostate remains within 3mm of starting position for 95.5% of the time. Bittner et al. examined prostate motion in the prone position, which may not be predictive of motion in the supine position, but found that over a mean tracking time of 12 minutes, the centroid of the transponders was ≥4mm for 4.5% of the time [66]. Langen et al. used Calypso to monitor prostate motion in 17 patients over 550 fractions. They found that the prostate was displaced >3mm and >5mm for 13.6% and 3.3% of the time respectively over a mean treatment time of 10 minutes. It seems likely therefore that margins between 3 and 5mm would be sufficient to cover intrafraction prostate motion for 3+ minutes.

6.12 Do patients in this study need androgen deprivation?

Roach et al. conducted a meta-analysis of 2742 men enrolled into RTOG trials of radiotherapy vs radiotherapy plus hormonal therapy. No evidence could be found that those with early stages of disease, such as those eligible for this study, have any benefit from adjuvant hormonal therapy [67].

D'Amico et al. randomised 206 men with clinically localized prostate cancer to radiotherapy with 70 Gy with or without ADT. A significant improvement in overall and disease-specific survival was found. However, nearly half the men in this study had Gleason 4+3 or higher disease and 12-13% had a PSA of >20 ng/mL [68]. In addition, a dose of 70 Gy would now be considered inadequate.

Denham et al. report the results of the RTOG 9601 trial which again randomised to radiotherapy with or without hormonal therapy or either 3 or 6 months duration [69]. Radiation dose in this trial was 66 Gy. The trial showed a significant improvement in disease-free survival with the addition of hormonal therapy, however over 80% of patients were in the high risk group and once again the dose was low.

A large study of over 1200 men treated across three institutions with EBRT and HDR boost [70]. This showed no benefit in the addition of ADT on overall survival, cause-specific survival and bRFS. In addition the use of ADT was associated with an increase in the development of metastases and of cancer-specific death rates, although clearly this was confounded by the discretionary nature of ADT in this scenario.

Nearly two thousand men were entered into a trial which randomised to short-course hormones with radiotherapy or radiotherapy alone, given to a dose of 66 Gy to the prostate [71]. This showed an improvement in disease-specific and overall survival, but subgroup analysis showed this only to be the case for intermediate risk patients.

The studies discussed above included men with a mixture of prostate cancer stages, used doses of radiotherapy now considered suboptimal, and were largely conducted in the pre-IMRT and IGRT era. There is, therefore, no convincing evidence that men with low and intermediate prostate cancer benefit from the addition of ADT to radiotherapy. Indeed there is some evidence that the addition of ADT may increase the α/β ratio of prostate cancer cells, thereby reducing the predicted therapeutic benefit of hypofractionation [72]. The NCCN guidelines for prostate cancer state that men with low risk prostate cancer should not be given ADT (NCCN 2014) and the EAU 2012 guidelines state that the role of hormones in high dose (>72 Gy) irradiation is unclear for intermediate risk disease [39, 73].

A recently published study has analysed the RTOG 9406 trial data and found that in this cohort of men who received a mean dose of 78.5 Gy, the addition of hormonal therapy was of no benefit to those in any risk group, although there was a non-significant trend to improved bRFS in the high risk group [74]. This suggests that adjuvant hormonal therapy may not be the standard of care for low- and intermediate-risk patients.

Recent retrospective analyses have tried to delineate the subgroup of intermediate-risk patients who may benefit from hormonal therapy. Zumsteg et al. found that intermediate risk patients treated with >81 Gy and short-course hormonal therapy had superior biochemical control and prostate cancer specific mortality compared with those treated with radiation alone. This contrasts with the results of two other studies which showed no significant improvement in biochemical outcomes with androgen deprivation in intermediate risk disease [74, 75].

6.13 Radiobiological rationale for study doses

Table 1: Summary of BED doses for conventional and hypofractionated radiotherapy

	BED if α/β ratio = 5	BED if α/β ratio = 4	BED if α/β ratio = 3	BED if α/β ratio = 2	BED if α/β ratio = 1.5
78 Gy in 39 fractions	109 Gy	117 Gy	130 Gy	156 Gy	182 Gy
62 Gy in 20 fractions	100 Gy	110 Gy	126 Gy	158 Gy	190 Gy

36.25 Gy in 5 fractions	88 Gy	101 Gy	123Gy	168 Gy	211 Gy
40 Gy in 5 fractions	104 Gy	120 Gy	147 Gy	200 Gy	253 Gy

In summary, it is likely that the therapeutic ratio can be improved by hypofractionation and, whilst moderate hypofractionation is likely to become a new standard of care, the next question is whether more profound hypofractionation can improve outcomes for men with prostate cancer.

7 Study Objectives

7.1 Primary Objectives:

<u>In PACE A:</u>

7.1.1 To determine whether there is improved quality of life following prostate SBRT compared with prostatectomy two years from completion of trial treatment.

In PACE B:

7.1.2 To determine whether prostate SBRT is non-inferior to conventional radiotherapy for freedom from biochemical/clinical failure in low/ intermediate risk prostate cancer.

7.2 Secondary Objective(s):

In PACE A:

7.2.1 To determine whether prostate SBRT is non-inferior to surgery for freedom from biochemical/clinical failure in low/ intermediate risk prostate cancer.

In PACE A and PACE B:

7.2.2 To determine the relative benefits of surgery, conventional radiotherapy and prostate SBRT in terms of local failure, distant failure, disease-free survival, disease-specific survival, overall survival, toxicity, quality of life in generic and organ specific domains.

8 Study Design

PACE is a multicentre, international phase 3 randomised controlled study comprising two parallel randomisations with a common experimental arm.

In PACE A, patients considered candidates for surgery, agreed by both the physician and patient, are randomised to either prostatectomy (control) or prostate SBRT delivered to a dose of 36.25 Gy in 5 fractions.

In PACE B, nonsurgical candidates or patients who refuse surgery are randomised to either conventional radiotherapy (control) or prostate SBRT (36.25 Gy in 5 fractions) . From version 7 of the protocol, centres will be asked to select a control arm of either 78 Gy in 39 fractions or 62 Gy in 20 fractions and this will be used for all PACE B patients allocated to the control group Centres will be permitted to change their control arm from 78 Gy in 39 fractions to 62 Gy in 20 fractions at

any point after version 7 of the protocol is implemented but this schedule must then be used for all patients subsequently entered at that centre.

Randomisation will be stratified by randomising centre (and hence by choice of control group fractionation) and by risk group as defined by National Comprehensive Cancer Network (see Appendix 2).

Low Risk:

- Clinical stage T1c – T2a
- PSA <10 ng/ml
- Gleason score ≤ 6

Intermediate Risk includes the presence of any of the following:

- Clinical stage T2b-T2c
- PSA 10 – 20 ng/ml
- Gleason score 3+4

8.1 Primary endpoint

In PACE A:
Co-primary endpoints:

(1) Urinary incontinence (number of absorbent pads required per day to control leakage) measured by The Expanded Prostate Cancer Index (EPIC) questionnaire.
(2) Bowel bother summary score from the EPIC questionnaire.

The primary time point of interest is two years from completion of trial treatment.

In PACE B:
Freedom from biochemical (Phoenix definition for SBRT and conventional radiotherapy arms, >0.2 ng/ml for surgical arm) or clinical (commencement of androgen deprivation therapy) failure. The primary timepoint of interest is 5 years from randomisation.

8.2 Secondary endpoints

In PACE A:

8.2.1 Freedom from biochemical (Phoenix definition for SBRT and conventional radiotherapy arms, >0.2 ng/ml for surgical arm) or clinical (commencement of androgen deprivation therapy) failure. The primary timepoint of interest is 5 years from randomisation.

In PACE A and B:

8.2.2 Clinician reported acute toxicity, assessed using CTCAEv4.03, RTOG (for SBRT and conventional radiotherapy only) and the Clavien scale (to assess acute post surgical complications for surgical patients only).

8.2.3 Clinician reported late toxicity, assessed using CTCAEv4.03 and RTOG (for SBRT and conventional radiotherapy only).

8.2.4 Patient reported outcomes and quality of life assessment for all treatment patients: Assessed using International Index of Erectile Function-5 (IIEF-5)[29], International Prostate Symptom Score (IPSS)[76], Vaizey score[77], Expanded Prostate Index Composite-26 (EPIC-26)[30].

8.2.5 Disease-specific and overall survival.

8.2.6 Progression-free survival– radiographic, clinical or biochemical evidence of local or distant failure.

8.2.7 Commencement of androgen deprivation therapy (LHRH analogues, anti-androgens, orchidectomy).

8.3 Definition of biochemical failure

All biochemical failures need to be confirmed with a second PSA meeting the criteria for failure. In addition, it is now recognised that after SBRT a benign PSA bounce is seen in up to 20% of patients, usually within the first 2 years [11, 78, 79]. A benign PSA bounce may also occur with conventional radiotherapy. In some cases the magnitude of the bounce is high enough for the patient to be incorrectly classified as a PSA failure. To prevent this, for patients receiving SBRT or conventional radiotherapy, PSA failure before 24 months will require 3 consecutive rises in PSA resulting in a clinical diagnosis of failure, or commencement of further treatment (eg androgen deprivation therapy). After 24 months, the definition of PSA failure for patients receiving radiotherapy will revert to the Phoenix definition described above (i.e. nadir+2 ng/ml).

9 Patient selection

Patients suitable for surgery and willing to consider a surgical treatment will be invited to enter PACE A. Those who are not suitable for surgery or are unwilling to consider an operation will be invited to enter PACE B.

9.1 Inclusion Criteria: All of the following criteria are mandatory for inclusion:

9.1.1 Histological confirmation of prostate adenocarcinoma with a minimum of 10 biopsy cores taken within 18 months of randomisation.

9.1.1.1 This requirement for biopsy within 18 months of randomisation may be omitted (unless clinically indicated) if the patient has become a candidate for radical treatment (e.g. due to patient choice or PSA/MRI progression) while being followed up in an active surveillance programme. The patient's most recent biopsy must satisfy all other relevant PACE trial eligibility criteria. In addition the patient must have a recent MRI confirming organ confined disease, within 8 weeks of the decision to treat. Patients progressing on Active Surveillance (AS) will be considered as having intermediate risk disease, and treated accordingly.

9.1.2 Gleason score ≤ 3+4

9.1.3 Men aged ≥18 years

9.1.4 Clinical and/or MRI stage T1c –T2c, N0-X, M0-X (TNM 6th Edition [80], See Appendix 1)

9.1.5 PSA ≤ 20 ng/ml

9.1.6 Pre-enrollment PSA must be completed within 60 days of randomisation

9.1.7 Patients belonging in one of the following risk groups according to the National Comprehensive Cancer Network (www.nccn.org): See Appendix 2

- Low risk: Clinical stage T1-T2a and Gleason ≤ 6 and PSA < 10 ng/ml, or
- Intermediate risk includes any one of the following:
  - Clinical stage T2b orT2c
  - PSA 10-20 ng/ml or
  - Gleason 3+4

9.1.8 WHO performance status 0 – 2

9.1.9 Ability of the research subject to understand and the willingness to sign a written informed consent document

9.1.10 Ability/willingness to comply with the patient reported outcome questionnaires schedule throughout the study.

9.2 Exclusion criteria: One of the following criteria is sufficient for exclusion:

9.2.1 Clinical stage T3 or greater

9.2.2 Gleason score ≥ 4 + 3

9.2.3 High risk disease defined by National Comprehensive Cancer Network (www.nccn.org): See Appendix 2

9.2.4 Previous malignancy within the last 2 years (except basal cell carcinoma or squamous cell carcinoma of the skin), or if previous malignancy is expected to significantly compromise 5 year survival

9.2.5 Prior pelvic radiotherapy

9.2.6 Prior androgen deprivation therapy (including LHRH agonists and antagonists and anti-androgens)

9.2.7 Any prior active treatment for prostate cancer. Patients previously on active surveillance are eligible if they continue to meet all other eligibility criteria.

9.2.8 Life expectancy <5 years

9.2.9 Bilateral hip prostheses or any other implants/hardware that would introduce substantial CT artifacts

9.2.10 Medical conditions likely to make radiotherapy inadvisable eg inflammatory bowel disease, significant urinary symptoms

9.2.11 For patients having fiducials inserted. Anticoagulation with warfarin/ bleeding tendency making fiducial placement or surgery unsafe in the opinion of the clinician (see section 11, Treatment).

9.2.12 Participation in another concurrent treatment protocol for prostate cancer

10 Study assessments and randomisation procedures

Patients will be screened for eligibility based on the inclusion/exclusion criteria.

10.1 Pre-treatment evaluations required for eligibility. The following evaluations should be performed within 6 weeks preceding randomisation unless otherwise indicated:

10.1.1 Complete history and physical examination (DRE if clinically indicated)

10.1.2 Assessment of fitness for anaesthetic by surgeon/ anaesthetist/ research nurse if being considered for the surgery vs prostate SBRT randomisation.

10.1.3 Assessment of performance status (recorded using WHO scale)

10.1.4 Pathological confirmation of adenocarcinoma of the prostate with Gleason scoring within 18 months of randomisation (unless on active surveillance and biopsy not clinically indicated, see Section 9 Patient selection).

10.1.5 Local staging assessments may include digital rectal exam (DRE) and transrectal ultrasound (TRUS). It is recommended that MRI of the pelvis be used for staging purposes. These assessments do not have to be done within 6 weeks preceeding randomisation.

10.1.6 PSA to be checked within 60 days of randomisation.

10.1.7 Within 6 weeks prior to the start of treatment testosterone will be measured and baseline symptoms will be assessed using Common Toxicity Criteria for Adverse Event Reporting (CTCAE) version 4.03 and RTOG bladder and bowel toxicity scoring (for patients randomised to receive radiotherapy only).

10.1.8 Patient should be able to complete patient questionnaires:

10.1.8.1 International Prostate Symptom Score (IPSS)

10.1.8.2 International Index for Erectile Function-5 (IIEF-5)

10.1.8.3 The Expanded Prostate Index Composite-26 (EPIC-26) Short Form questionnaire

10.1.8.4 Vaizey Incontinence Questionnaire

10.2 Informed Consent Process

10.2.1 The protocol and the informed consent must have local ethics committee/IRB approval prior to research activity. The site Principal Investigator (PI) is responsible for ensuring that only a current ethics committee/IRB approved consent form designed specifically for the study is appropriately signed.

10.2.2 The written consent document should embody, in language understandable to the participant, all the elements necessary for legally informed consent. The trial will be conducted in English.

10.2.3 The site PI is responsible for ensuring that proper informed consent has been obtained from the research subject before any study/research activity is conducted. The site PI can designate authorised members of the research team to obtain the informed consent.

10.2.4 The site PI is ultimately responsible for determining whether a subject has the capacity to consent. As part of the consent process, the subject's questions must be answered prior to consent being given and throughout the study. The subject should be asked if there are any questions prior to consent being obtained.

10.2.5 When giving the consent, the subject needs to verbalize understanding, and sign and date the last page of the Consent Form along with the investigator or designee obtaining consent.

10.2.6 The signed consent will be filed in the patient's research study chart or record and the investigator site file. In addition, the subject will receive a copy of the consent form.

10.2.7 The sponsor or their delegate may need to review all consent documents if deemed necessary.

10.3 Randomisation procedures

Randomisation procedures are the same for PACE A and PACE B.

Patients must be randomised centrally by the trials unit (ICR-CTSU) before trial treatment can commence. UK patients should be randomised by telephoning ICR-CTSU on:

020 8643 7150
09.00-17.00 (UK time) Monday to Friday

For non UK patients, randomisation outside of UK office hours, should be requested by faxing the ICR-CTSU on:

+44 (0) 20 8770 7876
09.00-17.00 (UK time) Monday to Friday

Further details of randomisation procedures for non-UK patients will be provided within the international site agreements.

An eligibility and randomisation checklist must be completed prior to randomisation.

The following information will be required at randomisation:

- Name of treating and recruiting hospital, consultant and person randomising patient
- Confirmation that patient has given written informed consent for trial and for any sub-studies;
- Confirmation that patient is eligible for the trial by completion of the eligibility checklist
- Patient's full name, risk group, hospital number, date of birth
- Patient's postcode and NHS/CHI number (for UK patients only)

The caller will be given the patient's unique randomisation number (Trial ID) and treatment allocation.

ICR-CTSU will send written confirmation of trial entry to the data management contact at the recruiting centre.

Treatment allocation will be 1:1 for surgery vs prostate SBRT and 1:1 for conventional radiotherapy vs prostate SBRT. Treatment allocation will use computer generated random permuted blocks. Randomisation will be stratified by randomising centre and risk group

10.4 Evaluation during and following treatment.

10.4.1 Patients will be assessed regularly (as per Table 2 (PACE A) or Table 3 (PACE B)) during treatment and after completion of radiotherapy or from the date of surgery. For conventional radiotherapy, toxicity assessment will be recorded at weeks 2, 4, 6 and 8 during treatment (dependent on duration of treatment – see Table 3). For SBRT, toxicities will be recorded on the day the last fraction is delivered. For surgery, toxicities will be recorded on last day of hospitalisation. Clavien toxicity score will be taken on the last day of hospitalisation, week 2 and 4 for surgery patients.

10.4.2 For the first 12 weeks after treatment completion, toxicity assessments will be recorded at each clinic attendance for all patients and then 3 monthly for the first 2 years, 6 monthly to years 5 and annually to year 10. At 4 weeks, 12 weeks, 6, 9, and 12 months following treatment and yearly thereafter (until year 5) the following will be recorded: EPIC-26, IIEF-5 (not recorded at 4 weeks and 9 months), IPSS and Vaizey. There are two additional assessments of IPSS at week 2 and week 8 following treatment. PSA will be recorded at 12 weeks, 6, 9, and 12 months following treatment and yearly thereafter. Quality of life booklets should be handed out in clinic at all relevant time points, and completed by the patient. Every effort should be made to ensure that the questionnaires are completed. Please aim to ensure that all questions and all pages have been completed by the patient when the booklet is handed in; see section 10.8 for full details regarding administration of the quality of life booklets.

10.4.3 At all timepoints, toxicity assessement will record the maximal toxicity since the last toxicity assessment.

10.4.4 Thereafter patients will be seen every 3 months for the first 2 years, every 6 months to 5 years and annually to year 10. Three monthly follow up for the first two years may be done as a telephone consultation, at the discretion of the treating clinician.

10.4.5 Follow-up visit windows:

- During treatment: ± 3 days
- Week 2 and Week 4 visit: ± 3 days
- Week 8 and Week 12 visit: ± 1 week
- Month 6 and Month 9: ± 2 weeks
- Month 12 and thereafter: ± 4 weeks

10.5 Participation in other clinical trials

Patients who fulfil the eligibility criteria will be given the opportunity to participate in PACE even if they have participated in other clinical trials prior to recruitment. Participation in non-interventional studies (eg UKGCPS study www.icr.ac.uk/ukgpcs or RAPPER study), is permitted.

Participation in other clinical trials whilst participating in PACE will be considered on a trial by trial basis by the PACE Trial Management Group.

10.6 Tissue donation for translational studies

Patients will be asked at the time of consent whether they will agree to donate their biomaterials from diagnostic tissue samples for future translational research. This will be optional. The pathology number and storing hospital of donated samples will be recorded to facilitate retrospective collection of tissue for future translational studies. Patients provided with earlier versions of the patient information sheet (i.e. prior to v5 dated 5th August 2014) recruited prior to the tissue donation amendment may be invited to provide further consent for tissue donation retrospectively.

Translational research will not form part of the PACE study itself, but it is anticipated that data from PACE outcomes, along with tissues samples donated, may be used in future research studies.

10.7 Data sharing

Combining data from many clinical trials may help to further our knowledge of cancer treatment. In view of this, patients will be asked to consent to the sharing of their anonymised data with other legitimate researchers, in order to facilitate this. This will be optional. Patients provided with earlier versions of the patient information sheet (i.e. recruited prior to amendment 6) will be invited to provide further consent for sharing of anonymised data (this will only affect patients recruited at the Royal Marsden Hospital and Mount Vernon Hospital).

Table 2: PACE A (Surgery vs. SBRT) schedule of assessments

	Pre-randomisation	Pre-treatment	Last day of hospitalisation (surgery pts) or last fraction (SBRT pts)	Follow up post completion of treatment						
**Assessment**				Week 2[a]	Week 4	Week 8[a]	Week 12	Month 6	Month 9	Month 12 and thereafter
Clinical history	x									
Physical Examination (DRE if clinically indicated)	x									
ASA score (for patients randomised to surgery only)	x									
PSA	x						x	x	x	x
Testosterone		x								
MRI pelvis [b]	x									
Toxicity assessment (CTCAE, RTOG[c]bladder and bowel toxicity)		x	x	x	x	x	x	x	x	x
Clavien toxicity score (for patients randomised to surgery only)			x	x	x					
QOL: EPIC-26, IPSS, IIEF-5, Vaizey.	x			x[d]	x[e]	x[d]	x	x	x[e]	x yearly to year 5

[a]can be telephone consultation at the discretion of the treating clinician

[b] MRI is recommended for staging purposes. MRI is strongly recommended for radiotherapy planning purposes.

[c] RTOG assessment not required for patients having surgery.

[d] IPSS ONLY required at week 2 and week 8.

[e] IIEF-5 should NOT be reported at this time point

Table 3: PACE B (Conventional Radiotherapy vs SBRT) schedule of assessments

	Pre-randomisation	Pre-treatment	During treatment for conventional radiotherapy only[e]				Last frxn (SBRT only)	Follow up post completion of treatment						
Assessment			Wk 2	Wk 4	Wk 6	Wk 8		Wk 2[a]	Wk 4	Wk 8[a]	Wk 12	Mo 6	Mo 9	Mo 12 & there after
Clinical history	X													
Physical Examination (DRE if clinically indicated)	X													
PSA	X										x	x	x	x
Testosterone		x												
MRI pelvis [b]	X													
Toxicity assessment (CTCAE, RTOG bladder and bowel toxicity)		x	RTOG only	RTOG only	RTOG only	RTOG only	X	x	x	x	x	x	x	x
QOL: EPIC-26, IPSS, IIEF-5, Vaizey	X							x[c]	x[d]	x[c]	x	x	x[d]	x yearly to year 5.

[a] can be telephone consultation at the discretion of the treating clinician

[b] MRI is recommended for staging purposes. MRI imaging is strongly recommended for radiotherapy planning purposes.

[c] IPSS ONLY required at week 2 and week 8.

[d] IIEF-5 should NOT be reported at this time point

[e] If patients are being treated with 62 Gy in 20 fractions, week 2 and 4 only required

Additional follow-up and investigations are permitted as per usual institutional policy.

Patient Pathway

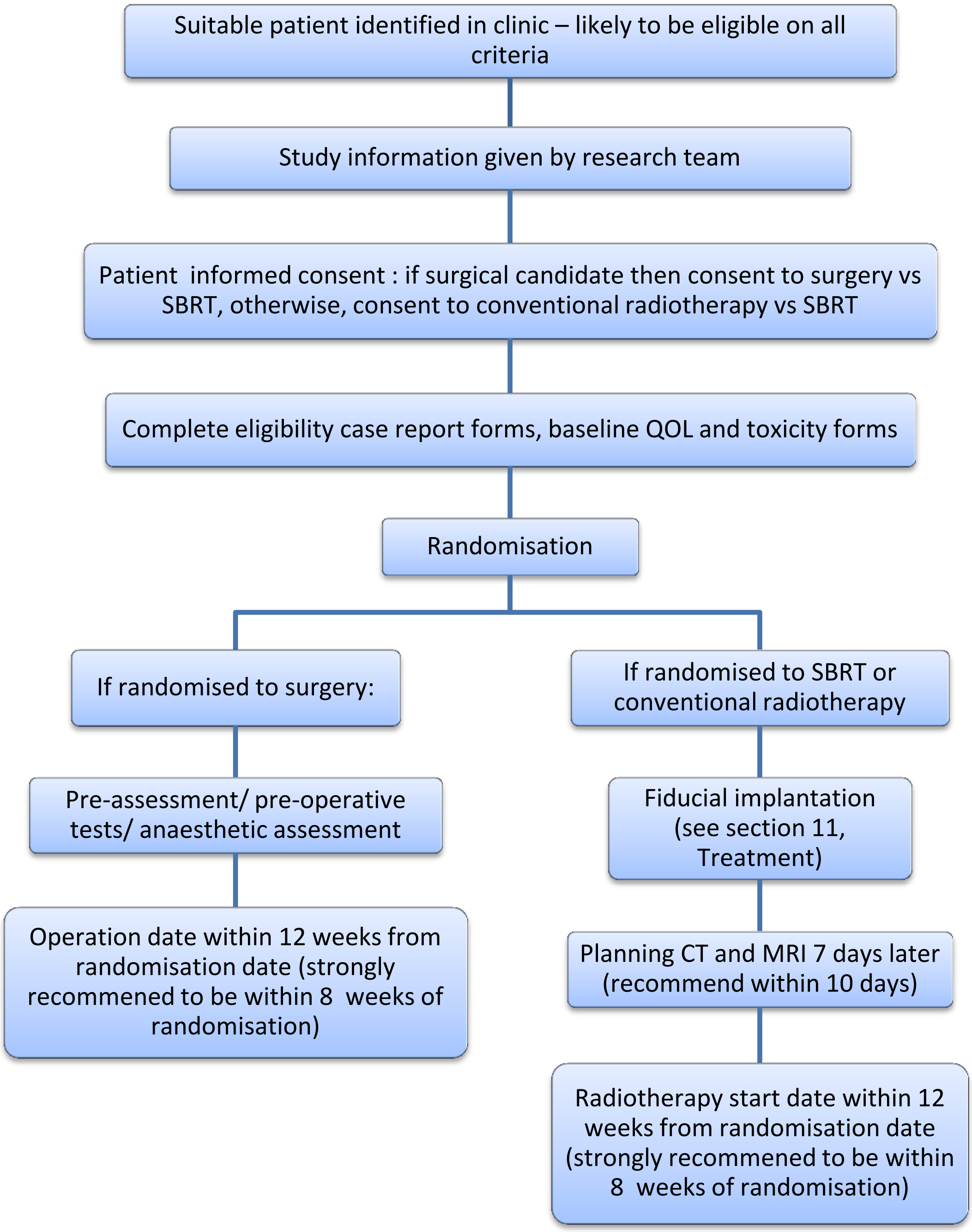

10.8 Instructions regarding administration of patient reported study questionnaires

These instructions are for the study coordinator or research nurse administering the questionnaires:

- All QL booklets will be administered by the centre in accordance with Table 2 and 3 of the PACE study protocol. The target timeframe for completion of follow up questionnaires will be +/- two weeks of the scheduled follow-up assessment.
- If possible the patient should be taken to a quiet area where he can complete the questionnaires prior to the clinic visit.
- Enough time should be allowed for the patient to complete the questionnaires.
- The patient should be encouraged to complete every item in order without skipping any. If the patient feels that a given question does not apply to him he should circle the response that is most applicable: no problem, not at all, none at the time, rarely or never.
- The questionnaires must be completed by the patient alone without coaching or suggestions by health care personnel or anyone else. The study staff might provide clarification without suggesting answers or discussing answers.
- The study staff will collect the questionnaires, checking for completeness. If a question or questionnaire has not been completed and the patient states he does not wish to answer the question or complete the questionnaire this can be documented on the questionnaire by the study coordinator/research nurse.
- If the patient does not come to the clinic the questionnaires can be posted to the patient by the site study staff, including a self addressed envelope so that the questionnaires can be returned to clinic. The patient will be reminded to complete and return the questionnaires in a timely manner during the phone follow-up.
- Completed patient questionnaires should be returned to the PACE Trial Manager at the ICR-CTSU.

10.9 Withdrawal of patients from study

During the course of the study, it is possible that patients will be withdrawn from the study. Factors leading to patient withdrawal may include, but are not limited to, the following:

- Patient withdrawal: A patient may voluntarily withdraw their consent from the study at any time without affecting their future medical treatment or benefits.

- Investigator termination: the investigator may terminate the patient's participation without regard to the patient's consent if the investigator believes it is medically necessary (e.g. if the patient becomes cognitively or physically incapacitated), the patient is not following the protocol, the Sponsor has stopped the study or other administrative reasons.

- Sponsor discontinuation: The sponsor may discontinue the study upon ethics committee request, or for safety issues. The sponsor shall promptly notify all investigators, and the applicable authorities in the European Union should the study be discontinued or terminated prematurely. Should the study be terminated prematurely, all treatment related records and all due CRFs would be collected by the sponsor.

- Patient lost to follow-up: A patient will be considered lost to follow-up with documentation of three unsuccessful attempts by the Investigator or his/her designee to

contact a patient or next of kin. For UK patients, if necessary, NHS and national health registration data will be used to obtain survival outcomes.

Patients who do not receive any or all of their allocated treatment for any reason should be treated at the discretion of their clinician. Unless the patient requests otherwise, all eCRFs, including long term follow-up, should be completed regardless of treatment actually received. A protocol deviation form should be completed to record details of deviation from treatment allocation.

Patients are asked prior to randomisation to consent to basic follow up information being provided from routine clinic visits should they withdraw from the study (see patient information sheet and consent form). Patients are however free to reverse their decision at any time without giving a reason. A study deviation form should be completed for any patient who withdraws consent for information to be provided on eCRFs or for attending study follow up visits.

Should a patient become cognitively or physically incapacitated at any point during the study they will be withdrawn for their own protection. If this were to happen during the course of the patient's radiotherapy their treatment should be reviewed as a clinical decision by the Principal Investigator at their centre. No further study procedures will be carried out and no further data will be collected on behalf of the study. Any data already collected about such patients will be fully anonymised. A study deviation form should be completed for any patient withdrawn from the study for this reason.

In the very rare event that a patient requests that their data is removed from the study entirely, the implications of this should be discussed with the patient first to ensure that this is their intent and, if confirmed, ICR-CTSU should be notified in writing. If this request is received after results have been published the course of action will be agreed between the Sponsor and independent Trial Steering Committee/Independent Data Monitoring and Steering Committee.

10.10 Compensation

Patients will not be paid. Patients, their health authorities and/or their insurance companies will be responsible for the cost of all procedures and treatments under this protocol.

11 Treatment

11.1 Conventional Radiotherapy and SBRT Treatment Planning

11.1.1 Fiducial Placement: it is stongly recommended that all patients randomised to radiotherapy have fiducial markers (measuring 3-5mm) implanted for image guidance. Fiducial markers should be visible on CT and MRI imaging to allow image guidance and MRI/CT fusion. At least three fiducial markers will be placed under transrectal ultrasound guidance, using either transperineal or transrectal approach. Antibiotic cover with oral ciprofloxacin or equivalent and metronidazole per rectum, or equivalent, should be administered if fiducial placement is done transrectally. The physician will place seeds such that they are visible (and not superimposed) on orthogonal imaging (where used) and ideally are separated by 2 cm or more. Fiducials are usually placed as an outpatient procedure; at least three seeds must be usable for tracking translation and rotation during treatment. The use of one paired fiducial and two free fiducials (four in total) is recommended for Cyberknife SBRT treatment.

11.1.2 Treatment Plan Imaging

11.1.2.1 To allow fiducial stabilization and resolution of swelling, planning studies will be imaged at least 7 days after fiducial placement. Patients will be scanned supine with arms across chest using an Alpha Cradle, vacbag or similar immobilization device, as needed. Knee and ankle supports may be used. Positioning and immobilisation should be as similar as possible during the planning MRI.

11.1.2.2 Bowel preparation: we strongly advise bowel preparation to reduce rectal diameter for all patients receiving radiotherapy. Aim for a maximum rectal AP diameter of 4cm, measured at the mid point of the prostate. We suggest daily enemas for 2 days prior to, and on the day of CT planning. We suggest patients should restart enemas 2 days prior to starting radiotherapy. SBRT patients are suggested to have an enema on each day of treatment. Conventional radiotherapy patients are suggested to have an enema daily for the first 2 weeks of treatment, unless they develop diarrhoea.

11.1.2.3 It is recommended that patients have a partially filled bladder during imaging and treatment delivery: patients should be asked to empty their bladder and then drink enough water (eg 325 mls) to ensure a reasonably filled bladder on the planning scan and before each fraction of radiotherapy. It is advised that the bladder should be filled to at least 150mls to proceed with planning. However, this may not always be possible, and planning may proceed if agreed with the site Principal Investigator (PI).

11.1.2.4 CT scans will be taken for treatment planning. CT slices will be 1 – 1.5mm, with 200-300 slices taken centered approximately at the prostate. For Cyberknife SBRT scans will extend at least 15 cm above and below the level of the prostate, including the testes so that these can be used as a blocking structure. For gantry-based SBRT and IMRT,

scans should extend from L3/L4 interverterbral space to 2cm below ischial tuberosities.

11.1.2.5 It is strongly recommended that all patients undergo MRI imaging for radiotherapy planning purposes to determine the anatomical borders of the prostate, and if possible, the urethra. The MRI will be fused to the treatment planning CT. It is recommended that MRI/CT fusion be done on implanted fiducials. No endorectal coil is allowed.

11.2 Evaluated Structures

11.2.1 The Clinical Target Volume (CTV):

When using MRI-fusion images for voluming, it is acknowledged that these tend to be less accurate more superiorly, particularly at the level of the seminal vesicles. Therefore we recommend using MRI fusion for voluming the prostate and prostate/rectum interface, but where there is a discrepancy the CT anatomy should be used. All other structures should be outlined on CT.

For the purposes of this study, the CTV shall be defined as follows:

All patients:<br>Low risk: CTV = prostate only (as defined on MRI planning scan where available)<br>Intermediate risk: CTV = prostate plus proximal 1cm of seminal vesicles from insertion point in the superior-inferior plane. This should include the middle ½ to ⅔ of seminal vesicle width (i.e. not the tips). Please contact the QA team for example contours.

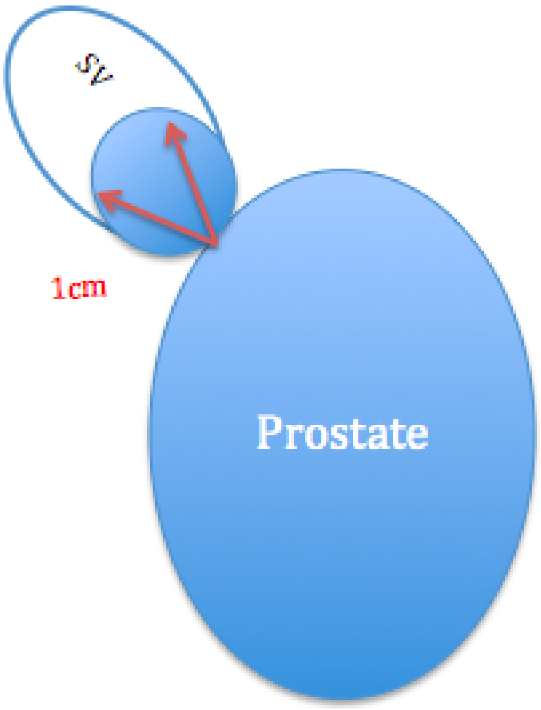

Figure 1: Schematic illustration of the seminal vesicle inclusion for PACE ("CTV" shown in blue)

11.2.2 The Planning Treatment Volume (PTV):

The CTV to PTV margins are different for prostate SBRT and conventional radiotherapy.

11.2.2.1 For conventional radiotherapy, margins will depend on the department's treatment delivery accuracy.

PTV margin for conventional radiotherapy:<br>PTV= CTV+ 5-9mm, except 3-7mm posteriorly

11.2.2.2 For prostate SBRT the PTV is defined as the CTV plus 4-5mm, except posteriorly where the prostate abuts the rectum, where a 3-5 mm margin will be applied.

PTV margins for SBRT (36.25 Gy in 5 fractions)<br>PTV= CTV+ 4-5mm/ 3-5mm posteriorly

11.2.2.3 Planning volumes will be outlined and reported in line with ICRU 83 "Prescribing, recording and reporting photon-beam intensity modulated radiotherapy (IMRT)" where relevant.

11.2.3 Organs at Risk (OAR)

The following OAR will be contoured: these are given in reducing order of priority for planning constraints.

11.2.3.1 <u>Rectum</u>: defined as a solid structure, including the lumen and rectal wall, extending from the anus to the rectosigmoid junction.

11.2.3.2 <u>Bladder</u>: defined as a solid structure including the bladder wall and lumen.

11.2.3.3 <u>Urethra</u> if visible (prostate SBRT only): the prostatic urethra is defined as the lumen-mucosal interface, extending from bladder neck to the membranous urethra.

11.2.3.4 <u>Penile bulb</u>: the portion of the bulbous spongiosum that lies inferior to the urogenital diaphragm.

11.2.3.5 <u>Femoral heads</u>: Femoral heads are to be outlined from their most cranial aspect to the bottom of the curvature of the femoral head (ie exclude the femoral neck)

11.2.3.6 <u>Bowel</u>: Above rectum, within 15cm of PTV for Cyberknife SBRT and within 4cm PTV for gantry-based SBRT and IMRT. Bowel may be outlined as a 'bowel bag'.

11.2.3.7 <u>Testes</u>: For Cyberknife SBRT, beams should not be allowed to traverse the testes, due to the effects on hormone production and subsequent

confusion of biochemical outcomes [81]. The bilateral testes should therefore be used as a 'blocking structure'.

11.2.4 Structured naming convention for volumes

As an NCRN radiotherapy trial, the PACE study uses a standardised naming convention [82]. This will avoid ambiguity and facilitate analysis of radiotherapy plan data. This convention is detailed in table 3.5.

Table 3.5: Structure naming convention for PACE

Volume	Naming convention
Conventional treatment volumes	
Clinical target volume: prostate +/- seminal vesicles	CTVp or CTVpsv
Planning target volume (receives 78 Gy or 62 Gy)	PTV_7800 or PTV_6200
SBRT treatment volumes	
Clinical target volume: prostate +/- seminal vesicles (receives 40 Gy)	CTVp_4000 or CTVpsv_4000
Planning target volume (receives 36.25 Gy)	PTV_3625
Organs at risk	
Rectum	Rectum
Bladder	Bladder
Urethra	Urethra
Left femoral head	FemoralHead_L
Right femoral head	FemoralHead_R
Penile bulb	PenileBulb
Bowel	Bowel

11.3 Dose Specifications: (all specified doses are given over the entire course of treatment).

11.3.1 Conventional radiotherapy Dose Specifications:

11.3.1.1 Dose for the conventional arm will be either 78Gy in 39 fractions daily over 8 weeks OR 62Gy in 20 fractions daily over at least 27 days, and delivered using IMRT. The prescription dose shall be the dose to the PTV and the following dose objectives will be met: for 78Gy: D98%≥74.1Gy, D50%=78Gy±1%, D2%≤83.5Gy (aim for D2% <81.9 Gy): for 62 Gy: D98%≥58.9Gy, D50%=62Gy±1%, D2%≤66.3Gy (aim for D2% <65.1Gy). The minimum dose constraint (D98%) may be relaxed where necessary in order to meet the rectum high dose constraint, with limited undercoverage permitted posteriorly where PTV overlaps rectum.

11.3.1.2 Dose specifications for OAR are shown in Table 4.

Table 4: Dose Specifications for Conventional radiotherapy arm

Organ at risk	Dose volume constraints			
	Dose (Gy) for 78Gy/39 fractions	Dose (Gy) for 62Gy/20 fractions)	Maximum Volume (% or cc)	
			Mandatory	Optimal
Rectum	30	24	-	80%
	40	32	-	65%
	50	40	60%	50%
	60	48	50%	35%
	65	52	30%	-
	70	56	25%	15%
	75	60	5%*	3%
Bladder	50	40	50%	-
	60	48	25%	-
	74	59	15%	5%
Femoral Heads	50	40	50%	5%
Bowel	50	40	17cc	-
Penile bulb	50	40	-	50%
	60	48	-	10%

* May require a reduction in posterior PTV margin and/or removal of rectal overlap from PTV during plan optimisation (as for SBRT planning).

11.3.2 Dose specifications for hypofractionated radiotherapy delivered with SBRT:

11.3.2.1 The dose for the SBRT arm will be 36.25 Gy given in 5 fractions over 1-2 weeks (i.e. daily or alternate daily).The prescription dose of 36.25 Gy shall be the dose to the PTV. V36.25 Gy to the PTV shall be greater than or equal to 95%. A secondary dose of 40 Gy should be delivered to the CTV (i.e. the prostate/SVs) such that the CTV V40Gy is greater than or equal to 95%. For Cyberknife planning, the prescription isodose shall be 65-85% of Dmax (or 75-85% if urethra not contoured). For gantry-based SBRT, the following dose objectives should be met with respect to the PTV: D98% ≥ 34.4 Gy, Dmax < 48 Gy, and aim for D2% ≤ 42.8 Gy, where possible. (A planning guide for gantry-based SBRT is available).

11.3.2.2 Dose specifications for OAR for SBRT are shown in Table 5. Minor and major variations are shown below.

Table 5: Dose Specifications for SBRT (36.25 Gy in 5 fractions)

OAR	Dose constraint
Rectum	V18.1 Gy <50% (i.e. 50% rectum <18.1 Gy) [62, 83]<br>V29 Gy <20 % (i.e less than 20% rectum receiving 29 Gy)<br>V36 Gy <1cc
Bladder	V18.1 Gy <40% [84]<br>V37 Gy <10cc (optimal V37 Gy<5cc)
Prostatic urethra (if visualized)	V42Gy <50% (optimal , not mandatory)
Femoral head	V14.5 Gy <5% [84]
Penile Bulb	V29.5 Gy <50% [85]
Testicular	Blocking structure
Bowel	V18.1 Gy <5cc<br>V30 Gy <1cc

11.3.2.3 Rectum dose variations:

11.3.2.3.1 Minor variation: V36Gy ≥ 1cc, but < 2cc.

11.3.2.3.2 Major variation:V36Gy ≥ 2cc

11.3.2.4 Bladder dose variations:

11.3.2.4.1 Minor variation: V37Gy ≥ 10cc, but < 20cc.

11.3.2.4.2 Major variation: V37Gy ≥ 20cc

11.3.2.5 Target volume variations:

11.3.2.5.1 Minor variation: CTV V40Gy 90-94.9%

11.3.2.5.2 Minor variation PTV: V36.25Gy 90-94.9%

11.3.2.5.3 Major variation CTV: V40Gy<90%

11.3.2.5.4 Major variation PTV: V36.25Gy<90%

11.3.2.6 Investigators shall attempt to keep normal tissue doses and prescription coverage as close to "per protocol" specifications as possible. If all the above "per Protocol" dose-volume criteria cannot be met on a given patient, then normal tissue constraints and target prescriptions may be relaxed to the "minor variation" range as follows: one minor variation in EITHER the primary or secondary dose prescription coverage (e.g. PTV V36.25Gy 90-95% or CTV V40Gy 90-95%) is allowed; two minor variations or one major variation is allowed only with the consent of the site chair.

11.3.2.7 Additional minor variation is allowed for constraints on the rectum and bladder. Major variations on OAR constraints are only allowed with the permission of the site chair. All variations shall be noted.

11.4 Radiotherapy plan data collection

11.4.1 Radiotherapy plan data will be collected (in DICOM format by electronic transfer) for all patients having radiotherapy within the trial. This data will be stored on a secure server by the sponsor.

11.5 Radiotherapy Treatment Delivery and Tracking

11.5.1 All radiotherapy techniques are to be approved in advance by the Chief Investigator and trials QA team.

11.5.2 It is highly recommended that radiotherapy start within 8 weeks of randomisation, but it must start within 12 weeks. Treatment will be given in a single phase over no more than 14 days for SBRT, no more than 61 days for conventional radiotherapy (78 Gy in 39 fractions), and 31 days for moderate hypofractionation (62 Gy in 20 fractions); longer planned treatment durations are to be discussed with the Chief Investigator for approval. In addition, for the 20 fraction treatment schedule overall time of treatment should be at least 27 days (as per CHHiP trial) and, in practice, means that these patients should start treatment on a Wednesday to Friday. Overall treatment duration will be recorded.

11.5.3 All patients will have image-guided radiotherapy, and it is strongly recommended that this is done with fiducial guidance. It is recommended that all patients be set up to fiducial markers prior to treatment and if a significant shift is required (>3mm) the patient should be re-imaged after that shift. In addition, tomographic imaging pre-treatment is encouraged to rule out any significant changes in rectal position or prostate deformation.

11.5.4 At least three fiducials should be identified for each treatment. If fewer than three fiducials can be tracked, then additional fiducials can be placed, and the patient replanned. Where the ability exists rotational corrections should be made.

11.5.5 For SBRT using Cyberknife, patients will have fiducial-based intra-fraction motion corrected during treatment.

11.5.6 For SBRT with gantry based systems, it is anticipated that the majority of centres will use an arc-based IMRT technique, with or without flatterning filter-free delivery. Flattening filter-free delivery should have a beam on time of under 3 minutes, in which case intra-fraction motion control is not mandated. Where beam-on time significantly exceeds 3 minutes, re-imaging should occur between beams/arcs (or at approximately 3-4 minute intervals). It is recommended that the couch is shifted for all displacement but it is mandatory to shift for any displacement ≥ 3mm.

11.5.7 For centres using Calypso beacons or Elekta clarity ultrasound monitoring, prostate motion will be monitored continually and treatment paused (and position corrected) if prostate displacement exceeds 3mm.

11.5.8 For gantry-based SBRT using tomographic imaging (i.e. cone beam CT) without fiducials, centres must demonstrate that they can deliver treatment to the

required accuracy (given the significant prostate motion which may occur during treatment). This will be discussed and agreed on an individual centre basis with the Chief Investigator and trial QA team.

11.6 Surgery Treatment Arm

It is highly recommended that surgery occur within 8 weeks of randomisation, but it must occur within 12 weeks. Radical prostatectomies must be either performed open, laparoscopically or using a robotically assisted laparoscopic approach. Participating surgeons should be performing at least 20 prostatectomies per year [86]. The number of procedures performed per year should be collected on each participating surgeon, as should the positive margin rate. Lymphadenectomy should be performed only when it is the standard practice of the surgeon for that case.

Data will be prospectively recorded on: the Clavien scale of post-operative complications [87], ASA Physical Status Classification System score, and WHO performance status of patients, whether the anastomosis is closed with a continuous or interrupted suture, the number of lymph nodes nodes removed (formal lymphadenectomy is not required in all cases) and 30-day mortality.

Patients will all have deep vein thrombosis/pulmonary embolism (DVT/PE) and antibiotic prophylaxis as per local guidelines. It is anticipated that all abdominal drains will be removed by day 3, and the urinary cathether will be removed before day 14 post-operatively.

12 Adverse event reporting

12.1 Definition of an Adverse Event (AE)

An AE is any untoward medical occurrence in a patient administered a study treatment; the event does not necessarily have a causal relationship with the treatment.

12.2 Definition of a Serious Adverse Event (SAE)

An SAE is any untoward medical occurrence that occurs after the commencement of study treatment and within 30 days of the last day of study treatment and:

- results in death
- is life-threatening
- requires hospitalisation or prolongation of existing inpatients´ hospitalisation
- results in persistent or significant disability or incapacity
- is a congenital anomaly or birth defect

In addition, any RTOG grade 4 events occurring up to 5 years after completion of radiotherapy should be reported according to serious adverse event reporting timelines.

Important adverse events that are not immediately life-threatening or do not result in death or hospitalisation but may jeopardise the subject or may require intervention to prevent one of the other outcomes listed in the definition above, may also be considered serious.

Progression of the indicated disease and death due to progression of the indicated disease are not considered SAEs.

Pregnancy or aid in the conception of a child whilst participating in a trial is not itself considered an SAE but should be followed up for congenital anomalies or birth defects.

Serious Adverse Reaction (SAR)

A serious adverse reaction is an SAE that is suspected as having a causal relationship to the trial treatment, as assessed by the investigator responsible for the care of the patient. A suspected causal relationship is defined as possibly, probably or definitely related (see definitions of causality table).

Definitions of causality

Relationship	Description
Unrelated	There is no evidence of any causal relationship with the trial treatment
Unlikely	There is little evidence to suggest there is a causal relationship (e.g. the event did not occur within a reasonable time after administration of the trial treatment). There is another reasonable explanation for the event (e.g. the patient's clinical condition, other concomitant treatment)
Possible	There is some evidence to suggest a causal relationship (e.g. because the event occurs within a reasonable time after administration of the trial treatment). However, the influence of other factors may have contributed to the event (e.g. the patient's clinical condition, other concomitant treatments)
Probable	There is evidence to suggest a causal relationship, and the influence of other factors is unlikely
Definitely	There is clear evidence to suggest a causal relationship, and other possible contributing factors can be ruled out
Not assessable	There is insufficient or incomplete evidence to make a clinical judgement of the causal relationship.

Related Unexpected Serious Adverse Event
An adverse event that meets the definition of serious and is assessed by the CI or nominative representative as:

- "Related" – that is, it resulted from administration of any of the research procedures, and
- "Unexpected" – that is, the type of event is not listed in the protocol as an expected occurrence (see Appendix 3)

12.3 UK Reporting Adverse Events to ICR-CTSU

For non-UK reporting requirements please see appendix 4.

Any toxicity, sign or symptom that occurs after commencement of study treatment which is not unequivocally due to progression of disease, should be considered an AE.

All AEs must be reported on the relevant toxicity, sign or symptom CRF.

Toxicity evaluation for patients randomised to surgery vs SBRT arm (Study A)

The National Cancer Institute Common Terminology Criteria for Adverse Events (CTCAE) version 4.03 and the RTOG scoring system will be used for toxicity assessment for patients randomised on surgery vs SBRT arm. A copy of the CTCAE Criteria can be downloaded from the CTEP home page (http://evs.nci.nih.gov/ftp1/CTCAE/About.html). The Clavien scale for post-operative complications will be used for surgical patients.

For each AE, the highest grade observed since the last visit should be reported.

Whenever one or more toxicity/sign/symptom corresponds to a disease or a well-defined syndrome only the main disease/syndrome should be reported.

Toxicity evaluation for patients randomised to conventional radiotherapy vs SBRT arm (PACE B)

The National Cancer Institute Common Terminology Criteria for Adverse Events (CTCAE) version 4.03 and the Radiation Therapy Oncology Group (RTOG) Morbidity Scoring Criteria will be used for assessing toxicity for patients randomised on the conventional radiotherapy vs SBRT arm.

For each AE, the highest grade observed since the last visit should be reported.

Whenever one or more toxicity/sign/symptom corresponds to a disease or a well-defined syndrome only the main disease/syndrome should be reported.

12.4 UK Reporting Serious Adverse Events to ICR-CTSU

Any SAE that occurs from the start of study treatment and up to 30 days following the last day of study treatment must be reported.

All SAEs should be reported to ICR-CTSU within 24 hours of the Principal Investigator (or designated representative) becoming aware of the event, by completing the PACE SAE form and faxing to:

The ICR-CTSU safety desk

Fax no: 0208 722 4368

For the attention of the PACE Trial team

As much information as possible, including the Principal Investigator's assessment of causality, must be reported to ICR-CTSU in the first instance. Additional follow up information should be reported as soon as it is available.

All SAE forms must be completed, signed and dated by the Principal Investigator or designated representative.

The Site SAE log should be completed and the SAE form filed in the Site Investigator File.

12.5 Adverse events exempt from expedited reporting

The expected adverse events listed in Appendix 3 are exempt from expedited reporting and should be reported using the appropriate CRF UNLESS they fulfil the protocol definition of an SAE.

12.6 UK Review of Serious Adverse Events

The Chief Investigator (or designated representative) will assess all reported SAEs for causality and expectedness (NB. The Chief Investigator cannot down-grade the Principal Investigator's assessment of causality.)

SAEs assessed as having a causal relationship to study treatment and as being unexpected will undergo expedited reporting to the relevant authorities and all other interested parties by ICR-CTSU.

Sites should respond as soon as possible to requests from the Chief Investigator or designated representative (via ICR-CTSU) for further information that may be required for final assessment of an SAE.

12.7 UK Expedited Reporting of Related Unexpected SAEs

If an SAE is identified as being related and unexpected by the Chief Investigator it will be reported by ICR-CTSU to the main REC, the Sponsor and all other interested parties within 15 days of being notified of the event.

The Principal Investigators at all actively recruiting sites will be informed of any related unexpected SAEs occurring within the trial at appropriate intervals.

12.8 UK Follow up of Serious Adverse Events

SAEs should be followed up until clinical recovery is complete or until the condition has stabilised. SAE outcomes should be reported to ICR-CTSU using the relevant section of the SAE form as soon as the Principal Investigator or designee becomes aware of the outcome.

12.9 UK Annual Safety Reporting

An annual progress report will be provided to the main REC by ICR-CTSU and copied to the Sponsor and the collaborative group in each participating country at the end of the reporting year. This will include data about related unexpected SAEs and whether any safety concerns have arisen during the reporting period.

13 Statistical considerations

All statistical analysis will be conducted by the ICR-CTSU at The Institute of Cancer Research.

13.1 Study Design

This umbrella study consists of two randomised parallel phase III trials with a common experimental arm. . PACE A compares prostatectomy with prostate SBRT and PACE B compares conventional radiotherapy with prostate SBRT. The primary objective of PACE A is to demonstrate superiority of SBRT in terms of patient reported outcomes compared to prostatectomy and PACE B is to demonstrate non-inferiority of SBRT compared to conventional radiotherapy. PACE A and B will be randomised independently and analysed separately.

13.2 PACE A: Surgery vs prostate SBRT randomisation

13.2.1 Sample Size

Following advice from the independent Trial Steering Committee, the primary endpoint and sample size for PACE A was revised due to slower than anticipated recruitment meaning that the original objective of demonstrating non-inferiority of SBRT compared to prostatectomy was not feasible. PACE A now has co-primary endpoints based on patient reported outcomes of urinary incontinence (number of absorbent pads used daily from the EPIC questionnaire) and bowel bother (summary score from the EPIC questionnaire). The aim of the study is to demonstrate superiority of SBRT compared to surgery in terms of both of these important patient reported outcomes The sample size is driven by the comparison of urinary incontinence (any use of urinary pads).

It is estimated that at 2 years from completion of treatment, 15% of surgical patients will be using urinary pads[21]. It is anticipated that 4% of SBRT patients will use urinary pads. Assuming a 5% two-sided alpha and 80% power, 111 patients are required in each treatment group to detect an 11% difference between groups. To allow for 5% drop-out by the time of analysis, the target sample size is 234 patients. .

With this number of patients, there is over 90% power to detect a 5 point difference in mean bowel bother scores between the randomised groups. Assuming a mean bowel bother summary score of 95.0 in surgical patients with a standard deviation of 9.4[21], a difference in mean score of 5.0 will be able to be detected with 152 patients in total (assuming 90% power and a two-sided 5% alpha).

Unless otherwise advised by the IDMC, principal analyses will take place after all PACE A patients have completed a minimum of two years follow-up.

13.2.2 Co-primary endpoints: Patient reported urinary incontinence and bowel bother

Urinary incontinence will be assessed using the ‘number of absorbent pads required per day to control leakage’ question on the EPIC questionnaire. The proportion of patients at two years from the completion of treatment reporting any use of daily pads is of primary interest.

Bowel bother will be assessed using the bowel bother summary score from the EPIC questionnaire. The mean score at two years from the completion treatment is of primary interest. A low bowel bother score indicates more bother.

13.2.3 Secondary Endpoints

13.2.3.1 Freedom from biochemical/clinical failure

The definition of biochemical progression is different for the two treatment groups. For patients receiving surgery, biochemical failure will be defined as PSA>0.2ng/ml. For patients receiving prostate SBRT, PSA failure will be defined as nadir +2ng/ml (nadir is the lowest value recorded after the commencement of radiotherapy). The commencement of androgen deprivation also counts as biochemical failure. Time will be measured from randomisation in both groups. The primary time point of interest is 5 years.

In all cases, PSA failure will be confirmed with a second measurement (>4 weeks from the index measurement) also meeting the criteria for PSA failure.

In addition it is now recognised that after SBRT a benign PSA bounce is seen in up to 20% of patients, usually within the first 2 years, [9, 70, 71]. A benign PSA bounce may also occur with conventional radiotherapy. In some cases the magnitude of the bounce is high enough for the patient to be incorrectly classified as a PSA failure. To prevent this, for patients receiving SBRT or conventional radiotherapy, PSA failure before 24 months will require 3 consecutive rises in PSA resulting in a clinical diagnosis of failure, or commencement of further treatment (eg androgen deprivation therapy). After 24 months, the definition of PSA failure will revert to the Phoenix definition described above (ie nadir+2 ng/ml).

It is recognised that whilst freedom from biochemical/clinical failure is a key secondary outcome measure there is limited power to make conclusions regarding the non-inferiority of SBRT compared to surgery on this endpoint. For example, if the 5 year freedom from biochemical/clinical failure rate is 85% with surgery non-inferiority margins of 12% (HR 1.95) and 11% (HR 1.84) could be ruled out with 80% or 70% power respectively (1-sided 5% alpha). Relaxing the type 1 error rate to 10% would permit margins of 10% or less (HR 1.76; 80% power) and 9% or less (HR 1.66; 70% power) to be ruled out

13.2.3.2 Acute Toxicity

Acute toxicity will be assessed at the end of treatment and for 12 weeks post completion of treatment using CTCAEv4.03 and RTOG scales. Surgical toxicity will be also be assessed using the Clavien toxicity scale prior to discharge and at weeks 2 and 4. Direct comparisons of PACE A and PACE B toxicity will not be possible.

13.2.3.3 Late toxicity

Late toxicity will be assessed using CTCAEv4.03 and RTOG scales measured from any time after the 12 week assessment post-treatment completion. Adverse events of grade 2 or greater experienced at 24 months from treatment is of primary interest.

13.2.3.4 Progression free survival

This will be measured as the first occurrence of biochemical failure, commencement of hormone therapy, local recurrence, pelvic/lymph node recurrence, distant disease or death from any cause. Time will be measured from randomisation. Local progression and pelvic/lymph node progression will be measured as the first occurrence of positive local biopsy following randomisation. Rectal examination is not done routinely during follow up but should be recorded on the eCRF if done. MRI/Ultrasound and biopsies are performed when indicated by rising PSA. Distant disease is defined as a positive result for any of the following: CT/MRI scan showing metastatic disease without new primary; bone scan; choline PET, chest X-ray.

13.2.3.5 Disease specific survival

This will include deaths from prostate cancer only. In general, patients with death recorded as prostate cancer related with no prior progression will be reviewed on a case by case basis. Patients with an unknown cause of death will be assumed to have died from prostate cancer if thye have a previously reported progression, otherwise they will be assumed to have died from other causes. Patients dying from other causes will be censored at date of death. Time will be measured from randomisation.

13.2.3.6 Overall survival

This will include deaths from any cause. Time will be measured from randomisation.

13.2.3.7 Distant progression

This will be measured as the first occurrence of distant disease. Distant disease is defined as a positive result for any of the following: CT/MRI scan showing metastatic disease without new primary; bone scan; choline PET, chest X-ray. Patients who died without progression will be censored at date of death. Time will be measured from randomisation. A sensitivity analysis may be conducted assuming patients reporting a prostate cancer death without prior distant progression have distant disease at the date of death.

13.2.3.8 Commencement of hormone therapy

Date on which anti-androgens or LHRH analogues/antagonists are started or date on which orchidectomy occurs.

13.2.3.9 Acute and late patient reported outcomes

Bladder, bowel and sexual function will be assessed using EPIC-26. Erectile dysfunction will be assessed using IIEF-5. Urinary and bowel incontinence will also be assessed using the IPSS and Vaizey questionnaires respectively. Acute is defined as 12 weeks from the end of treatment and late from any time after the 12 week assessment

13.3 PACE B: Conventional radiotherapy vs prostate SBRT

13.3.1 Sample Size

The sample size is based on a five year freedom from biochemical/clinical failure of 85% in patients receiving conventionally fractionated radiotherapy of 78 Gy in 39 fractions. The aim of the study is to demonstrate non-inferiority of SBRT compared to conventional radiotherapy. Table 8 gives the total number of patients required to demonstrate non-inferiority based on various minimum desirable differences to rule out. A one-sided 5% significance level has been used and an allowance for 10% drop-out at the time of analysis. It was originally anticipated that recruitment will take four years and there will be a staggered start to recruitment as centres open. However, due to the change in sponsorship opening of new centres has been delayed so anticipated recruitment is now 4.5 years. Extending the recruitment period by 6 months allows the sample size to remain unchanged. Revised recruitment predictions took in to account actual recruitment during year 1 and then expects 20% of patients to be recruited in year 2, 30% in years 3 and 4 and 15% of total recruitment in the final 6 months of recruitment.

Unless otherwise advised by the IDMC, principal analyses will take place after the required number of events have been observed or after a minimum of five years follow-up for all patients.

It can be seen that a 6% difference at 5 years (corresponding to a critical hazard ratio of 1.45) could be ruled out with 858 patients randomised in total (80% power). Sample size may be increased if accrual is faster than anticipated, in order to increase the power of the study.

Table 8. Total sample size estimates for PACE B (total number of events required in brackets)

Difference to rule out	Hazard ratio	80% power	90% power
5%	1.373	1224 (269)	1595 (350)
6%	**1.450**	**858 (194)**	1118 (252)
7%	1.529	641 (149)	835 (194)
8%	1.608	500 (119)	652(155)

<u>The target sample size is 858 patients</u>. The decision to close the study to further recruitment on achieving the target sample size will be taken with advice from the Independent Data Monitoring Committee (IDMC).

Toxicity associated with prostate SBRT is also an important endpoint. It is anticipated that 24 months RTOG bladder and/or bowel toxicity of grade 2 or greater will be approximately 10% for patients receiving conventional radiotherapy [88] With 429 patients in each arm there would be 80% power to rule out a 6% difference in toxicity with SBRT i.e. exclude more than 16% toxicity at 24 months with prostate SBRT (non-inferiority, 5% one-sided alpha)

13.3.2 Primary Endpoint: Freedom from biochemical/clinical failure

Biochemical progression after 24 months is defined as an increase in serum PSA of at least 2ng/ml greater than the post-radiotherapy nadir (the lowest PSA to date) confirmed by a second consecutive reading also of at least 2ng/ml greater than the post-treatment nadir. A commencement of androgen deprivation also counts

as biochemical failure. Time will be measured from randomisation. The primary timepoint of interest is 5 years.

As described above, to prevent patients with a benign PSA bounce after radiotherapy being incorrectly classified as PSA failures, PSA failure before 24 months will require 3 consecutive rises in PSA resulting in a clinical diagnosis of failure, or commencment of further treatment (eg androgen deprivation therapy). For this low/intermediate risk population the chance of a true biochemical failure within 2 years of treatment is very low.

13.3.3 Secondary Endpoints

13.3.3.1 Acute toxicity

Acute toxicity will be assessed using RTOG during treatment and using CTCAE v4.03 and RTOG scales for 12 weeks after completing treatment

13.3.3.2 Late toxicity

Late toxicity will be assessed using CTCAE v4.03 and RTOG scales. Any toxicity recorded after the 12 week post-treatment assessment will count as late toxicity.Toxicity at 24 months from treatment will be the time point of primary interest.

13.3.3.3 Progression free survival

This will be measured as the first occurance of biochemical failure, commencement of hormone therapy, local recurrence, pelvic/lymph node recurrence, distant disease or death from any cause. Time will be measured from randomisation. Local progression and pelvic/lymph node progression will be measured as the first occurrence of positive local biopsy following randomisation. Rectal examination is not done routinely during follow up but should be recorded on the eCRF if done. MRI/Ultrasound and biopsies are performed when indicated by rising PSA. Distant disease is defined as a positive result for any of the following: CT/MRI scan showing metastatic disease without new primary; bone scan; choline PET, chest X-ray.

13.3.3.4 Disease-specific survival

This will include deaths from prostate cancer only. In general, patients with death recorded as prostate cancer related with no prior progression will be reviewed on a case by case basis. Patients with an unknown cause of death will be assumed to have died from prostate cancer if thye have a previously reported progression, otherwise they will be assumed to have died from other causes. Patients dying from other causes will be censored at date of death. Time will be measured from randomisation.

13.3.3.5 Overall survival

This will include deaths from any cause. Time will be measured from randomisation.

13.3.3.6 Distant progression

This will be measured as the first occurrence of distant disease. Distant disease is defined as a positive result for any of the following: metastatic disease on CT/MRI; bone scan;choline PET scan; chest X-ray. Patients who died without progression will be censored at date of death. Time

will be measured from randomisation. A sensitivity analysis may be conducted assuming patients reporting a prostate cancer death without prior distant progression have distant disease at the date of death.

13.3.3.7 Commencement of hormone therapy

Date on which anti-androgens, LHRH analogues or antagonists are commenced or date on which orchidectomy occurs.

13.3.3.8 Acute and late patient reported outcomes

Bladder, bowel and sexual function will be assessed using EPIC-26. Erectile dyfunction will be assessed using IIEF-5. Urinary and bowel incontinence will also be assessed using the IPSS and Vaizey questionnaires respectively. Acute is defined as 12 weeks from the end of treatment and late from any time after the 12 week assessment.

13.4 Statistical Analysis (for PACE A and PACE B)

13.4.1 Primary Analysis Population

Analyses of outcome data will be on the basis of intention to treat and therefore include all patients randomised into each study (regardless of ineligibility for study treatment, unwillingness to continue with follow-up visits, withdrawal of consent after randomisation, deviation from allocated treatment and lost to follow-up). However, randomised patients who have not received at least one fraction of radiotherapy (or did not receive surgery if allocated to that group) will not be included in toxicity analyses.

13.4.2 Analysis Methods

PACE A – The primary comparison of patient reported outcomes between surgery and prostate SBRT will be at two years from the completion of treatment. For urinary incontinence, the proportion of patients with any use of asborbent pads will be presented by treatment group. The chi-squared or Fisher's exact test will be used to compare the two groups. For bowel bother, the summary score will be presented as mean and standard deviaton for each treatment group[30]. The t-test will be used to compare the two groups, if data are normally distributed (if not, the Mann-Whitney test will be used). A 5% significance level will be used for both comparisons.

PACE B - The primary comparison will be conventional radiotherapy versus prostate SBRT. Analyses will estimate the size of the treatment effect with a 90% confidence interval for the estimated difference between randomisation groups (equivalent to one-sided 95% confidence interval). The primary analysis of freedom from biochemical/clinical progression will be event driven unless the Independent Monitoring Committee and Trial Steering Committees agree that analysis prior to the target number of events being observed would be mature and robust to have potential to influence clinical practice. Freedom from biochemical/clinical progression will be analysed by the logrank test. Information will be provided on both the absolute and relative treatment effects. Estimates of event rates will be calculated using the Kaplan-Meier method. Primary analyses will be unadjusted. The Cox proportional hazard model will be used to adjust for risk group and important known prognostic factors. Methods to account for non-proportionality will be used if appropriate. The origin time will be taken as the date of randomisation. Patients alive and free of event at the time of analysis and

patients lost to follow-up will be censored at the last available PSA assessment. The primary time-point of interest is 5 years.

In both PACE A and PACE B:

For all time-to-event endpoints (other than freedom from biochemical/clinical failure) analyses will use the logrank test. Hazard ratios will be presented with a 95% confidence interval. Estimates of event rates will be calculated using the Kaplan-Meier method. Principal analyses will be unadjusted. Methods to account for non-proportionality will be used if appropriate. The origin time will be taken as the date of randomisation for efficacy endpoints and date of treatment completion for toxicity endpoints. Patients alive and free of event at the time of analysis and patients lost to follow-up will be censored at the last available assessment. The primary time-point of interest is 5 years.

- Acute and late toxicity will be summarised by the proportions experiencing grade ≥2 side effects with comparisons made (where appropriate) using chi-squared based tests or Fisher's exact test if expected cell frequencies are less than 5. In addition, methods for ordinal data will be used.For acute toxicity, the week 12 assessment post treatment is of specific interest and a formal comparison of grade 2 or greater in each treatment arm will be conducted
- For late toxicity, the 24 month assessment is of specific interest and a formal comparison of grade 2 or greater in each treatment arm will be conducted

The number and percentage of patients with acute toxicity of each grade in each treatment group at each time point will be specified. Late toxicity will be summarised as the number and percentage of each grade in each treatment group at each time point.

Time-to-event analyses will also be conducted for time to first grade 1+, grade 2+ and grade 3+ event. Kaplan-Meier curves (by treatment group) will be presented for time to event data, point estimates (with 95% CIs) will be reported. Patients alive and free of an event at the time of analysis will be censored at last available toxicity assessment. Patients who have died will be censored at date of death.

Standard algorithms will be used to derive scores from and handle missing data in quality of life questionnaires (IPSS, IIEF-5, Vaizey and EPIC-26). Treatment groups will be compared at individual time-points and analyses to account for the longitudinal nature of the data (generalised estimating equations) may be used. To make some adjustment for multiple testing a significance level of 1% will be used for comparisons of quality of life endpoints other than for analysis of the co-primary endpoints in PACE-A.

Further details of analysis methods will be specified in a Statistical Analysis Plan in accordance with ICR-CTSU Standard Operating Procedures.

13.5 Stopping Rules and Interim Analyses

It is planned that an Independent Data Monitoring Committee (IDMC) will meet at approximately 6 monthly intervals to review the accumulating safety and emerging efficacy data.

Once 30 patients have been treated with SBRT on a conventional linac (ie non-Cyberknife systems), the toxicity, acute and late, will be reviewed by the IDMC to ensure there is not an augmented rate of side effects in this cohort. After this, conventional linac SBRT vs Cyberknife SBRT toxicity and outcomes will continue to be monitored by the IDMC separately and together to ensure ongoing safety of this technique.

14 Quality assurance (QA)

14.1 Surgery QA

Surgical workload is the best measure of quality of surgery, and hence a minimum number of procedures per year has been specificed (>20). Sites will be asked to complete a surgical QA form which will be reviewed by a surgical member of the PACE TMG member. In addition, data on surgical margin positivity and postoperative complications will be reviewed by the IDMC to ensure a reasonable level of consistency across all sites.

14.2 Radiotherapy QA

The following QA documents and exercises must be completed by new centres for each radiotherapy treatment arm before commencing recruitment:

- Statement of unit calibration protocol
- Independent beam output audit
- Process document
- Benchmark case (see 14.2.1 below)
- IGRT benchmark test (conventional linac delivery only)
- Prospective individual case reviews will be performed for the first patient randomised to each treatment arm (see 14.2.2 below)

14.2.1 Benchmark Study: All potential sites shall receive, prior to patient enrollment, anonymous electronic patient data sets including CT and MRI images. A treatment plan shall be developed according to the protocol for both SBRT and IMRT, and the plan reviewed by the study team; completion of satisfactory benchmark plans is required prior to patient enrollment.

14.2.2 The first patient for each treatment allocation will undergo pre-treatment review. The treatment plan of the first patient enrolled at each site for each treatment must be reviewed prior to beginning treatment. The study team shall be notified at the time of enrollment of each patient, and of the proposed first treatment date, to assure the team's availability for review. There is the option for contours to be reviewed prior to planning if the centre prefers. After planning is complete, the treating site will make the treatment plan available to the study team site for review. The study team shall complete review within 2 weeks of receipt; treatment will only begin after any necessary corrections are implemented and final plan is approved. In addition, the first intermediate risk case must also be reviewed if the cases reviewed above were both low risk and did not include the seminal vesicles.

14.2.3 Thereafter plans will be reviewed as deemed necessary by the study team.

14.2.4 All outlining should be either performed by or reviewed and approved by the PI at the centre who has been through the pre-trial outlining QA. Since this is a clinical trial and the patient numbers may not be excessive we hope this approach will be acceptable. However, where this is not feasible we recommend the following:

14.2.4.1 The PI should review and approve clinical outlines for the 1st 3 PACE patients recruited by each additional clinician at that centre, after which (assuming these are satisfactory) they are also approved for PACE. Note: Please ensure at least one is an intermediate risk group case, since many

inconsistencies with proximal seminal vesicle outlining have been reported.

14.2.5 Should the PI leave and be replaced, the replacement should perform the PACE benchmark outlining QA to be reviewed by the PACE QA team.

14.2.6 Treatment plan exports

All patient treatment plans will be exported in DICOM format, anonymised, and sent to the RT QA team electronically

15 Ethical and regulatory aspects

15.1 Research Governance

15.1.1 Sponsor responsibilities:

The sponsor of this clinical trial is the Royal Marsden NHS Trust. Responsibilities of participating sites are defined in an agreement between the individual participating site and the Sponsor.

15.2 Trial Administration & Logistics

15.2.1 Site activation:

Before activating the trial, participating sites are required to sign an agreement accepting responsibility for all trial activity which takes place within their site.

Sites may commence recruitment once the site agreement has been signed by all required signatories, the required trial documentation is in place (as specified by ICR-CTSU) and a site initiation (visit or teleconference) has taken place. Site initiation visits will be conducted at sites where the Principal Investigator has requested one or where ICR-CTSU in discussion with the Chief Investigator or Sponsor deems it is appropriate.

15.2.2 Investigator training:

Each centre will complete the comprehensive pre-trial section of the quality assurance programme prior to commencing recruitment, as detailed in section 14. The quality assurance programme will continue throughout the trial, with investigator training as required.

15.2.3 Data acquisition:

Electronic (e) Case Report Forms (CRF) will be used for the collection of trial data. ICR-CTSU will provide guidance to sites to aid the completion of the eCRFs. The Trial Management Group reserves the right to amend or add to the eCRF template as appropriate. Such changes do not constitute a protocol amendment, and revised or additional forms should be used by sites in accordance with the guidelines provided by ICR-CTSU.

15.2.4 Central data monitoring:

Once data has been entered on the eCRF by the site personnel, ICR-CTSU will review it for compliance with the protocol, and for inconsistent or missing data. Should any missing data or data anomalies be found, queries will be raised for resolution by the site.

Any systematic inconsistencies identified through central data monitoring may trigger an on-site monitoring visit.

15.2.5 On-site monitoring:

If a monitoring visit is required, ICR-CTSU will contact the site to arrange the visit. Once a date has been confirmed, the site should ensure that full patient notes of participants selected for source data verification are available for monitoring.

ICR-CTSU staff conducting on-site monitoring will review essential documentation and carry out source data verification to confirm compliance with the clinical trial agreement and trial protocol. If any problems are detected during the course of the monitoring visit, ICR-CTSU will work with the Principal Investigator or delegated individual to resolve issues and determine appropriate action.

15.2.6 Completion of the study and definition of study end date:

The study end date is deemed to be the date of last data capture.

15.2.7 Archiving:

Essential trial documents should be retained according to local policy and for a sufficient period for possible inspection by the regulatory authorities (at least 5 years after the date of last data capture). Documents should be securely stored and access restricted to authorised personnel.

15.3 Patient Protection And Ethical Considerations

15.3.1 Trial approvals:

This trial has been formally assessed for risk by the Sponsor and ICR-CTSU. The trial has received ethical approval from a research ethics committee for multi-centre trials and global R&D approval via the NIHR Coordinated System for gaining NHS Permission. Before entering patients, the Principal Investigator at each site is responsible for submitting Site Specific Information and gaining local Research and Development approval of this protocol.

15.3.2 Trial conduct:

This trial will be conducted according to the approved protocol and its amendments, supplementary guidance and manuals supplied by the Sponsor and in accordance with the Research Governance Framework for Health and Social Care and the principles of GCP.

15.3.3 Informed consent:

Patients should be asked to sign the current main REC approved PACE consent form at trial entry after receiving both verbal and written information about the trial, having been given sufficient time to consider this information. All consent forms must be countersigned by the Principal Investigator or a designated

individual. A signature log of delegated responsibilities, listing the designated individuals and the circumstances under which they may countersign consent forms, must be maintained at the participating site. This log, together with original copies of all signed patient consent forms, should be retained in the Site Investigator File and must be available for inspection. The current main REC approved PACE patient information sheets should be provided in addition to any standard patient information sheets that are provided by the site and used in routine practice.

15.3.4 Patient confidentiality:

Patients will be asked to consent to their full name being collected at registration in addition to their date of birth, hospital number, postcode and NHS number or equivalent to allow linkage with routinely collected NHS data.

Each investigator should keep a separate log of all participants' Trial IDs, names, addresses and hospital numbers. The investigator must retain trial documents (e.g. participants' written consent forms) in strict confidence. The investigator must ensure the participants' confidentiality is maintained at all times.

Representatives of ICR-CTSU, the Sponsor, the site's Research and Development Office and the regulatory authorities may require access to participants' hospital notes for quality assurance purposes. Confidentiality of participants will be maintained at all times and information by which participants could be identified will not be reproduces or disclosed.

15.3.5 Data Protection Act (DPA):

ICR-CTSU will comply with all applicable data protection laws.

15.3.6 Liability

Indemnity for participating NHS hospitals is provided by the usual NHS indemnity arrangements. Each participating site is responsible for ensuring insurance and indemnity arrangements are in place to cover the liability of the Principal Investigator. Inclusion of private patients will be subject to the site ensuring appropriate insurance and indemnity arrangements are in place.

15.4 Financial Matters

This trial is investigator designed and led and has been endorsed by the Clinical Trials Advisory & Awards Committee (CTAAC) of Cancer Research UK. A research grant has been given to the trial Sponsor by Accuray.

In the UK, the trial meets the criteria for R&D support as outlined in the Statement of Partnership on Non-Commercial R&D in the NHS in England. The trial is part of the National Institute for Health Research (NIHR) portfolio. Research Network resources should therefore be made available for the trial to cover UK specific research costs.

16 Study management and oversight

The study will be conducted in line with relevant regulations and will conform to the GCP principles. Three bodies will be set up to ensure the study is managed appropriately:

16.1 Trial management group (TMG)

A Trial Management Group (TMG) will be set up and will include the Chief Investigator, Clinical Co-ordinator, ICR-CTSU Scientific lead, Co-investigators and identified collaborators, the Trial Statistician and the Trial Managers. Principal Investigators and key study personnel will be invited to join the TMG as appropriate to ensure representation from a range of centres and professional groups. Notwithstanding the legal obligations of the Sponsor and Chief Investigator, the TMG have operational responsibility for the conduct of the trial. Where possible, membership will include a lay/consumer representative. The Committee's terms of reference, roles and responsibilities will be defined in a charter issued by ICR-CTSU.

16.2 Independent data monitoring committee (IDMC)

An Independent Data Monitoring Committee (IDMC) will be set up to monitor the progress of the trial and will comprise a Chairman and at least two further members with clinical or statistical expertise (at least one member must be a statistician). Membership of the IDMC will be proposed by the TMG and approved by the TSC.

The IDMC will meet in confidence at regular intervals, and at least annually. A summary of findings and any recommendations will be produced following each meeting. This summary will be submitted to the TMG and TSC, and if required, the main REC.

The IDMC will reserve the right to release any data on outcomes or side-effects through the TSC to the TMG (and if appropriate to participants) if it determines at any stage that the combined evidence from this and other studies justifies it.

The Committee's terms of reference, roles and responsibilities will be defined in a charter issued by ICR-CTSU.

It would be within the remit of the IDMC to monitor toxicity and freedom from biochemical/clinical failure rates and survival rates in the surgery and conventional radiotherapy arms (on which the sample sizes has been calculated) and advise whether the assumptions are valid for emerging data from the trial.

16.3 Independent Trial Steering Committee (TSC)

A Trial Steering Committee (TSC) comprising independent experts in oncology, surgery and statistics will provide high level oversight of the trial. The TSC will monitor progress against recruitment milestones and will advise the TMG on any major protocol amendments. It is anticipated that the TSC will meet at least annually. The Committee's terms of reference, roles and responsibilities will be defined in a charter issued by ICR-CTSU.

17 Study sponsorship

17.1 Study organisation

This is an academically led study sponsored by the Royal Marsden NHS Foundation Trust, London, SW3 6JJ. Statistical analyses will be conducted by ICR-CTSU. Trial Coordination will be performed by ICR-CTSU (a UKCRC registered NCRI cancer clinical trials unit) who will be responsible for the day to day conduct of the trial.

17.2 Contracts

Study sites will enter into a written research agreement with the Royal Marsden NHS Foundation Trust (sponsor) which sets out the responsibilities for study conduct. There is no per patient payment for entering patients into this study.

An additional research agreement between the Sponsor and ICR will define ICR-CTSU's roles and repsonsibilities including those related to central trial co-ordination, database provision, central statistical monitoring, interim analyses/reports for review by the IDMC and principal analysis for presentation/publication.

18 Publication policy

The main trial results will be published in a peer-reviewed journal, on behalf of all collaborators. The manuscript(s) will be prepared by a writing group, consisting of members of the Trial Management Group, and participating clinicians. All participating clinicians will be acknowledged in the publication. Separate primary publications are planned for PACE-A and PACE-B. With the consent of the IDMC and TSC and where this will not compromise the ongoing integrity of the trial, results of toxicity analyses may be published ahead of the primary analysis of efficacy data.

All presentations and publications relating to the trial must be authorised by the Trial Management Group. Authorship of any secondary publications, will reflect the intellectual and time input into these studies.

No Investigator may present or attempt to publish data relating to the PACE trial without prior permission from the Trial Management Group.

19 Appendices

19.1 Appendix 1: Staging

UICC/AJCC 2002 TNM classification

T - Primary tumour

TX	Primary tumour cannot be assessed
T0	No evidence of primary tumour
T1	Clinically inapparent tumour neither palpable nor visible by imaging
T1a	Tumour incidental histological finding in 5% or less of tissue resected
T1b	Tumour incidental histological finding in more than 5% of tissue resected
T1c	Tumour identified by needle biopsy (e.g., because of elevated PSA)
T2	Tumour confined within the prostate*
T2a	Tumour involves one-half of one lobe or less
T2b	Tumour involves more than one-half of one lobe but not both lobes
T2c	Tumour involves both lobes
T3	Tumour extends through the prostatic capsule**
T3a	Extracapsular extension (unilateral or bilateral)
T3b	Tumour invades seminal vesicle(s)
T4	Tumour is fixed or invades adjacent structures other than seminal vesicles: bladder neck, external sphincter, rectum, levator muscles, and/or pelvic wall

N - Regional lymph nodes***

NX	Regional lymph nodes were not assessed
N0	No regional lymph node metastasis
N1	Metastasis in regional lymph node(s)

M - Distant metastasis****

MX	Distant metastasis cannot be assessed (not evaluated by any modality)
M0	No distant metastasis
M1	Distant metastasis
M1a	Non-regional lymph node(s)
M1b	Bone(s)
M1c	Other site(s) with or without bone disease

*Tumour found in one or both lobes by needle biopsy, but not palpable or reliably visible by imaging, is classified as T1c.
** Invasion into the prostatic apex, or into (but not beyond) the prostate capsule, is not classified as T3, but as T2.
***The regional lymph nodes are the nodes of the true pelvis, which essentially are the pelvic nodes below the bifurcation of the common iliac arteries.
Laterality does not effect the N classification.
****When more than one site of metastasis is present, the most advanced category should be used.

Reference: Greene FL, Page DL, Fleming ID et al. (eds). AJCC Cancer Staging Manual, Sixth Edition. Heidelberg Berlin New York: Springer 2002.

19.2 Appendix 2: NCCN risk groups

Low-risk
T1-T2a
Gleason 2-6
PSA <10 ng/ml

Intermediate risk
T2b/c or
Gleason 7 or
PSA 10-20 ng/ml

High risk
T3 or
Gleason 8 or more, or
PSA >20 ng/ml

19.3 Appendix 3 – Expected Adverse Events

The following are possible anticipated treatment related AEs (i.e. expected occurrences) which are not subject to expedited reporting but all such events should be reported in the appropriate section of the CRF UNLESS they fulfil the protocol definition of an SAE.

19.3.1 Surgery Arm

- Bowel strictures
- Ureteric obstruction
- Immediate postoperative urinary incontinence
- Immediate postoperative erectile dysfunction
- Pulmonary embolus/ Deep vein thrombosis
- Greater than 1500 ml intraoperative blood loss
- Return to theatre for bleeding, haematoma or any other reason
- Intraoperative damage to adjacent organ
- Persisting urinary leak that prevents abdominal drain removal
- Ileus lasting greater than three days
- Urinary septicaemia
- Readmission to hospital for operation related complication
- Clinical indication that delays removal of urethral catheter

19.3.2 SBRT and Conventional Radiotherapy Arms

- Urinary toxicities:
    - Urinary frequency/urgency/nocturia
    - Urinary retention
    - Urinary obstruction/strictures
    - Haematuria
    - Cystitis/bladder spasms
    - Urinary incontinence/leakage
    - Pain (prostate, urinary/dysuria)
- GI Toxicities:
    - Pain (rectal, pelvic, abdominal)
    - Diarrhoea
    - Constipation
    - Rectal bleeding/ulcer
    - Fistula
    - Proctitis
    - Bowel obstruction or perforation
- Sexual function
    - Erectile dysfunction
    - Decreased volume of ejaculate/absence of ejaculate
    - Decreased libido
- Dermatology/Skin
    - Rash
    - Hair loss in treatment area
- Bone fractures
- Related to fiducial marker insertion
    - Bleeding
    - Sepsis (urinary and systemic)
    - Pain

19.4 Appendix 4 - Non-UK Safety reporting requirements

The site Principal Investigator or designee is responsible for reporting SAEs to their individual Institutional Review Board (IRB) and/or Institutional Ethics Committee (EC) as per local standards.

The collaborative group in each participating country will report related unexpected SAEs as per their local requirements to IECs and local investigators.

Further Sponsor safety reporting notification requirements will be agreed in the international site agreements.

20 References

1. Ferlay, J., et al., *GLOBOCAN 2008 v1.2, Cancer Incidence and Mortality Worldwide: IARC CancerBase No. 10 [Internet].*
2. CRUK, *Prostate cancer- UK incidence statistics.* Accessed 29th September 2014.
3. Bill-Axelson, A., et al., *Radical prostatectomy versus watchful waiting in early prostate cancer.* N Engl J Med, 2011. **364**(18): p. 1708-17.
4. Hamdy, F.C., et al., *10-Year Outcomes after Monitoring, Surgery, or Radiotherapy for Localized Prostate Cancer.* New England Journal of Medicine, 2016. **375**(15): p. 1415-1424.
5. Dearnaley, D.P., et al., *5 year outcomes of a phase III randomised trial of conventional or hypofractionated high dose intensity modulated radiotherapy for prostate cancer (CRUK/06/016): report from the CHHiP Trial Investigators Group.* ECCO Conference abstract 2015, 2015.
6. Aluwini, S., et al., *Hypofractionated versus conventionally fractionated radiotherapy for patients with prostate cancer (HYPRO): acute toxicity results from a randomised non-inferiority phase 3 trial.* Lancet Oncol, 2015. **16**(3): p. 274-83.
7. Incrocci, L., et al., *Hypofractionated vs Conventionally Fractionated Radiotherapy for Prostate Cancer: 5-year Oncologic Outcomes of the Dutch Randomized Phase 3 HYPRO Trial.* ASTRO annual meeting abstract, 2015.
8. Lee, W.R., et al., *Randomized Phase III Noninferiority Study Comparing Two Radiotherapy Fractionation Schedules in Patients With Low-Risk Prostate Cancer.* Journal of Clinical Oncology, 2016. **34**(20): p. 2325-2332.
9. Meier, R., et al., *CyberKnife Stereotactic Radiosurgery for Organ-Confined Prostate Cancer: Early Toxicity, PSA and Quality of Life Outcomes from a Multi-Center Study.* Presented at 2011 Cyberknife Robotic radiosurgery conference 2011.
10. Fuller, D.B., et al., *Prospective Evaluation of CyberKnife stereotactic Body Radiotherapy for Low-and IntermediateRisk Prostate Cancer: Emulating HDR Brachytherapy Dose Distribution.* Presented at 2011 Cyberknife Robotic radiosurgery conference, 2011.
11. King, C.R., et al., *Stereotactic body radiotherapy for localized prostate cancer: Pooled analysis from a multi-institutional consortium of prospective phase II trials.* Radiother Oncol, 2013.
12. King, C.R., et al., *Health-related quality of life after stereotactic body radiation therapy for localized prostate cancer: results from a multi-institutional consortium of prospective trials.* Int J Radiat Oncol Biol Phys, 2013. **87**(5): p. 939-45.
13. Gonzalez-San Segundo, C., et al., *Radical Prostatectomy Versus External-Beam Radiotherapy for Localized Prostate Cancer: Long-Term Effect on Biochemical Control-In Search of the Optimal Treatment.* Ann Surg Oncol, 2011.
14. Kupelian, P.A., et al., *Comparison of the efficacy of local therapies for localized prostate cancer in the prostate-specific antigen era: a large single-institution experience with radical prostatectomy and external-beam radiotherapy.* J Clin Oncol, 2002. **20**(16): p. 3376-85.
15. Kupelian, P.A., et al., *Radical prostatectomy, external beam radiotherapy <72 Gy, external beam radiotherapy > or =72 Gy, permanent seed implantation, or combined seeds/external beam radiotherapy for stage T1-T2 prostate cancer.* Int J Radiat Oncol Biol Phys, 2004. **58**(1): p. 25-33.

16. Potters, L., et al., *Monotherapy for stage T1-T2 prostate cancer: radical prostatectomy, external beam radiotherapy, or permanent seed implantation.* Radiother Oncol, 2004. **71**(1): p. 29-33.
17. Stephenson, A.J., et al., *Prostate cancer-specific mortality after radical prostatectomy for patients treated in the prostate-specific antigen era.* J Clin Oncol, 2009. **27**(26): p. 4300-5.
18. Pound, C.R., et al., *Natural history of progression after PSA elevation following radical prostatectomy.* JAMA, 1999. **281**(17): p. 1591-7.
19. Novara, G., et al., *Trifecta outcomes after robot-assisted laparoscopic radical prostatectomy.* BJU Int, 2011. **107**(1): p. 100-4.
20. Ficarra, V., et al., *Retropubic, laparoscopic, and robot-assisted radical prostatectomy: a systematic review and cumulative analysis of comparative studies.* Eur Urol, 2009. **55**(5): p. 1037-63.
21. Donovan, J.L., et al., *Patient-Reported Outcomes after Monitoring, Surgery, or Radiotherapy for Prostate Cancer.* New England Journal of Medicine, 2016. **375**(15): p. 1425-1437.
22. van der Wielen, G.J., J.P. Mulhall, and L. Incrocci, *Erectile dysfunction after radiotherapy for prostate cancer and radiation dose to the penile structures: a critical review.* Radiother Oncol, 2007. **84**(2): p. 107-13.
23. Mangar, S.A., et al., *Evaluating the relationship between erectile dysfunction and dose received by the penile bulb: using data from a randomised controlled trial of conformal radiotherapy in prostate cancer (MRC RT01, ISRCTN47772397).* Radiother Oncol, 2006. **80**(3): p. 355-62.
24. Roach, M., 3rd, et al., *Radiation dose-volume effects and the penile bulb.* Int J Radiat Oncol Biol Phys, 2010. **76**(3 Suppl): p. S130-4.
25. Robinson, J.W., S. Moritz, and T. Fung, *Meta-analysis of rates of erectile function after treatment of localized prostate carcinoma.* Int J Radiat Oncol Biol Phys, 2002. **54**(4): p. 1063-8.
26. Treiyer, A., et al., *A single center prospective study: prediction of postoperative general quality of life, potency and continence after radical retropubic prostatectomy.* J Urol, 2011. **185**(5): p. 1681-5.
27. Pinkawa, M. and P. Gontero, *Open to debate. The motion: Radiotherapy for prostate cancer preserves sexual function to a greater extent than nerve sparing radical prostatectomy.* Eur Urol, 2009. **56**(1): p. 212-4.
28. Sanda, M.G., et al., *Quality of life and satisfaction with outcome among prostate-cancer survivors.* N Engl J Med, 2008. **358**(12): p. 1250-61.
29. Rosen, R.C., et al., *Development and evaluation of an abridged, 5-item version of the International Index of Erectile Function (IIEF-5) as a diagnostic tool for erectile dysfunction.* Int J Impot Res, 1999. **11**(6): p. 319-26.
30. Wei, J.T., et al., *Development and validation of the expanded prostate cancer index composite (EPIC) for comprehensive assessment of health-related quality of life in men with prostate cancer.* Urology, 2000. **56**(6): p. 899-905.
31. Dearnaley, D.P., et al., *Phase III pilot study of dose escalation using conformal radiotherapy in prostate cancer: PSA control and side effects.* Br J Cancer, 2005. **92**(3): p. 488-98.
32. Dearnaley, D.P., et al., *The early toxicity of escalated versus standard dose conformal radiotherapy with neo-adjuvant androgen suppression for patients with localised prostate cancer: results from the MRC RT01 trial (ISRCTN47772397).* Radiother Oncol, 2007. **83**(1): p. 31-41.

33. Dearnaley, D.P., et al., *Escalated-dose versus standard-dose conformal radiotherapy in prostate cancer: first results from the MRC RT01 randomised controlled trial.* The Lancet Oncology, 2007. **8**(6): p. 475-487.
34. Dearnaley, D.P., et al., *Escalated-dose versus control-dose conformal radiotherapy for prostate cancer: long-term results from the MRC RT01 randomised controlled trial.* The Lancet Oncology. **15**(4): p. 464-473.
35. Kupelian, P., et al., *Improved biochemical relapse-free survival with increased external radiation doses in patients with localized prostate cancer: the combined experience of nine institutions in patients treated in 1994 and 1995.* Int J Radiat Oncol Biol Phys, 2005. **61**(2): p. 415-9.
36. Pollack, A., et al., *Prostate cancer radiation dose response: results of the M. D. Anderson phase III randomized trial.* Int J Radiat Oncol Biol Phys, 2002. **53**(5): p. 1097-105.
37. Peeters, S.T., et al., *Dose-response in radiotherapy for localized prostate cancer: results of the Dutch multicenter randomized phase III trial comparing 68 Gy of radiotherapy with 78 Gy.* J Clin Oncol, 2006. **24**(13): p. 1990-6.
38. Matzinger, O., et al., *Acute toxicity of curative radiotherapy for intermediate- and high-risk localised prostate cancer in the EORTC trial 22991.* Eur J Cancer, 2009. **45**(16): p. 2825-34.
39. Heidenreich, A., et al. *European Association of Urology: Guidelines on Prostate Cancer*. 2012.
40. Fiorino, C., et al., *Dose-volume effects for normal tissues in external radiotherapy: pelvis.* Radiother Oncol, 2009. **93**(2): p. 153-67.
41. Dasu, A. and I. Toma-Dasu, *Prostate alpha/beta revisited -- an analysis of clinical results from 14 168 patients.* Acta Oncol, 2012. **51**(8): p. 963-74.
42. Miralbell, R., et al., *Dose-fractionation sensitivity of prostate cancer deduced from radiotherapy outcomes of 5,969 patients in seven international institutional datasets: alpha/beta = 1.4 (0.9-2.2) Gy.* Int J Radiat Oncol Biol Phys, 2012. **82**(1): p. e17-24.
43. Marzi, S., et al., *Modeling of alpha/beta for late rectal toxicity from a randomized phase II study: conventional versus hypofractionated scheme for localized prostate cancer.* J Exp Clin Cancer Res, 2009. **28**: p. 117.
44. Tucker, S.L., et al., *Estimation of alpha/beta for Late Rectal Toxicity Based on RTOG 94-06.* Int J Radiat Oncol Biol Phys, 2011.
45. Lloyd-Davies, R.W., C.D. Collins, and A.V. Swan, *Carcinoma of prostate treated by radical external beam radiotherapy using hypofractionation. Twenty-two years' experience (1962-1984).* Urology, 1990. **36**(2): p. 107-11.
46. Collins, C.D., *Radical external beam radiotherapy for localized carcinoma of the prostate using a hypofractionation technique.* Clin Oncol (R Coll Radiol), 1991. **3**(6): p. 358.
47. Livsey, J.E., et al., *Hypofractionated conformal radiotherapy in carcinoma of the prostate: five-year outcome analysis.* Int J Radiat Oncol Biol Phys, 2003. **57**(5): p. 1254-9.
48. Soete, G., et al., *Phase II study of a four-week hypofractionated external beam radiotherapy regimen for prostate cancer: report on acute toxicity.* Radiother Oncol, 2006. **80**(1): p. 78-81.
49. Martin, J.M., et al., *Phase II trial of hypofractionated image-guided intensity-modulated radiotherapy for localized prostate adenocarcinoma.* Int J Radiat Oncol Biol Phys, 2007. **69**(4): p. 1084-9.

50. Yeoh, E.E., et al., *Hypofractionated Versus Conventionally Fractionated Radiotherapy for Prostate Carcinoma: Final Results of Phase III Randomized Trial.* Int J Radiat Oncol Biol Phys, 2010.
51. Yeoh, E.E., et al., *Hypofractionated versus conventionally fractionated radiation therapy for prostate carcinoma: updated results of a phase III randomized trial.* Int J Radiat Oncol Biol Phys, 2006. **66**(4): p. 1072-83.
52. Yoshioka, Y., et al., *High-dose-rate brachytherapy without external beam irradiation for locally advanced prostate cancer.* Radiother Oncol, 2006. **80**(1): p. 62-8.
53. Yoshioka, Y., et al., *Monotherapeutic High-Dose-Rate Brachytherapy for Prostate Cancer: Five-Year Results of an Extreme Hypofractionation Regimen with 54 Gy in Nine Fractions.* Int J Radiat Oncol Biol Phys, 2010.
54. Schour, L., D.J. Demanes, G.A. Altieri, D. Brandt, M. Barnaba, P. Skoolisariyaporn, *High Dose Rate Monotherapy for Prostate Cancer.* Int J Radiat Oncol Biol Phys, 2005. **63**: p. S315.
55. Demanes, D.J., et al., *High-Dose-Rate Monotherapy: Safe and Effective Brachytherapy for Patients with Localized Prostate Cancer.* Int J Radiat Oncol Biol Phys, 2011.
56. Corner, C., et al., *A Phase II study of high-dose-rate afterloading brachytherapy as monotherapy for the treatment of localized prostate cancer.* Int J Radiat Oncol Biol Phys, 2008. **72**(2): p. 441-6.
57. Aluwini, S., et al., *Toxicity and quality of life after high-dose-rate brachytherapy as monotherapy for low- and intermediate-risk prostate cancer.* Radiother Oncol, 2015.
58. Henderson, D.R., A.C. Tree, and N.J. van As, *Stereotactic Body Radiotherapy for Prostate Cancer.* Clin Oncol (R Coll Radiol), 2015.
59. Roach, M., 3rd, et al., *Pretreatment prostate-specific antigen and Gleason score predict the risk of extracapsular extension and the risk of failure following radiotherapy in patients with clinically localized prostate cancer.* Semin Urol Oncol, 2000. **18**(2): p. 108-14.
60. Davis, B.J., et al., *The radial distance of extraprostatic extension of prostate carcinoma: implications for prostate brachytherapy.* Cancer, 1999. **85**(12): p. 2630-7.
61. Chao, K.K., et al., *Clinicopathologic analysis of extracapsular extension in prostate cancer: should the clinical target volume be expanded posterolaterally to account for microscopic extension?* Int J Radiat Oncol Biol Phys, 2006. **65**(4): p. 999-1007.
62. Freeman, D.E. and C.R. King, *Stereotactic body radiotherapy for low-risk prostate cancer: five-year outcomes.* Radiat Oncol, 2011. **6**: p. 3.
63. Katz, A.J., et al., *Stereotactic body radiotherapy for localized prostate cancer: disease control and quality of life at 6 years.* Radiat Oncol, 2013. **8**(1): p. 118.
64. Chen, L.N., et al., *Stereotactic body radiation therapy (SBRT) for clinically localized prostate cancer: the Georgetown University experience.* Radiat Oncol, 2013. **8**: p. 58.
65. Curtis, W., et al., *Relationship of imaging frequency and planning margin to account for intrafraction prostate motion: analysis based on real-time monitoring data.* Int J Radiat Oncol Biol Phys, 2013. **85**(3): p. 700-6.
66. Bittner, N., et al., *Electromagnetic tracking of intrafraction prostate displacement in patients externally immobilized in the prone position.* Int J Radiat Oncol Biol Phys, 2010. **77**(2): p. 490-5.

67. Roach, M., 3rd, et al., *Predicting long-term survival, and the need for hormonal therapy: a meta-analysis of RTOG prostate cancer trials.* Int J Radiat Oncol Biol Phys, 2000. **47**(3): p. 617-27.
68. D'Amico, A.V., et al., *6-month androgen suppression plus radiation therapy vs radiation therapy alone for patients with clinically localized prostate cancer: a randomized controlled trial.* Jama, 2004. **292**(7): p. 821-7.
69. Denham, J.W., et al., *Short-term androgen deprivation and radiotherapy for locally advanced prostate cancer: results from the Trans-Tasman Radiation Oncology Group 96.01 randomised controlled trial.* Lancet Oncol, 2005. **6**(11): p. 841-50.
70. Martinez, A.A., et al., *Lack of benefit from a short course of androgen deprivation for unfavorable prostate cancer patients treated with an accelerated hypofractionated regime.* Int J Radiat Oncol Biol Phys, 2005. **62**(5): p. 1322-31.
71. Jones, C.U., et al., *Radiotherapy and short-term androgen deprivation for localized prostate cancer.* N Engl J Med, 2011. **365**(2): p. 107-18.
72. Williams, S., et al., *Androgen Deprivation Therapy Plus Hypofractionated Prostate Radiotherapy—Are We Castrating the Radiobiological Advantage?* Int J Radiat Oncol Biol Phys, 2008. **72**(1): p. S73-74.
73. NCCN. *National Comprehensive Cancer Network - Clinical Practice Guidelines in Oncology: Prostate Cancer. Version 1.2016*. 2015 27/10/2015]; Available from: http://www.nccn.org/professionals/physician_gls/pdf/prostate.pdf.
74. Valicenti, R.K., et al., *Does hormone therapy reduce disease recurrence in prostate cancer patients receiving dose-escalated radiation therapy? An analysis of radiation therapy oncology group 94-06.* Int J Radiat Oncol Biol Phys, 2011. **79**(5): p. 1323-9.
75. Krauss, D., et al., *Lack of benefit for the addition of androgen deprivation therapy to dose-escalated radiotherapy in the treatment of intermediate- and high-risk prostate cancer.* Int J Radiat Oncol Biol Phys, 2011. **80**(4): p. 1064-71.
76. Barry, M.J., et al., *The American Urological Association symptom index for benign prostatic hyperplasia. The Measurement Committee of the American Urological Association.* J Urol, 1992. **148**(5): p. 1549-57; discussion 1564.
77. Vaizey, C.J., et al., *Prospective comparison of faecal incontinence grading systems.* Gut, 1999. **44**(1): p. 77-80.
78. Bolzicco, G., et al., *A single-center study of 100 consecutive patients with localized prostate cancer treated with stereotactic body radiotherapy.* BMC Urol, 2013. **13**(1): p. 49.
79. Katz, A., *Five-year Biochemical Control Rates for Stereotactic Body Radiotherapy for Organ Confined Prostate Cancer: A Multi-institutional Pooled Analysis.* Oral presentation at ASTRO annual meeting 2012, 2012. **84**(3 Suppl 1): p. s147-148.
80. Sobin, L. and C. Wittekind, *TNM classification of malignant tumours 6th edition.* New York: Wiley-Liss, 2002.
81. King, C.R., A. Lo, and D.S. Kapp, *Testicular dose from prostate cyberknife: a cautionary note.* Int J Radiat Oncol Biol Phys, 2009. **73**(2): p. 636-7; author reply 637.
82. Santanam, L., et al., *Standardizing naming conventions in radiation oncology.* Int J Radiat Oncol Biol Phys, 2012. **83**(4): p. 1344-9.
83. (NRIG), N.R.I.G.R., *Stereotactic Body Radiotherapy. Clinical review of the evidence for SBRT.* 2011.
84. King, C.R., et al., *Long-term outcomes from a prospective trial of stereotactic body radiotherapy for low-risk prostate cancer.* Int J Radiat Oncol Biol Phys, 2012. **82**(2): p. 877-82.

85. Katz, A.J., et al., *Stereotactic body radiotherapy for organ-confined prostate cancer.* BMC Urol, 2010. **10**: p. 1.
86. Nuttall, M.C., et al., *Threshold volumes for urological cancer surgery: a survey of UK urologists.* BJU Int, 2004. **94**(7): p. 1010-3.
87. Dindo, D., N. Demartines, and P.A. Clavien, *Classification of surgical complications: a new proposal with evaluation in a cohort of 6336 patients and results of a survey.* Ann Surg, 2004. **240**(2): p. 205-13.
88. Zelefsky, M.J., et al., *Improved clinical outcomes with high-dose image guided radiotherapy compared with non-IGRT for the treatment of clinically localized prostate cancer.* Int J Radiat Oncol Biol Phys, 2012. **84**(1): p. 125-9.

The PACE Trial (Prostate Advances in Comparative Evidence)

Statistical Analysis Plan

PACE B - Acute Toxicity Sub-study

Version 5.1

23.05.19

This statistical analysis plan is based on protocol version 8

Dated: 16th November 2016

ISRCTN Number: 17627211

REC reference number:

CRUK (other funder) number: CRUKE/12/025

ICR-CTSU Number:

ClinicalTrials.gov reference number: NCT01584258

Sponsor reference number:

Author: Douglas Brand

23.05.19

The current draft version of SAP if prior to principal analysis of primary endpoint, any previous draft versions used for formal presentation, i.e. peer reviewed conference posters/presentations prior to the principal analysis, and any final version(s) of the SAP will be stored in the Statistical Section of the Trial Master File

This statistical analysis plan is a framework to guide statistical analysis and may be supplemented by additional and exploratory analyses. Trial statisticians reserve the right to amend analysis methods as appropriate after discussion with the ICR-CTSU Scientific Lead.

For final versions only:
This statistical analysis plan has been approved by the following personnel:

PACE ICR Clinical Fellow: Douglas Brand

Signed:__________________________________ Date: __/__/____

Trial Statistician: Vicki Hinder/Clare Griffin

Signed: ___________________________ Date: __/__/____

Signed: ___________________________ Date: __/__/____

ICR-CTSU Scientific Lead: Prof Emma Hall

Signed: ___________________________ Date: __/__/____

Chief Investigator: Dr Nicholas van As

Signed: ___________________________ Date: __/__/____

Document history

Version	Date	Changes made (including justifications)
1.0	12/12/17	N/A
2.0	05/03/18	Power calculation added<br>Primary and secondary endpoints added<br>At request Prof Hall
2.1	18/10/18	Amended primary endpoint analysis to be chi-square unless assumptions not met
3.0	08/11/18	Clarified purpose. Included information regarding the IDMC recommendations. Added Cyberknife exploratory work
3.1	15/11/18	Amended alpha to one sided following discussion with Dr van As.
3.11	15/11/18	Added re exploratory presentation of data comparing the different durations of each modality.
3.12	22/11/18	Reversion to two-sided test per discussion NvA and EH
4.0	04/12/18	Following examination of data, an exceptional change in co-primary endpoints made. Instead of emergent (above baseline) RTOG G2+ GU and GI toxicity, it will be G2+ RTOG toxicity for GI and GU without reference to baseline. This is due to 10% of baseline forms being done after fiducial insertion, with more SBRT patients receiving fiducials and this introducing a strong risk of bias.
5.0	16/05/19	Major changes to include specific p-value adjusted hypothesis testing of the secondary endpoints of interest. This is to bring in line with the methods used on CHHiP QoL analysis. Specific outline of probable content of paper included in the appendix 3.
5.1	23/05/19	Mann-whitney to compare baseline distributions

		for RTOG and CTCAE. Minor amendments to references. MCIDs more clearly defined. Missing data approach better defined, without bias to direction of effect.

Contents

1.0 Introduction 6
1.1 Trial design 6
1.2 Study population 7
2.0 Trial objectives 9
3.0 Randomisation/recruitment procedures 9
4.0 Endpoints 11
5.0 Sample size and power 11
6.0 Data completeness and consistency 13
6.1 Patient flow through trial 14
6.2 Compliance with assessments 15
6.3 Consistency of reporting 15
6.4 Missing data 15
7.0 Analysis methods 16
7.1 Analysis populations 16
7.2 Baseline characteristics 16
7.3 Analyses of defined endpoints 17
7.4 Treatment compliance 20
7.5 Exploratory analyses 20
Dosimetry data will be obtained for as many PACE B patients as possible.
Ideally for each patient the following data items will be collected: 20
8.0 General considerations 21
8.1 Subgroup analyses 21
9.0 Independent Data Monitoring Committee (IDMC) and interim analyses 22
10.0 Analysis Programs 22
11.0 Analysis program locations 22
References 20

1.0 Introduction

The PACE (Prostate Advances in Comparative Evidence) trial had two initial main comparisons (A & B), with a third (PACE C) commencing soon. PACE B compares experimental stereotactic body radiotherapy (SBRT) to conventionally fractionated or moderately hypofractionated radiotherapy (CMFHRT) radiotherapy for the treatment of localised prostate cancer. This statistical analysis plan describes the methods that will be used to analyse acute toxicity data from the trial, defined as the toxicity data accruing up to the 12 week follow-up point. This sub-study will hereafter be referred to as the PACE B Acute Toxicity Study. This document is referred to as the PACE B Acute Toxicity SAP. The SAP can be viewed at request, subject to approval by the CI and ICR-CTSU.

The purpose of this SAP is to outline clearly the planned methods for analysis and avoid data-driven approaches. Any derivations from this analysis plan will be documented in the statistical analysis report. Procedures for monitoring data accuracy and data entry quality are provided in the PACE trial main statistical monitoring plan.

1.1 Trial design

The PACE (Prostate Advances in Comparative Evidence) trial is an international, multi-centre, randomised, open label, phase III, non-inferiority clinical trial addressing the comparative effectiveness of treatments for early prostate cancer. There are two halves to the trial, with both halves comparing SBRT (36.25Gy in 5 fractions) against, respectively, surgery [PACE A] or CMFHRT [PACE B]. This analysis will focus on the patients recruited to PACE B, for whom the recruitment completed in December 2017.

The primary endpoint is biochemical/clinical progression free survival. Biochemical progression is defined, using Phoenix consensus guidelines, as PSA nadir + 2 ng/mL, confirmed on a second PSA reading. For patients within 24 months of radiotherapy, 3 successive PSA rises are required due to the PSA bounce phenomenon. Clinical progression was defined as time of commencing androgen deprivation therapy. PACE B will need to recruit 858 patients (1:1 randomisation) in order to have 80% power to exclude 6% or more absolute detriment (non-inferiority margin) to biochemical or clinical progression at 5 years. An assumption is that 85% of control patients will be biochemical/clinical progression free at 5 years.

Secondary endpoints are (*Adapted from PACE Protocol Version 8, 16th November 2016*):

1. Clinician reported acute toxicity, assessed using CTCAEv4.03, RTOG
2. Clinician reported late toxicity, assessed using CTCAEv4.03 and RTOG

3. Patient reported outcomes and quality of life assessment for all treatment patients: Assessed using International Index of Erectile Function-5 (IIEF-5), International Prostate Symptom Score (IPSS), Vaizey score, Expanded Prostate Index Composite-26 (EPIC-26).
4. Disease-specific and overall survival.
5. Progression-free survival– radiographic, clinical or biochemical evidence of local or distant failure.
6. Commencement of androgen deprivation therapy (LHRH analogues, anti-androgens, orchidectomy).

No formal interim analysis will be performed. The Independent Data Monitoring Committee (IDMC) reviews safety and efficacy data approximately 6 monthly. The standard linear accelerator (LINAC) based SBRT patients were examined after 30 patients had been treated to ensure there was not excess toxicity, with no recommendation to halt trial given by the IDMC.

This analysis will concern the evaluation of acute toxicity, as covered in the secondary endpoints 1 and 3. This will be analysed according to treatment received (not intention-to-treat), with only patients receiving at least one fraction of SBRT or CFMHRT radiotherapy included.

The control treatment in PACE B is CFMHRT with daily fractionation. In the initial protocol, this was mandated as 78 Gy in 39 fractions, however following the results of the CHHiP trial [1], a protocol amendment was made to allow 62 Gy in 20 fractions, over at least 27 days, as a control treatment option. The experimental treatment is SBRT, delivered as 36.25 Gy in 5 fractions, delivered either daily or every other day (max 14 days total treatment time). The treatment could be delivered on either Cyberknife (Accuray, USA) system or on a standard LINAC gantry.

For both arms, it was recommended to start radiotherapy within 8 weeks of randomisation and strictly not more than 12 weeks. No patients were treated with androgen deprivation therapy. For all patients undergoing radiotherapy, image guidance fiducials (3 or more) were strongly advised, bowel preparation was strongly advised, and partial bladder filling was recommended. All patients had a planning CT scan prior to radiotherapy and it was strongly advised to also perform a planning MRI scan (without endorectal coil).

1.2 Study population

All patients in the PACE B trial are eligible for this acute toxicity analysis, provided they received at least a single fraction of radiotherapy, either CFMHRT or SBRT. In the unlikely event that a patient started one form of radiotherapy (SBRT or CFMHRT) and then switched to the other, they will be excluded from this analysis. Reasons for all exclusions will be clearly documented.

The inclusion and exclusion criteria are otherwise those of the main PACE trial:

Per PACE Protocol Version 8 (16th November 2016):

1.2.1 Inclusion Criteria

1.2.1.1 Histological confirmation of prostate adenocarcinoma with a minimum of 10 biopsy cores taken within 18 months of randomisation.

1.2.1.1.1 This requirement for biopsy within 18 months of randomisation may be omitted (unless clinically indicated) if the patient has become a candidate for radical treatment (e.g. due to patient choice or PSA/MRI progression) while being followed up in an active surveillance programme. The patient's most recent biopsy must satisfy all other relevant PACE trial eligibility criteria. Patients progressing on active surveillance will be considered as having intermediate risk disease, and treated accordingly.

1.2.1.2 Gleason score ≤ 3+4

1.2.1.3 Men aged ≥18 years

1.2.1.4 Clinical and/or MRI stage T1c –T2c, N0-X, M0-X (TNM 6th Edition)

1.2.1.5 PSA ≤ 20 ng/ml

1.2.1.6 Pre-enrolment PSA must be completed within 60 days of randomisation

1.2.1.7 Patients belong in one of the following risk groups according to the National Comprehensive Cancer Network (www.nccn.org):

1.2.1.7.1 Low risk: Clinical stage T1-T2a and Gleason ≤ 6 and PSA < 10 ng/ml, or

1.2.1.7.2 Intermediate risk includes any one of the following:

- Clinical stage T2b orT2c
- PSA 10-20 ng/ml or
- Gleason 3+4

1.2.1.8 WHO performance status 0 – 2

1.2.1.9 Ability of the research subject to understand and the willingness to sign a written informed consent document

1.2.2 Exclusion Criteria

1.2.2.1 Clinical stage T3 or greater

1.2.2.2 Gleason score ≥ 4 + 3

1.2.2.3 High-risk disease defined by National Comprehensive Cancer Network (www.nccn.org)

1.2.2.4 Previous malignancy within the last 2 years (except basal cell carcinoma or squamous cell carcinoma of the skin), or if previous malignancy is expected to significantly compromise 5-year survival

1.2.2.5 Prior pelvic radiotherapy

1.2.2.6 Prior androgen deprivation therapy (including LHRH agonists and antagonists and anti-androgens)

1.2.2.7 Any prior active treatment for prostate cancer. Patients previously on active surveillance are eligible if they continue to meet all other eligibility criteria.

1.2.2.8 Life expectancy <5 years

1.2.2.9 Bilateral hip prostheses or any other implants/hardware that would introduce substantial CT artefacts

1.2.2.10 Medical conditions likely to make radiotherapy inadvisable e.g. inflammatory bowel disease, significant urinary symptoms

1.2.2.11 For patients having fiducials inserted. Anticoagulation with warfarin/ bleeding tendency making fiducial placement or surgery unsafe in the opinion of the clinician (see section 11, Treatment).

1.2.2.12 Participation in another concurrent treatment protocol for prostate cancer

2.0 Study objectives

The objective of this PACE B Acute toxicity study will be to compare acute toxicity data (up to week 12 post treatment) by treatment received (per protocol) for PACE B trial participants.

The primary endpoints for this study will be worst grade RTOG G2+ bowel toxicity and worst grade RTOG G2+ bladder toxicity during this period.

Secondary objectives will be to assess differences between SBRT and CFMHRT :

- Baseline, worst, worst exceeding baseline, 12 week scores of all of the following [differences from this in square brackets]:
- RTOG GI G2+
- RTOG GU G2+
- RTOG GI G3+
- RTOG GU G3+
- CTCAE GI G2+
- CTCAE GU G2+
- CTCAE GI G3+
- CTCAE GU G3+
- IPSS total
- IPSS QoL
- IPSS severity distributions [at timepoints collected]
- EPIC-26 Urinary Incontinence Subdomain Score [plus minimal clinically important difference (8 point reduction at any time relative to baseline)]

- EPIC-26 Urinary Obstructive Subdomain Score [plus minimal clinically important difference (6 point reduction at any time relative to baseline)]
- EPIC-26 Urinary Bother
- EPIC-26 Bowel Subdomain Score [plus minimal clinically important difference (5 point reduction at any time relative to baseline)]
- EPIC-26 Bowel Bother
- EPIC-26 Sexual Subdomain Score [plus minimal clinically important difference (11 point reduction at any time relative to baseline)]
- IIEF
- Vaizey

n.b. Minimal clinically important differences for EPIC-26 defined per Skolarus et al [2]

Exploratory analysis

- Compare toxicities between Cyberknife and non-Cyberknife treated patients for patients receiving SBRT [per IDMC request].
- Examine effect of other rational possible predictors on this toxicity, including:
    - International effect (UK&Ireland vs Canada)
    - Learning effect (by centre volume of PACE patients)
    - Margin effects
        - Absolute margins used
        - Deviations from protocol margins
    - Dosimetry information. (Reported dose-volume constraints)
        - Potentially incorporate as binary predictor (planned to protocol, not planned to protocol)
    - Fiducials vs non-fiducials
    - Other plausible predictors (If any)
    - Alpha-blockers/cholinergics at diagnosis
- Dosimetric analyses of individual patient DICOMs in the study, with reference to resultant toxicities, including analysis of differences in contouring,

3.0 Randomisation/recruitment procedures

Central randomisation is performed at ICR-CTSU, via telephone for UK sites and fax for non-UK sites. There is a 1:1 random allocation, by computer generated random permutated block, between CFMHRT and SBRT.

The randomisation will be stratified by:

- Treatment centre
- Risk group (Low, Intermediate)

Treatment allocation is open label.

4.0 Endpoints

Co-Primary Endpoints

- Worst grade RTOG G2+ for bowel toxicity (GI)
- Worst grade RTOG G2+ for bladder toxicity (GU)
    - For each of these, a patient fulfils the endpoint if they have:
        1. GU or GI toxicity at grade 2 or more in follow-up (during and up to 12 weeks post RT treatment)

Secondary Endpoints

All secondary endpoints will be calculated from commencement of RT to 12 weeks post RT

- Graphically report data at baseline and up to 12 weeks follow-up for (details below in graphical section):
    - RTOG -GI & GU
    - CTCAE -GI & GU
    - IPSS – each subdomain and total score. Plus categories by time
    - EPIC-26 – by domain. EPIC bowel and urine bother scores
    - IIEF
    - Vaizey
- Comparison between CFMHRT and SBRT for secondary objectives in **2.0 Study Objectives**

5.0 Sample size and power

The analysis of acute toxicity was not considered as part of the sample size calculations for the main PACE trial. The best comparator for expected acute toxicity in the control arm is the PROFIT trial [3]. This compared 78Gy in 39 fractions to 60 Gy in 20 fractions, both delivered as daily treatments. Similar to PACE B, androgen deprivation therapy was not permitted. The CHHiP trial (mentioned earlier) is also a reasonable comparator, although it permitted higher risk patients and androgen deprivation was administered. The control arm of HYPRO, another trial of the Hypofractionation era, is also of interest, treating patients with 78 Gy / 39# [4]. The table below summarises the relevant data available for cumulative acute toxicity.

**Acute Toxicity**	PROFIT (without ADT)		CHHiP (with ADT)			HYPRO (+/- ADT)
RTOG G2+	78 Gy / 39 #	60 Gy / 20#	74 Gy / 37 #	60 Gy / 20#	57 Gy / 19#	78 Gy / 39 #
Bowel	10.5%	16.7%	25%	38%	38%	31.2 %
Bladder	31%	30.9%	46%	49%	46%	57.8%

Based on data from ICR-CTSU, the proportion of patients in PACE B receiving the two different acceptable conventional treatment arms are:

- 62 Gy / 20# = 90-95%
- 78 Gy / 39# = 5-10%

Therefore, accounting for the above and the disparate nature of available data, the estimates of acute RTOG G2+ cumulative toxicity in the conventional arm of PACE B will be:

- Bowel = 25%
- Bladder = 40%

These are weighted closer to findings in the PROFIT trial, which had more similar patient risk groups (intermediate) and did not allow ADT.

Power calculations were performed using Stata 15: (Table below)
power twoproportions [control proportion] [test proportion] , n(871) alpha(0.025)

- Note alpha set at 2.5% (two-way) to split a total alpha 0.05 across the two endpoints of bowel and bladder.

Power Calculation	Conventional	SBRT		
G2+ RTOG	Proportion	Proportion 1	Proportion 2	Proportion 3
Bowel	**0.25**	0.30	0.34	**0.35**
**POWER**		27%	75%	**83%**
Bladder	**0.40**	0.45	0.50	**0.51**
**POWER**		22%	76%	**84.5%**

Final selections for acute G2+ cumulative RTOG acute toxicity are made to preserve an 80% power (or more), with two way alpha of 0.025 for each endpoint:

- Bowel: 83% power to detect 10% increase in toxicity from 25% control arm
- Bladder: 84.5% power to detect 11% increase in toxicity from 40% control arm.

6.0 Data completeness and consistency

Data completeness and quality will be summarised descriptively, discussing measures taken to clean the data, monitoring checks performed on the data and the findings of these. The Central Statistical Monitoring Plan outlines the data consistency and accuracy checks routinely carried our prior to analyses.

6.1 Patient flow through trial

For the acute toxicity study analysis, patient flow through the trial will be presented using a CONSORT diagram as below:

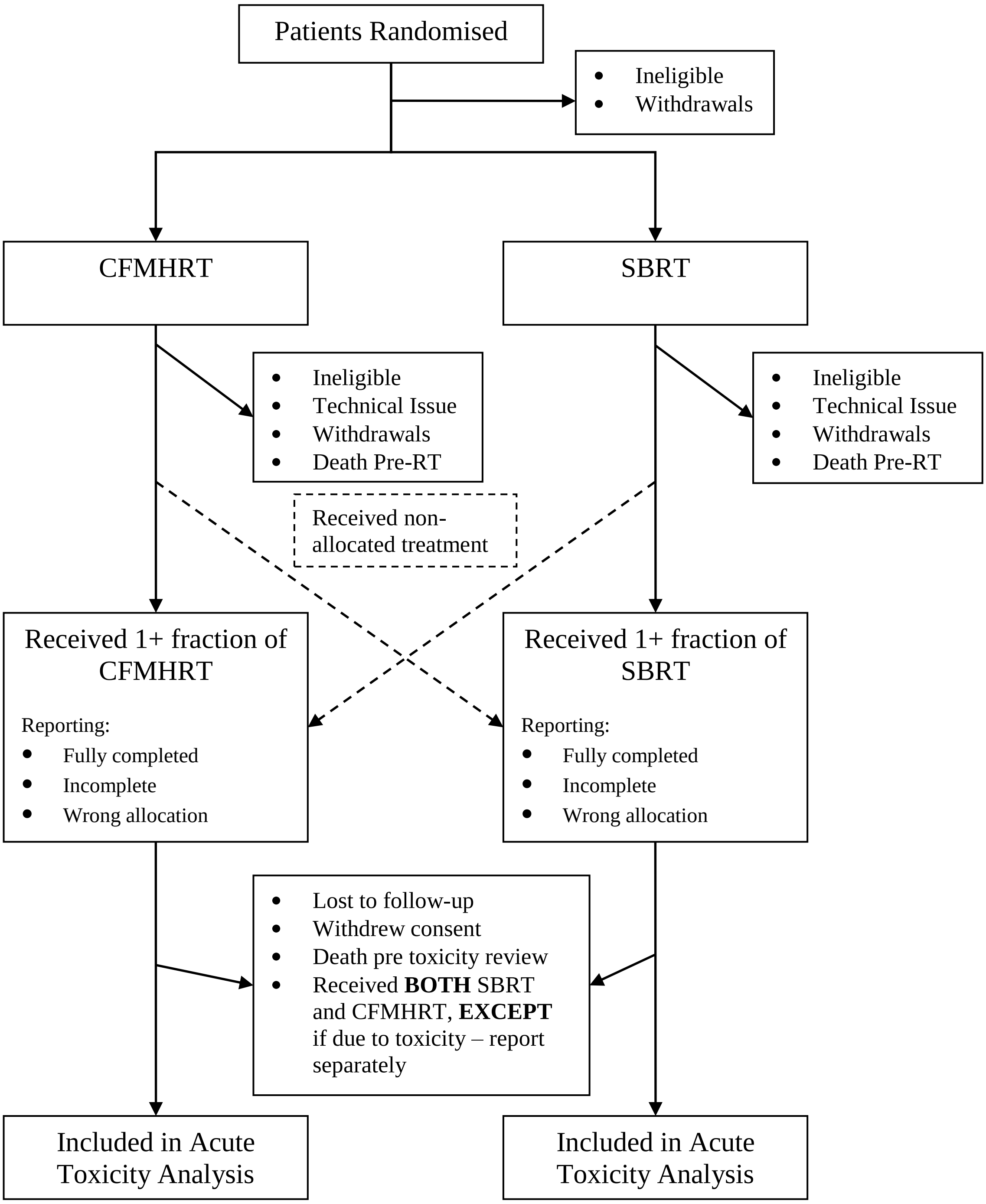

6.2 Compliance with assessments

Compliance with the radiotherapy treatment protocols and acute toxicity follow-up assessments will be reported as part of the acute toxicity study. Treatment protocol compliance will be visually identifiable in the CONSORT diagram (see section 6.1). Where there appear to be differences in compliance between arms, sensitivity analysis will be performed, with reference to date of CRF returns, should the quality of submitted data enable this. Returns rates for all forms will be reported, by per protocol assignment.

6.3 Consistency of reporting

In the event of non-returned CRFs, ICR-CTSU will contact participating centres in order to maximise return rate. In the unusual event (given electronic reporting) of two forms being returned for a single time point, then for each question, the worse outcome score of the two forms will be used in analysis. Forms that have not been dated will be assumed to have been completed on the closest previous clinical assessment date. The handling of any other inconsistencies in data, that data cleaning and querying have not resolved will be recorded in the statistical analysis report.

6.4 Missing data

Any missing data will be requested by the PACE trial manager/data manager. Levels of missing data will be summarised by form type. Should any patterns emerge then these will be examined. It is expected that the majority of missing data will be missing at random and therefore, it is not expected that imputation methods will be used for individual patients or variables.

Should there be a substantial (e.g. 10%) difference in missing data seen between the two arms then a sensitivity analysis will be performed, restricting to those patients with 1 or fewer missing data.

EPIC-26 is the only PRO questionnaire with specific minimum domain completion fraction for the domain sub-score to be valid:

- Urinary Obstructive = 4/4, 100% of items
- Urinary Incontinence = 4/4, 100% of items
- Bowel = 5/6, 83% of items
- Sexual = 5/6, 83% of items
- Hormonal = 4/5, 80% of items

7.0 Analysis methods

7.1 Analysis populations

This PACE acute toxicity analysis will include all patients who received at least a single fraction of radiotherapy. As this is a toxicity assessment, analysis will be performed by treatment received, rather than by intention to treat. Patients receiving both conventional and SBRT fractions of radiotherapy will be excluded from this analysis, by identification from deviation forms and radiotherapy delivery forms.

Patients will then be included in the analysis of each endpoint/timepoint if they have completed the relevant follow-up assessments/questionnaires. In the case of the analysis of EPIC-26, domain scores will be void unless the completion fraction meets the cut-points as stated in section 6.4.

7.2 Baseline characteristics

Baseline Data Collected:

- Age (40-49, 50-59, 60-69, 70-79, 80+)
- Family history of prostate cancer (yes, no)
- WHO Performance status (0, 1, 2, 3+)
- PSA [ng/mL] – (0-5, 5-10, 10-15, 15-20)
- Testosterone [µmol/L] – (<1.5, ≥1.5)
- DRE (T1c, T2a, T2b, T2c) {**non-mandatory**}
- TRUS (T1c, T2a, T2b, T2c) {**non-mandatory**}
- MRI T Stage (T1c, T2a, T2b, T2c)
- MRI N Stage (N0, N1, NX)
- MRI M Stage (M0, M1, MX)
- Prostate Volume [cc]
- Gleason Score (≤3+2, 3+3, 3+4, ≥4+3)
- Percentage Positive Cores [Positive Cores / Total Cores]
- Total length of cores [mm] {**non-mandatory**}
- Total linear extent positive [mm] {**non-mandatory**}
- Concomitant medications:
    - Alpha blockers (yes, no)
    - Aspirin (yes, no)
    - Statins (yes, no)
    - Anti-cholinergics for bladder symptoms (yes, no)

Square brackets [] show units of measurement

Round brackets () show categories for categorical data

All baseline characteristics will be tabulated by treatment group. It is not planned to conduct formal tests between the groups as, by virtue of the randomisation, the demographics and characteristics should be well balanced between the four groups. However, if, by chance, there appears to be a large difference between the treatment groups with regard to a particular baseline variable a formal test (e.g. chi squared test) will be performed.

For nominal/ordinal data (round brackets show categories), percentages will be reported by group. Median average age will also be presented for age, alongside distribution by grouping. For T-stage, the highest value of the DRE, TRUS and MRI will be reported. Median average will be reported for continuous data. Interquartile range and range will also be presented.

7.3 Analyses of defined endpoints

Primary Analysis (Alpha 0.05 two-sided, split as 0.025 per comparison)

- Chi-square test will be performed (Unless assumptions failure thus requires Fisher's exact test) for:
  - Worst bowel RTOG G2+ acute toxicity
  - Worst bladder RTOG G2+ acute toxicity
- Comparing conventional to SBRT. Alpha 0.025 for each comparison.
- Proportions and corresponding confidence intervals will also be presented

Secondary Analyses (Significant p-value = 0.001)

1. RTOG GI baseline (mann-whitney)
2. RTOG GI G2+ at 12 weeks (chi-square – compare proportions)
3. RTOG GI G2+ worst exceeding baseline (chi-square – compare proportions)
4. RTOG GU baseline (mann-whitney)
5. RTOG GU G2+ at 12 weeks (chi-square – compare proportions)
6. RTOG GU G2+ worst exceeding baseline (chi-square – compare proportions)
7. RTOG GI G3+ worst (chi-square – compare proportions)
8. RTOG GI G3+ worst exceeding baseline (chi-square – compare proportions)
9. RTOG GI G3+ at 12 weeks (Chi-square – compare proportions)
10. RTOG GU G3+ worst (chi-square – compare proportions)
11. RTOG GU G3+ worst exceeding baseline (chi-square – compare proportions)
12. RTOG GU G3+ at 12 weeks (Chi-square – compare proportions)

13. CTCAE GU baseline (mann-whitney)
14. CTCAE GU G2+ worst (chi-square – compare proportions)
15. CTCAE GU G2+ worst exceeding baseline (chi-square – compare proportions)
16. CTCAE GU G2+ at 12 weeks (Chi-square – compare proportions)
17. CTCAE GU G3+ worst (chi-square – compare proportions)
18. CTCAE GU G3+ worst exceeding baseline (chi-square – compare proportions)
19. CTCAE GU G3+ at 12 weeks (Chi-square – compare proportions)
20. CTCAE GI baseline (mann-whitney)
21. CTCAE GI G2+ worst (chi-square – compare proportions)
22. CTCAE GI G2+ worst exceeding baseline (chi-square – compare proportions)
23. CTCAE GI G2+ at 12 weeks (Chi-square – compare proportions)
24. CTCAE GI G3+ worst (chi-square – compare proportions)
25. CTCAE GI G3+ worst exceeding baseline (chi-square – compare proportions)
26. CTCAE GI G3+ at 12 weeks (Chi-square – compare proportions)
27. IPSS total baseline (Mann-whitney – compare scores)
28. IPSS total worst (Mann-whitney – compare worst total scores)
29. IPSS total worst change (Mann-whitney – compare worst total scores worsening)
30. IPSS total 12 week (Mann-whitney – compare scores)
31. IPSS QoL baseline (Mann-whitney – compare scores)
32. IPSS QoL worst (Mann-whitney – compare worst QoL scores)
33. IPSS QoL worst change (Mann-whitney – compare worst QoL drop)
34. IPSS QoL 12 week (Mann-whitney – compare scores)
35. IPSS Categories: Baseline (Chi-square test for trend)
36. IPSS Categories: Worst (Chi-square test for trend)
37. IPSS Categories: 12-weeks (Chi-square test for trend)
38. EPIC-26 Urinary Incontinence baseline (Mann-whitney – compare scores)
39. EPIC-26 Urinary Incontinence worst (Mann-whitney – compare worst QoL scores)
40. EPIC-26 Urinary Incontinence worst change (Mann-whitney – worst drop cf baseline)
41. EPIC-26 Urinary Incontinence 12 week (Mann-whitney – compare scores)
42. EPIC-26 Urinary Incontinence MCID drop Any time – Chi-square
43. EPIC-26 Urinary Incontinence MCID drop 12 week – Chi-square
44. EPIC-26 Urinary Obstructive baseline (Mann-whitney – compare scores)
45. EPIC-26 Urinary Obstructive worst (Mann-whitney – compare worst QoL scores)
46. EPIC-26 Urinary Obstructive worst change (Mann-whitney – worst drop cf baseline)
47. EPIC-26 Urinary Obstructive 12 week (Mann-whitney – compare scores)
48. EPIC-26 Urinary Obstructive MCID drop Any time – Chi-square

49. EPIC-26 Urinary Obstructive MCID drop 12 week – Chi-square
50. EPIC-26 Urinary Bother Baseline – Mann whitney
51. EPIC-26 Urinary Bother Worst – Mann whitney
52. EPIC-26 Urinary Bother Worst change – Mann whitney (cf baseline)
53. EPIC-26 Urinary Bother 12 weeks– Mann whitney
54. EPIC-26 Bowel baseline (Mann-whitney – compare scores)
55. EPIC-26 Bowel worst (Mann-whitney – compare scores)
56. EPIC-26 Bowel worst change cf baseline (Mann-whitney – compare scores)
57. EPIC-26 Bowel 12 week (Mann-whitney – compare scores)
58. EPIC-26 Bowel MCID drop Any time – Chi-square
59. EPIC-26 Bowel MCID drop 12 week – Chi-square
60. EPIC-26 Bowel Bother Baseline – Mann whitney
61. EPIC-26 Bowel Bother Worst – Mann whitney
62. EPIC-26 Bowel Bother Worst change – Mann whitney (cf baseline)
63. EPIC-26 Bowel Bother 12 weeks– Mann whitney
64. EPIC-26 Sexual baseline (Mann-whitney – compare scores)
65. EPIC-26 Sexual worst (Mann-whitney – compare scores)
66. EPIC-26 Sexual worst cf baseline (Mann-whitney – compare scores)
67. EPIC-26 Sexual 12 week (Mann-whitney – compare scores)
68. EPIC-26 Sexual MCID drop Any time – Chi-square
69. EPIC-26 Sexual MCID drop 12 week – Chi-square
70. EPIC-26 Hormonal baseline (Mann-whitney – compare scores)
71. EPIC-26 Hormonal worst (Mann-whitney – compare scores)
72. EPIC-26 Hormonal worst cf baseline (Mann-whitney – compare scores)
73. EPIC-26 Hormonal 12 week (Mann-whitney – compare scores)
74. EPIC-26 Hormonal MCID drop Any time – Chi-square
75. EPIC-26 Hormonal MCID drop 12 week – Chi-square
76. IIEF baseline (Mann-whitney – compare scores)
77. IIEF 12 week (Mann-whitney – compare scores)
78. Vaizey Total baseline (Mann-whitney – compare scores)
79. Vaizey Total worst (Mann-whitney – compare scores)
80. Vaizey Total worst cf baseline (Mann-whitney – compare scores)
81. Vaizey 12 week score (Mann-whitney – compare scores)

N.b. Fisher's exact will be used if assumptions of Chi-Square not met.

7.4 Treatment compliance

Treatment compliance will be reported in the CONSORT diagram, indicating how many patients went on to receive at least one fraction of their assigned radiotherapy treatment arm. Additionally it will indicate the number of patients failing to complete the intended course and for what reasons.

7.5 Exploratory analyses

Compare toxicities between Cyberknife and non-Cyberknife treated patients for patients receiving SBRT [per IDMC request]:

1. The margins set and achieved for different techniques – It was felt that SBRT - conventional LINAC was the hardest technique to achieve the margins required.

2. Centre effect – some sites may have more experience with techniques, may be reporting CTCAE and RTOG differently.

3. There may be a learning curve, therefore changes in toxicity patterns over time may be seen.

Differences will be examined by:

- Chi-square (or Fishers) for worst GU and GI RTOG G2+ toxicity
- Examination of effect in other data collected:
  - CTCAE
  - EPIC-26
  - IPSS

Logistic Regression Model

- Treatment platform
- 1 week vs 2 week administration
- International effect (UK&Ireland vs Canada)
- Learning effect (by centre volume of PACE patients)
- Margin effects
  - Absolute margins used
  - Deviations from protocol margins
- Dosimetry information. (Reported dose-volume constraints)
  - Potentially incorporate as binary predictor (planned to protocol, not planned to protocol)
- Fiducials vs non-fiducials
- Alpha-blockers/cholinergics at diagnosis
- Other biologically plausible predictors (If any)

Additional presented data for hypothesis generation

- Graph of RTOG against time, separated by:
    - 1 week SBRT
    - 2 week SBRT
    - 4 week CFMHRT
    - 8 week CFMHRT
- Tabular presentation of worst RTOG, CTCAE, Worst EPIC GI & GU subdomains, Worst IPSS
    - Separated by:
    - 1 week SBRT
    - 2 week SBRT
    - 4 week CFMHRT
    - 8 week CFMHRT

Dosimetry data will be obtained for as many PACE B patients as possible. Ideally for each patient the following data items will be collected.:

- Planning CT DICOM files
- Planning MRI DICOM files (if performed)
    - DICOM Registration Files where MRI employed
- Dose Cube data (DICOM Dose)
- DICOM Planning File
- DICOM Structure Sets

Acceptability of structure contouring for normal organs will be checked, with re-contouring if needed. The data can then be used to generate finalised Dose Volume Histogram (DVH) files. These DVH files can be used to make predictions about the expected toxicity of PACE, based upon normal tissue alpha/beta ratios and time factor estimates derived from the CHHiP data. This will make use of an EQD2 corrected LKB model, potentially incorporating time factor for recovery. This will be done for at least bladder and rectal endpoints. Other exploratory analyses of the methods of contouring and dosimetry may undertaken as appropriate.

8.0 General considerations

PACE-B complete recruitment in late December 2017. All patients have therefore reached the required duration of follow-up to collect 12 week post-RT forms.

8.1 Subgroup analyses

See section 7.5 exploratory analyses

9.0 Independent Data Monitoring Committee (IDMC) and interim analyses

From PACE protocol version 8:
"It is planned that an Independent Data Monitoring Committee (IDMC) will meet at approximately 6 monthly intervals to review the accumulating safety and emerging efficacy data.

Once 30 patients have been treated with SBRT on a conventional linac (ie non-Cyberknife systems), the toxicity, acute and late, will be reviewed by the IDMC to ensure there is not an augmented rate of side effects in this cohort. After this, conventional linac SBRT vs Cyberknife SBRT toxicity and outcomes will continue to be monitored by the IDMC separately and together to ensure ongoing safety of this technique."

The trial has not been halted following the 30 patient review and no interim issues with safety (toxicity) have been raised to date.

IDMC approval of release of the PACE-B acute toxicity data ahead of primary analysis will be required. The IDMC will asked to consider potential impact of knowledge of acute PACE-B toxicity data by investigators on the continued integrity of PACE-A (open to recruitment) and follow-up/reporting of late toxicity in PACE B.

Per the IDMC Recommendations, we are examining:

1. The margins set and achieved for different techniques – It was felt that SBRT - conventional LINAC was the hardest technique to achieve the margins required.

2. Centre effect – some sites may have more experience with techniques, may be reporting CTCAE and RTOG differently.

3. There may be a learning curve, therefore changes in toxicity patterns over time may be seen.

10.0 Analysis Programs

Analysis will be conducted using Stata version 15 (or prior versions). Exploratory analyses may require additional programs, such as R or MATLAB.

11.0 Analysis program locations

All programs will be stored in the analyses folder for PACE on the ICR-CTSU server. Only the PACE trial statistician(s), PACE ICR Clinical Fellow, ICR-CTSU IT staff, Director and Deputy Directors of ICR-CTSU will be able to see the analysis folder. Programmes will be stored under the type of analysis e.g. Acute Toxicity Analysis. All official analysis reports that are to be circulated externally of ICR-CTSU will be password protected. Hard copies of reports will be stored securely in the statistical section of the PACE trial master file held in a locked fire proof cupboard with restricted access.

References

[1] D. Dearnaley *et al.*, "Conventional versus hypofractionated high-dose intensity-modulated radiotherapy for prostate cancer: 5-year outcomes of the randomised, non-inferiority, phase 3 CHHiP trial," *Lancet Oncol.*, vol. 17, no. 8, pp. 1047–1060, Jun. 2016.

[2] T. A. Skolarus *et al.*, "Minimally important difference for the expanded prostate cancer index composite short form," *Urology*, vol. 85, no. 1, pp. 101–105, Jan. 2015.

[3] C. N. Catton *et al.*, "Randomized Trial of a Hypofractionated Radiation Regimen for the Treatment of Localized Prostate Cancer," *J. Clin. Oncol.*, vol. 35, no. 17, pp. 1884–1890, 2017.

[4] S. Aluwini *et al.*, "Hypofractionated versus conventionally fractionated radiotherapy for patients with prostate cancer (HYPRO): Acute toxicity results from a randomised non-inferiority phase 3 trial," *Lancet Oncol.*, vol. 16, no. 3, pp. 274–283, 2015.

Appendix 1
Summary of the Routinely Collected Clinician Reported Outcome Measures

Data Collection Timepoints

Pre-treatment
CTCAE **and**
RTOG Bladder and Bowel

During Treatment
RTOG Bladder and Bowel **only** at:
- Week 2,4,6,8 for CFMHRT radiotherapy arm
- **OR**
- Last fraction only for SBRT arm

Post-Treatment
CTCAE **and**
RTOG Bladder and Bowel **at**
- Week 2,4,8,12 post treatment
- Month 3,6,9
- Annually thereafter to 10 years follow-up

Clinician Reported Outcome Metrics

1) Urinary

a. CTCAE Genito-Urinary Scores
   i. Haematuria (0-5)
   ii. Pain/Dysuria (0-3)
   iii. Frequency (0-2)
   iv. Incontinence (0-3)
   v. Urinary Retention (0-5)
   vi. Urgency (0-2)
b. RTOG Genito-urinary
   i. Cystitis
   ii. Haematuria
   iii. Urethral Stricture

2) Bowel

a. CTCAE Gastrointestinal Scores
   i. Colitis (0-5)
   ii. Constipation (0-5)
   iii. Diarrhoea (0-5)
   iv. GI Fistula (0-5)
   v. Nausea (0-3)
   vi. Proctitis (0-5)
   vii. GI Haemorrhage Anus/rectum (0-5)
   viii. Rectal Pain (0-2)
b. RTOG Gastrointestinal

i. Diarrhoea
ii. Proctitis
iii. Rectal-anal Stricture
iv. Rectal Ulcer
v. Bowel Obstruction

3) Sexual

a. CTCAE
   i. Erectile dysfunction (0-3)

4) Hormonal/General

a. CTCAE
   i. Hot flushes (0-3)
   ii. Pain (0-3) (Specified)
   iii. Fatigue (0-3)
   iv. Anorexia (0-5)
   v. Weight loss (0-3)
   vi. Radiation Dermatitis (0-3)

Appendix 2
Summary of the Routinely Collected Patient Reported Outcome Measures

Data Collection Timepoints

All proformas have been collected at:
- Pre-randomisation
- Week 2,4,8,12 post treatment (acute)
- Month 6, 9 ,12 post treatment
- Annually thereafter to year 5

Patient Reported Outcome Metrics

1) Urinary

a. EPIC-26 Urinary Incontinence Domain – *Need all 4 or score as missing data*
   i. EPIC 26.23 How often leaked urine (1-5) [0,25,50,75,100]
   ii. EPIC 26.26 Urine control (1-4) [0,33,67,100]
   iii. EPIC 26.27 Pads per day (0-3) [100,67,33,0]
   iv. EPIC 26.28 Dripping/leaking urine (0-4) [100,75,50,25,0]

b. EPIC-26 Urinary Obstructive Domain – *Need all 4 or score as missing data*
   i. EPIC 26.29 Pain/Burning or urination (0-4) [100,75,50,25,0]
   ii. EPIC 26.30 Bleeding on urination (0-4) [100,75,50,25,0]
   iii. EPIC 26.31 Weak Stream/Incomplete emptying (0-4) [100,75,50,25,0]
   iv. EPIC 26.33 Frequent daytime urination (0-4) [100,75,50,25,0]

c. EPIC-26 Urinary Non-subscale (Over last 4 weeks)
   i. EPIC 26.34 Overall urine function problem (1-5) [100,75,50,25,0]

d. I-PSS Composite Score (0-35)
   i. Incomplete emptying (0-5)
   ii. Frequency <2 hours (0-5)
   iii. Intermittency (0-5)
   iv. Urgency (0-5)
   v. Weak stream (0-5)
   vi. Straining (0-5)
   vii. Nocturia (0-5)

e. IPSS Quality of Life Score (0-6)

2) Bowel

a. EPIC-26 Bowel Domain – *Need 5 or score as missing data*
   i. EPIC 26.49 Urgency of bowel movement (0-4) [100,75,50,25,0]

ii. EPIC 26.50 Inc. frequency of bowel movements (0-4) [100,75,50,25,0]
iii. EPIC 26.52 Loss of bowel control (0-4) [100,75,50,25,0]
iv. EPIC 26.53 Bloody stools (0-4) [100,75,50,25,0]
v. EPIC 26.54 Abdominal/pelvic/rectal pain (0-4) [100,75,50,25,0]
vi. EPIC 26.55 How big a problem is bowel habits (1-5) [100,75,50,25,0]

b. Vaizey Score Composite (0-24)
   i. Incontinence solid stool (0-4)
   ii. Incontinence of liquid stool (0-4)
   iii. Incontinence of gas (0-4)
   iv. Alteration in Lifestyle (0-4)
   v. Wear pad or plug (0=no, 2=yes)
   vi. Constipating medications (0=no, 2=yes)
   vii. Inability to defer defaecation 15 mins (0=no, 4=yes)

3) Sexual

a. EPIC-26 Sexual Domain – *Need 5 or score as missing data*
   i. EPIC 26.57 Ability to have an erection (1-5) [0,25,50,75,100]
   ii. EPIC 26.58 Ability to orgasm (1-5) [0,25,50,75,100]
   iii. EPIC 26.59 Quality of erections (1-4) [0,33,67,100]
   iv. EPIC 26.60 Frequency of erections (1-5) [0,25,50,75,100]
   v. EPIC 26.64 Ability to function sexually (1-5) [0,25,50,75,100]
   vi. EPIC 26.68 How big a problem is sexual function (1-5) [100,75,50,25,0]

b. IIEF-5 Composite Score (5-25)
   i. Confidence of erection (1-5)
   ii. How often erections suitable for penetrative sex (1-5)
   iii. How often maintain erection after penetration (1-5)
   iv. How difficult to maintain erection to completion (1-5)
   v. How frequently sex satisfactory (1-5)

4) General/Hormonal

a. EPIC-26 Hormonal Domain – *Need 4 or score as missing data*
   i. EPIC 26.74 Hot flushes (0-4) [100,75,50,25,0]
   ii. EPIC 26.75 Breast tenderness/enlargement (0-4) [100,75,50,25,0]
   iii. EPIC 26.77 Depression (0-4) [100,75,50,25,0]
   iv. EPIC 26.78 Lack of Energy (0-4) [100,75,50,25,0]
   v. EPIC 26.79 Change in body weight (0-4) [100,75,50,25,0]

Appendix 3
Plan of Results to Report in Paper

Results Section Plan – By Paragraph

1. Trial Details
    a. Men, centres, dates
    b. CONSORT – explanation of exceptional SBRT + CFMHRT patient to per protocol
        i. **Figure 1** CONSORT Flow Diagram

2. Baseline/Disease/Treatment Characteristics
    a. **Table 1**. By Per Protocol.
        i. Age, Ethnic origin, FHx Prostate ca, WHO PS, NCCN risk, T-score, Gleason, Pre-treatment PSA, Pre-treatment testosterone, Prostate volume, con meds
    b. **Supplementary Table 1**. By Per Protocol
        i. Fid marks, fid mark numbers, RT method, IGRT method, overall treatment times, margins
    c. Brief text explanation of Supp table 1 key points (quicker, more fiducials, smaller margins, more non co-planar in SBRT)

3. Return Rates
    a. Brief comment on return rates for each instrument (perhaps a single number average for each).
    b. Supplementary Table 2. RTOG Return Rates
    c. Supplementary Table 3. CTCAE Return Rates
    d. Supplementary Table 4. EPIC Return Rates
    e. Supplementary Table 5. IPSS Return Rates
    f. Supplementary Table 6. IIEF-5 Return Rates
    g. Supplementary Table 7. Vaizey Return Rates

4. Primary Endpoint Analysis + Other RTOG
    a. **Figure 2, Panel A** RTOG GI toxicity G1+,2+,3+ (point prevalence, all patients) vs time
    b. **Figure 2, Panel B** RTOG GU toxicity G1+,2+,3+ (point prevalence, all patients) vs time
    c. **Supplementary Figure 1.** RTOG GI and GU toxicity separated as 1 week SBRT vs 2 week SBRT vs 4 week MHRT vs 7.8 week CFRT.
    d. Text: Worst acute G2+ proportions GI compared by chi-square (interpreted to sig p-value 0.025)
    e. Text: Worst acute G2+ proportions GU compared by chi-square (interpreted to sig p-value 0.025)
    f. **Supplementary Table 8**. Split by per protocol analysis:
        i. Worst RTOG toxicities (not referencing baseline)
            1. G2+ G3+ GI compared by chi-square (n.b G2+ is also the primary comparison)
            2. G2+ G3+ GU compared by chi-square (n.b G2+ is also the primary comparison)
    g. **Supplementary Table 9.** Split by per protocol analysis:
        i. Worst RTOG toxicities (above baseline) – exclude those without baseline
            1. G2+ G3+ GI compared by chi-square
            2. G2+ G3+ GU compared by chi-square

ii. Baseline RTOG
1. GI – compare by mann-whitney
2. GU – compare by mann-whitney
iii. 12 week RTOG
1. GI – compare G2+ and G3+ by chi square
2. GU – compare G2+ and G3+ by chi square

5. CTCAE
a. **Figure 3, Panel A** CTCAE GI toxicity G1+,2+,3+ (point prevalence, all patients) vs time
b. **Figure 3, Panel B** CTCAE GU toxicity G1+,2+,3+ (point prevalence, all patients) vs time
c. In text: state p-values of worst G2+ and G3+ GI and GU CTCAE comparisons (Chi-square)
d. **Supplementary Table 10** - Worst Acute CTCAE GI Toxicity Items
i. **Composite**
ii. Individual items: Anal Pain, Colitis, Constipation, Diarrhoea, Diverticulitis, Fecal incontinence, Fistula, GI Pain, Haemorrhoids, GI haemorrhage , Proctitis, GI Unspecified, Rectal Prolapse
e. **Supplementary Table 11** - Worst Acute CTCAE GU Toxicity Items
i. **Composite**
ii. Individual items: Bladder Spasm, Cystitis, Haematuria, Prostatic Obstruction, Urinary Frequency, Urinary incontinence, Urinary retention, Urinary urgency
f. **Supplementary Table 11** - Worst Acute CTCAE GI Toxicity Items ABOVE BASELINE
i. **Composite**
ii. Individual items: Anal Pain, Colitis, Constipation, Diarrhoea, Diverticulitis, Fecal incontinence, Fistula, GI Pain, Haemorrhoids, GI haemorrhage, Proctitis, GI Unspecified, Rectal Prolapse
g. **Supplementary Table 12** - Worst Acute CTCAE GU Toxicity Items ABOVE BASELINE
i. **Composite**
ii. Individual items: Bladder Spasm, Cystitis, Haematuria, Prostatic Obstruction, Urinary Frequency, Urinary incontinence, Urinary retention, Urinary urgency
h. Supplementary Table 13 – CTCAE GI Baseline
i. Composite – compare distributions by mann-whitney
ii. Individual (as above)
i. Supplementary Table 14 – CTCAE GU Baseline
i. Composite – compare distributions by mann-whitney
ii. Individual (as above)
j. Supplementary Table 15 – CTCAE GI 12 week
i. Composite - compare distributions by chi-square
ii. Individual (as above)
k. Supplementary Table 16 – CTCAE GU 12 week
i. Composite - compare distributions by chi-square
ii. Individual (as above)

6. EPIC-26 all By 5 subdomains (UI, UO, Bowel, Sexual, Hormonal) *n.b. urine overall bother is separate – outside the subdomain scores, but bowel overall bother is within bowel subdomain*
a. **Figure 4** — mean change in score from baseline with confidence intervals + separate urine QoL (not included in subdomain)
b. **Supplementary Figure 2** – mean scores with confidence intervals
c. **Supplementary Table 17** 5x +urine bother Average, CI for baseline, worst, 12-week scores and comparison by Mann-Whitney

d. **Supplementary Table 18** 5x + urine bother Average, CI for worst change from baseline and comparison by Mann-Whitney
e. **Supplementary table 19** – Proportions experiencing clinically important differences at any time.
f. **Supplementary table 20** – Proportions experiencing clinically important differences at 12 weeks
g. **Supplementary table 21** – Table showing urinary and bowel bother question distributions at 0,4,12 weeks. – I.e. comparative to CHHiP

7. IPSS
    a. **Figure 5** – by question + total + QoL (10 plots) – change from baseline with confidence intervals
    b. **Supplementary Figure 3** - by question + total + QoL (10 plots) – average scores at each timepoint with confidence intervals
    c. **Supplementary Figure 4** – Stacked bar charts showing IPSS severity category by time.
    d. Supplementary Table 22 – IPSS categories – baseline, worst, 12 weeks with chi-square test (for trend) for the distribution of severity grades at each time point between SBRT and CFMHRT
    e. Test: Mann Whitney x3 for IPSS total, at baseline, worst, 12 week
    f. Test: Mann Whitney x3 for IPSS QoL, at baseline, worst, 12 week
    g. Test: Mann Whitney for IPSS worst score change from baseline
    h. Test: Mann Whitney for IPSS worst QoL change from baseline

8. IIEF-5
    a. Text. Score average and CI at 0, 12 weeks. Compare by mann whitney at each timepoint
    b. Text. Change in IIEF-5 from baseline average and CI. Compare by Mann Whitney

9. Vaizey
    a. **Supplementary Table 22**. Score average and CI at 0,4,12 weeks. Compare 0 and 12 weeks by Mann Whitney
    b. Text. **Compare worst Vaizey** – Mann-Whitney
    c. Text. **Compare worst Vaizey score change from baseline** – Mann-Whitney
